# An Updated Review on the Synthesis and Antibacterial Activity of Molecular Hybrids and Conjugates Bearing Imidazole Moiety

**DOI:** 10.3390/molecules25215133

**Published:** 2020-11-04

**Authors:** Renzo Rossi, Maurizio Ciofalo

**Affiliations:** 1 Dipartimento di Chimica e Chimica Industriale, University of Pisa, Via G. Moruzzi, 3, I-56124 Pisa, Italy; 2 Dipartimento di Scienze Agrarie, Alimentari e Forestali, University of Palermo, Viale delle Scienze, Edificio 4, I-90128 Palermo, Italy

**Keywords:** imidazoles, molecular hybrids, molecular conjugates, antibacterials, antibiotic resistance, synthesis, bioactivity, antibiotics

## Abstract

The rapid growth of serious infections caused by antibiotic resistant bacteria, especially the nosocomial ESKAPE pathogens, has been acknowledged by Governments and scientists and is one of the world’s major health problems. Various strategies have been and are currently investigated and developed to reduce and/or delay the bacterial resistance. One of these strategies regards the design and development of antimicrobial hybrids and conjugates. This unprecedented critical review, in which our continuing interest in the synthesis and evaluation of the bioactivity of imidazole derivatives is testified, aims to summarise and comment on the results obtained from the end of the 1900s until February 2020 in studies conducted by numerous international research groups on the synthesis and evaluation of the antibacterial properties of imidazole-based molecular hybrids and conjugates in which the pharmacophoric constituents of these compounds are directly covalently linked or connected through a linker or spacer. In this review, significant attention was paid to summarise the strategies used to overcome the antibiotic resistance of pathogens whose infections are difficult to treat with conventional antibiotics. However, it does not include literature data on the synthesis and evaluation of the bioactivity of hybrids and conjugates in which an imidazole moiety is fused with a carbo- or heterocyclic subunit.

## 1. Introduction

Serious infections caused by antibiotic resistant bacteria, especially the six nosocomial ESKAPE pathogens *Enterococcus faecium*, *Staphylococcus aureus*, *Klebsiella pneumoniae*, *Acinetobacter baumannii*, *Pseudomonas aeruginosa*, and *Enterobacter* spp., are one of the world’s major healthcare problems in the 21st century and are a cause of morbidity and mortality [1]. Overconsumption and misuse of antibiotics and exposure to infections in hospitals has caused the emergence of multi-drug resistant bacteria many of which, according to the World Health Organization (WHO) list, are Gram-negative pathogens [2]. In fact, their structure consists of a protective extra outer membrane that antibiotics have great difficulty going through. Gram-positive bacteria lack this important layer.

In order to reduce and/or delay bacterial resistance, various strategies have been investigated and developed. One of these concerns the development of new antibacterial drugs [3,4] and the structural modification of existing antibiotics, but the studies in this area are very expensive and time consuming. Another effective strategy involves the use of the combination therapy, i.e., the use of two or more drugs to restore or to increase the efficacy of both drugs against the bacterial pathogens that are resistant to ordinary antibiotics. However, none of the expected benefits of following this strategy have thus far been observed in in vivo studies [5]. A third clinically employed strategy concerns the deactivation of the mechanism of resistance through the use of a combination of a β-lactam antibiotic with a β-lactamase inhibitor adjuvant. Finally, another strategy to which much attention has been paid in recent years, presenting a great opportunity for progress, concerns design and development of antimicrobial hybrids [6] and conjugates. According to Klahn’s definition [7], antimicrobial hybrids are molecules that contain two discrete functional elements, both having antibacterial activity, which can be linked through a spacer. Due to the dual targeting, resistance development can be significantly impaired, the pharmacokinetic properties can be better compared to combination therapies with the single antibacterial elements, and the antibacterial activity is often greater than the sum of the antibacterial activity of each element. On the other hand, still according to Klahn [7], in antibacterial conjugates, a single functional moiety controls the accumulation of the other part of the conjugate by mediating an active transport into the bacterial cell or blocking the efflux from it and stimulating the accumulation of the second moiety that acts as an antibiotic through antimicrobial peptides, cell penetrating peptides, lipopeptides or siderophore vectors. Unfortunately, in some recent papers, this classification of antibacterial agents in hybrids and conjugates has not been followed and the antibacterial substances in which a single pharmacophore is connected to a non-bacterial subunit through a spacer or linker have been named both as antibacterial hybrids and conjugates. Nevertheless, in this unprecedented critical review we have defined an antibiotic hybrid as a synthetic combination of two or more covalently linked pharmacophores belonging to an established agent known to elicit an antibacterial effect.

This review, with 261 references, in which we testified our continuing interest in the synthesis and bioactivity of imidazole derivatives [8,9,10,11,12,13,14,15], aims to summarise and comment on the main results obtained from the end of 1900s until the end of February 2020 in studies conducted by numerous international research groups on the synthesis and evaluation of the antibacterial properties of imidazole-based molecular hybrids and conjugates in which their discrete functional elements are directly covalently linked or are connected through a linker or spacer. The review, in which significant attention was paid to summarise the strategies used to overcome the antibiotic resistance of pathogens whose infections are difficult to treat with conventional antibiotics, was organised in the following main sections: (i) heterocyclic conjugates and hybrids bearing nitroimidazole moiety; (ii) coumarin/imidazole hybrids and conjugates; (iii) furanchalcone/imidazole hybrids; (iv) hybrids based on benzofuran, quinazolinone, and imidazolium moieties; (v) 1*H*-imidazoles containing azetidinone derivatives; (vi) pyrrole/imidazole and indole/imidazole hybrids; (vii) pyrazole/imidazole hybrids; (viii) 1,2,3-triazole/imidazole hybrids and conjugates; (ix) hybrids of imidazole derivatives and 5-membered heterocycles containing oxygen and nitrogen; (x) 1,8-naphthalimide/imidazole hybrids; (xi) bis-imidazoles; (xii) pyridine/imidazole hybrids; (xiii) quinoline/imidazole hybrids; (xiv) pyrimidine/imidazole hybrids; (xv) addendum.

However, this review did not include the synthesis and evaluation of the bioactivity of molecular hybrids and conjugates in which imidazole moieties are fused with carbo- or hetero-cyclic subunits or contain only (un)functionalised acyclic groups. Furthermore, no literature data were reported on the synthesis of imidazole containing hybrids and conjugates for which antimicrobial assessments were not carried out.

Occasionally, the original biological data and their standard errors have been rounded off to provide readers with comparable and more statistically consistent results. For the same reason, whenever possible, MIC and (sometimes) IC_50_ values have been reported on a molar basis, regardless of the data format in the original papers.

## 2. Heterocyclic Conjugates and Hybrids Bearing Nitroimidazole Moiety

**Figure 1:**
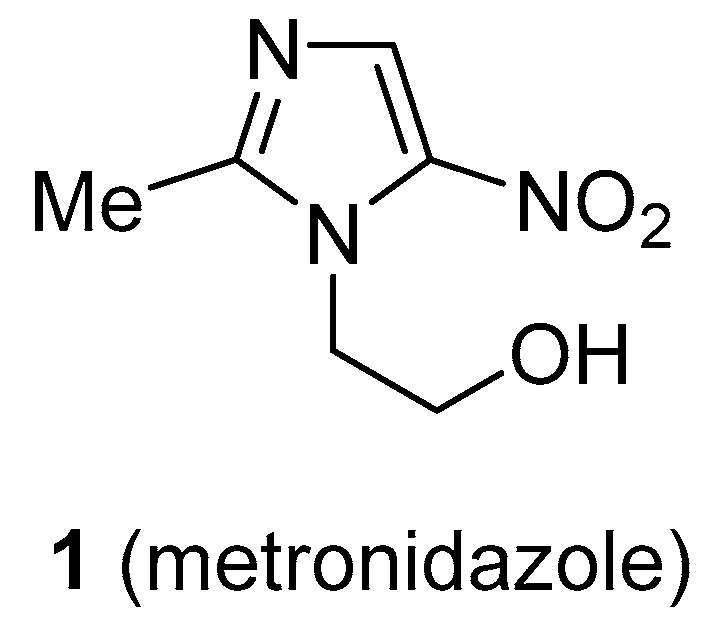
Structure of metronidazole.

Nitroimidazole derivatives are a class of antimicrobial drugs that are used as effective therapeutic agents for treatment of infections caused by Gram-negative and Gram-positive bacteria and protozoa such as *Giardia*, *Lamblia*, and *Entamoeba histolytica* [16]. Metronidazole [2-(2-methyl-5-nitro-1*H*-imidazol-1-yl)ethan-1-ol, Flagyl^®^, Pfizer] (**1**) (Figure 1) is an antibiotic used to treat a wide variety of infections caused by anaerobic Gram-negative bacteria, such as *Helicobacter pylori*, and for treatment of infections caused by *Clostridium difficile* [17], a Gram-positive anaerobic bacillus that causes life-threating severe diarrhoea, abdominal pain, and fever [18].

The mechanism of action **1** and other nitroimidazoles involves the conversion of this prodrug, via an anaerobic 1-electron reduction of the nitro group, to a short-lived nitro radical anion, which is unstable and decomposes to give a nitrite anion and an imidazole radical. These toxic radical species can inhibit DNA synthesis and cause DNA strand breaks leading to cell death [16,19,20]. Unfortunately, metronidazole resistance has been observed in many pathogenic Gram-negative anaerobic bacteria, e.g., carbapenem resistant *P. aeruginosa*, *K. pneumoniae*, carbapenem-resistant *A. baumannii*, *Escherichia coli*, 3rd generation cephalosporin-resistant, and fluoroquinolone-resistant *Neisseria gonorrhoeae*, *Chlamydia trachomatis*, and *Yersinia pestis* [21,22]. In fact, Gram-negative bacteria are generally more resistant to multiple antibiotics than Gram-positive bacteria because their outer membrane comprises a complex lipopolysaccharide whose lipid moiety acts as an endotoxin [23]. Therefore, in order to overcome this serious problem, in the last two decades much attention has been paid to the design and synthesis of nitroimidazole hybrids and conjugates having antibacterial activity higher than that of compound **1** and possibly not involving bacterial resistance. 

**Scheme 1:**
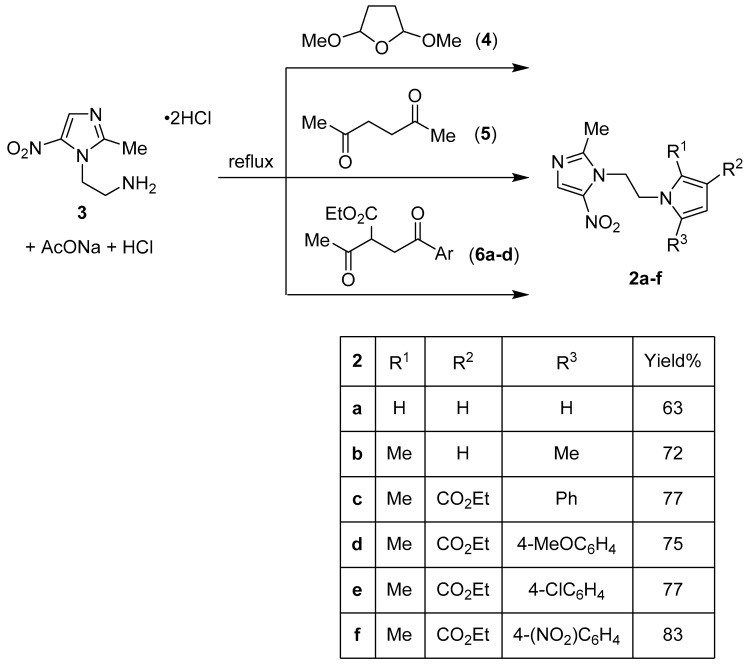
Synthesis of 5-nitroimidazole/pyrrole hybrids **2a**–**f**.

In 1999, Demirayak and coworkers [24] synthesised six 5-nitroimidazole/pyrrole hybrids of general formula **2** in yields ranging from 63 to 83% by reaction of 2-(2-methyl-5-nitro-1*H*-imidazol-1-yl)ethan-1-amine dihydrochloride (**3**) with equimolar amounts of the appropriate 1,4-diones, i.e., compounds **4**, **5**, and **6a**–**d**, and 2 eq of AcONa in AcOH under reflux for 0.5 h, under the conventional Paal-Knorr pyrrole synthesis conditions [25] (Scheme 1).

From the evaluation of the antibacterial activity of hybrids **2** against *E. coli* and multi-drug resistant *S. aureus*, it turned out that compounds **2a**–**d** possessed MIC values of 20–40 µM against *S. aureus*, while all hybrids **2a**–**f** had MIC values of 40–70 µM against *E. coli*. However, the activity of all hybrids **2** was significantly lower than that of ceftriaxone (4 and 0.1 µM against *S. aureus* and *E. coli*, respectively), a cephalosporin that was used as a reference compound [24].

**Figure 2:**
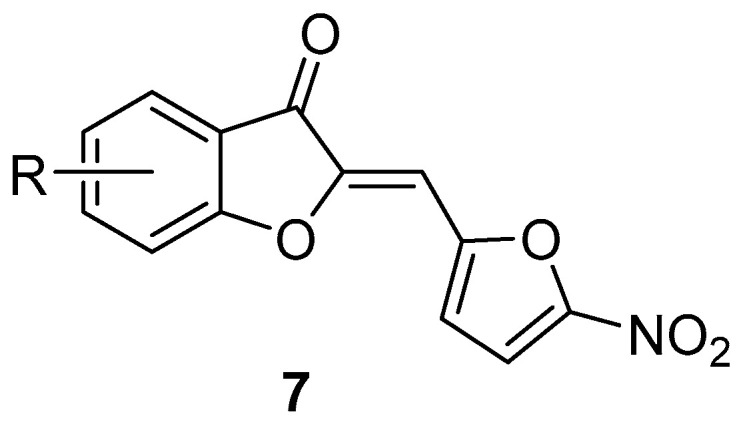
Structure of (*Z*)-2-[(5-nitrofuran-2-yl)methylene]benzofuran-3(2*H*)-ones **7**.

In 2007, Shafiee and coworkers [26], considering that (*Z*)-2-[(5-nitrofuran-2-yl)methylene] benzofuran-3(2*H*)-ones **7** (Figure 2) exhibits very good in vitro antibacterial properties against Gram-positive (*S. aureus* ATCC 25923) and a Gram-negative bacterium (*Caulobacter crescentus* NA 1000) [27], synthesised (*Z*)-2-[(1-methyl-5-nitro-1*H*-imidazol-2-yl)methylene]benzofuran-3(2*H*)-ones **8** and (*Z*)-2-[(1-methyl-4-nitro-1*H*-imidazol-5-yl)methylene] benzofuran-3(2*H*)-ones **9** and tested the in vitro antibacterial properties of these nitroimidazole hybrids against the Gram-positive bacteria *S. aureus*, methicillin-resistant *S. aureus* (MRSA), *Staphylococcus epidermidis*, and *Bacillus subtilis*, and the Gram-negative bacterium *K. pneumoniae.*

**Scheme 2:**
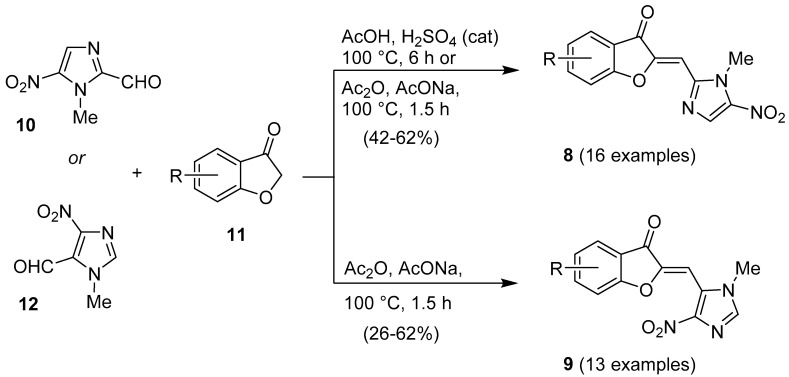
Synthesis of hybrids **8** and **9**.

Hybrids **8** and **9** were synthesised as outlined in Scheme 2. 1-Methyl-5-nitro-1*H*-imidazole-2-carbaldehyde (**10**) was condensed with 3(2*H*)-benzofuranones **11** in AcOH at 100 °C for 6 h in the presence of a catalytic amount of sulfuric acid or by treatment with Ac_2_O in the presence of AcONa at 100 °C for 1.5 h affording compounds **8** in yields ranging from 42 to 62%.

Instead, hybrids **9** were synthesised in yields ranging from 26 to 62% by the reaction of 1-methyl-4-nitro-1*H*-imidazole-5-carbaldehyde (**12**) with benzofuran-3(2*H*)-ones **11** in Ac_2_O in the presence of AcONa at 100 °C for 1.5 h [26].

**Figure 3:**
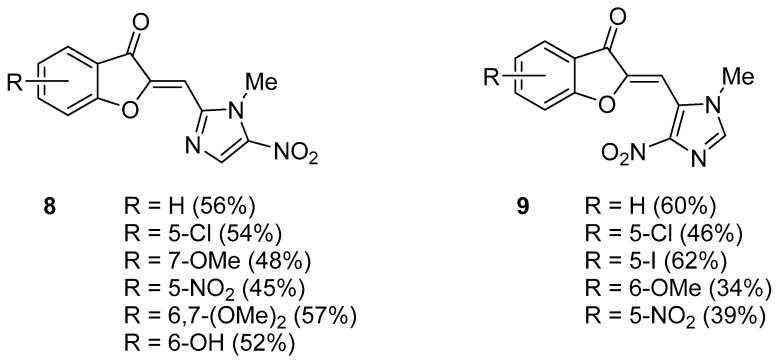
Structures and yields of some representative compounds **8** and **9** synthesised according to Scheme 2.

Figure 3 shows the chemical structures and yields of some representative compounds **8** and **9**, which were synthesised using the reactions depicted in Scheme 2.

**Scheme 3:**
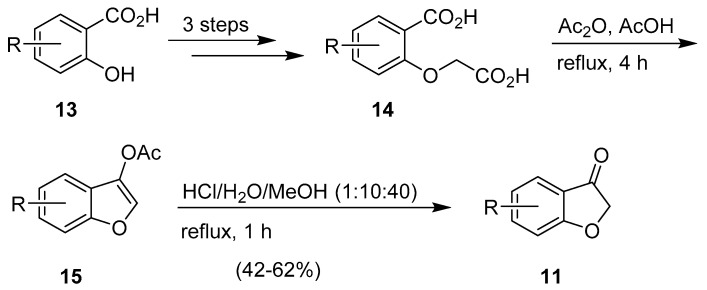
Synthesis of 3(2*H*)-benzofuranones **11.**

Compounds **11**, which were key intermediates for the synthesis of hybrids **8** and **9**, were in turn prepared starting from salicylic acids **13** via the route shown in Scheme 3 that involved the ring closure of intermediates **14** in refluxing Ac_2_O/AcOH followed by hydrolysis of the resulting compounds **15** in a mixture of HCl/H_2_O/MeOH (1:10:40) under reflux for 1 h [26].

Interestingly, most hybrids **8** showed a remarkable antibacterial activity against Gram-positive bacteria, whereas hybrids **9** exhibited no effect against selected bacteria. Compound **8m** (R = 5-NO_2_) revealed to be from 1.7 to 14 times less active than **8a** (R = H) against Gram-positive bacteria and **8n** (R = 6,7-(OMe)_2_) was found to be the most active hybrid against these bacteria [26].

In 2008, in the context of a study to find new molecules for treatment of infections caused by the microaerophilic Gram-negative bacterium clarithromycin resistant *H. pylori*, the root cause of gastric and duodenal ulcers, Foroumadi and coworkers discovered that 5-nitroimidazole/(1,3,4-thiadiazol-2-yl)-morpholine-1,1-dioxide hybrids **16** and related compounds were able to inhibit the growth of two clinical metronidazole sensitive and metronidazole resistant *H. pylori* strains [28]. For example, in a bacterial growth inhibition assay in which the disk diffusion method was used, 4-[5-(1-methyl-5-nitro-1*H*-imidazol-2-yl)-1,3,4-thiadiazol-2-yl] thiomorpholine-1,1-dioxide (**16a**) at a concentration of 8 μg/disc turned out to have inhibition zone diameters of 32 and 27 mm against these two bacterial strains, respectively.

**Scheme 4:**
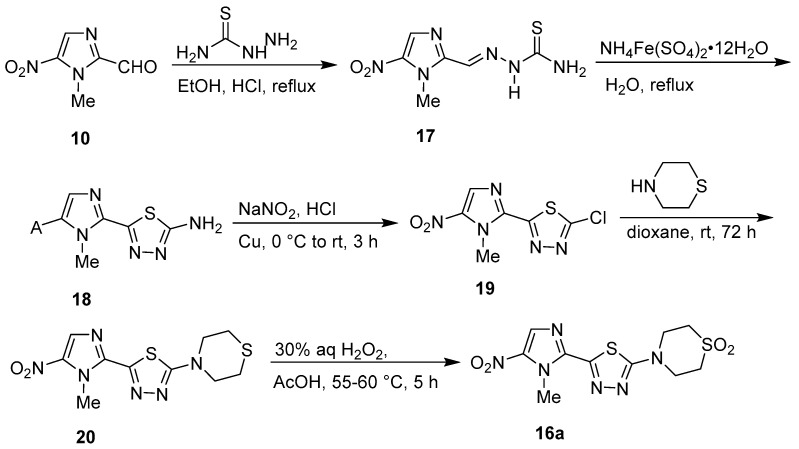
Synthesis of 4-[5-(1-methyl-5-nitro-1*H*-imidazol-2-yl)-1,3,4-thiadiazol-2-yl] thiomorpholine-1,1-dioxide (**16a**).

The synthesis of hybrid **16a** was achieved via the route depicted in Scheme 4, in which the first step was the conversion of aldehyde **10** to thiosemicarbazone **17**. Oxidative cyclisation of **17** in the presence of NH_4_Fe(SO_4_)_2_·12H_2_O [29] provided compound **18**, which by diazotation in HCl in the presence of Cu powder [30] gave 2-chloro-5-(1-methyl-5-nitro-1*H*-imidazol-2-yl)-1,3,4-thiadiazole **19**. The latter compound was treated with thiomorpholine in dioxane and the resulting compound **20** was oxidised with excess of 30% H_2_O_2_ in AcOH at 55–60 °C to give hybrid **16a** [28]. Unfortunately, the yields of this synthetic route were not reported.

**Figure 4:**
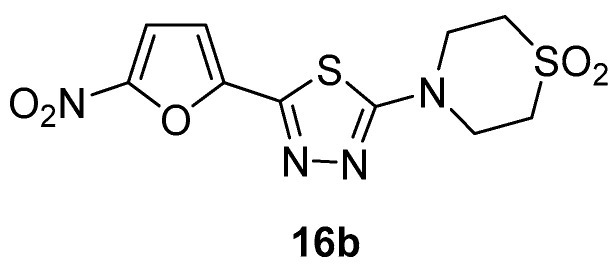
Structure of 4-[5-(5-nitrofuran-2-yl)-1,3,4-thiadiazol-2-yl]thiomorpholine-1,1-dioxide (**16b**).

However, biological data showed that 4-[5-(5-nitrofuran-2-yl)-1,3,4-thiadiazol-2-yl] thiomorpholine-1,1-dioxide (**16b**) (Figure 4), an analogue of **16a**, was more potent than this hybrid [28].

In 2009, Atia reported the synthesis of numerous hybrids containing a 2-nitroimidazole moiety starting from metronidazole (**1**) [31]. Such hybrids included three 4-arylidene-2-[1-(2-chloroethyl)-5-nitro-1*H*-imidazole-2-yl]oxazol-5(4*H*)-ones **21**, three 3′-amino-5′-arylidene-1-(2-chloroethyl)-5-nitro-3′,5′-dihydro-1*H*,4′*H*-(2,2′-biimidazol)-4′-ones **22**, nine 5′-arylidene-3′-arylideneamino-1-(2-chloroethyl)-5-nitro-3′,5′-dihydro-1*H*,4′*H*-(2,2′-biimidazol)-4′-ones **23**, three ethyl *N*-[4-arylidene-1′-(2-chloroethyl)-5′-nitro-5-oxo-4,5-dihydro-1*H*,1′*H*-(2,2′-biimidazol)-1-yl] glycinates **24**, three 2-{[4-arylidene-1′-(2-chloroethyl)-5′-nitro-5-oxo-4,5-dihydro-1*H*,1′*H*-(2,2′-biimidazol)-1-yl]amino}acetohydrazides **25**, three 2-{[4-arylidene-1′-(2-chloroethyl)-5′-nitro-5-oxo-4,5-dihydro-1*H*,1′*H*-(2,2′-biimidazol)-1-yl]glycyl}hydrazine-1-carbothioamides **26**, three 5′-arylidene-1-(2-chloroethyl)-5-nitro-3′-{[(5-sulfanyl-1,3,4-oxadiazol-2-yl)methyl]amino}-3′,5′-dihydro-1*H*,4′*H*-(2,2′-biimidazol)-4′-ones **27**, three 3′-{[(4-amino-5-sulfanyl-4*H*-1,2,4-triazol-3-yl)methyl] amino}-5′-arylidene-1-(2-chloroethyl)-5-nitro-3′,5′-dihydro-1*H*,4′*H*-(2,2′-biimidazol)-4′-ones **28**, three 5′-arylidene-1-(2-chloroethyl)-5-nitro-3′-{[(5-sulfanyl-1,3,4-thiadiazol-2-yl)methyl]amino}-3′,5′-dihydro-1*H*,4′*H*-(2,2′-biimidazol)-4′-ones **29**, and three 5′-arylidene-1-(2-chloroethyl)-5-nitro-3′-{[(5-sulfanyl-4*H*-1,2,4-triazol-3-yl)methyl]amino}-3′,5′-dihydro-1*H*,4′*H*-[2,2′-biimidazol]-4′-ones **30**. 

**Scheme 5:**
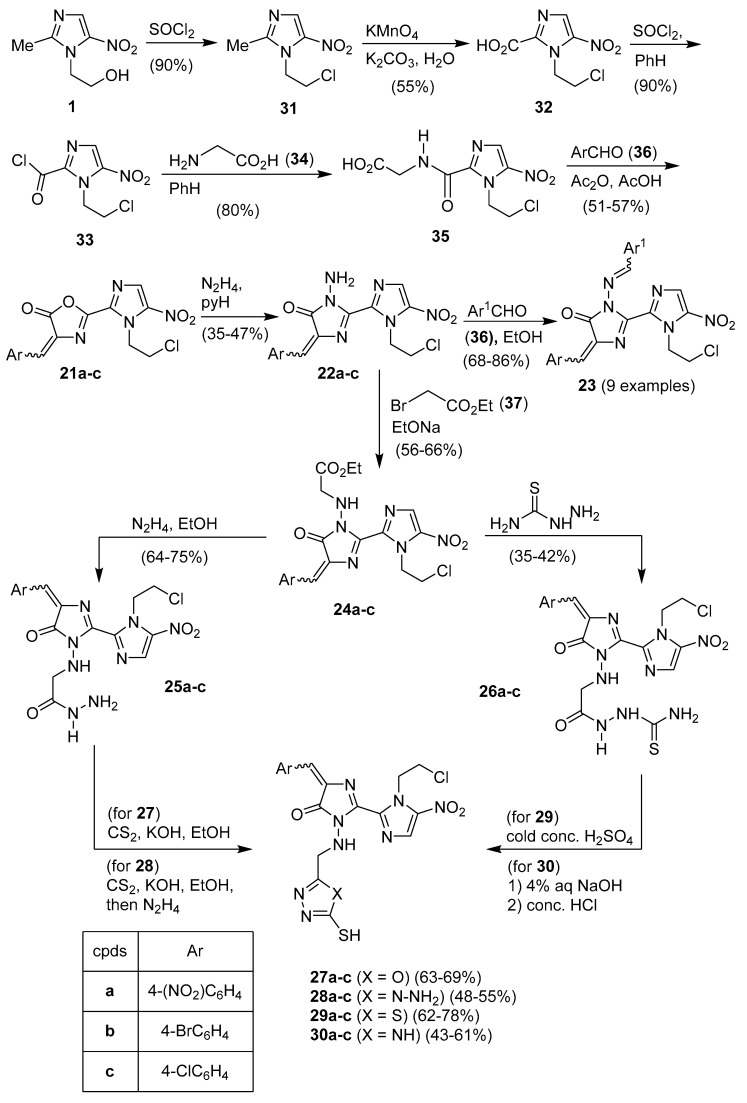
Synthesis of hybrids **21**–**30** starting from metronidazole (**1**).

The synthesis of these compounds was achieved as shown in Scheme 5. In particular, the reaction of compound **1** with SOCl_2_ provided 1-(2-chloroethyl)-2-methyl-5-nitro-1*H*-imidazole **31**, which was treated with 1 eq of KMnO_4_ and 1 eq of NaHCO_3_ in water under reflux giving rise to carboxylic acid **32** in 55% yield. The latter compound was treated with an equimolar amount of SOCl_2_ in benzene under reflux to give the 5-chloroformyl derivative **33** in 90% yield. The subsequent reaction of **33** with an equimolar amount of glycine (**34**) in the presence of a 10% aqueous solution of NaOH gave compound **35** in 80% yield. Compound **35** was then treated with an equimolar amount of aryl aldehydes **36** in a mixture of Ac_2_O and AcOH providing compounds **21** in yields ranging from 51 to 57%. The next step involved reaction with a large molar excess of 99% hydrazine hydrate in dry pyridine under reflux for 20 h gave compounds **22** in 35–47% yields, which by treatment with equimolar amounts of aryl aldehydes **36** in EtOH under reflux produced Schiff’s bases **23** in 68–86% yield. On the other hand, the reaction of compound **21** with an equimolar amount of sodium in absolute EtOH for 2 h followed by addition of an equimolar amount of ethyl bromoacetate (**37**) and heating the resulting mixture under reflux for 5 h afforded compounds **24** in 56–66% yield. Acetohydrazides **25**, which were obtained in 64–75% yield by reaction of compounds **24** with an equimolar amount of 99% hydrazine hydrate in EtOH under reflux for 8 h, were converted to compounds **27** in 63–69% yield by treatment with equimolar amounts of CS_2_ and KOH in EtOH under reflux for 3 h. Instead, the reaction of compounds **25** with an equimolar amount of CS_2_, followed by treatment of the resulting crude products with 99% hydrazine hydrate afforded hybrids **28** in yields ranging from 48 to 55%. Compounds **24** were also converted in 35–42% yield to compounds **26** by treatment with thiosemicarbazide and the reaction of the latter derivatives with 4% aqueous NaOH for 4 h, followed by acidification with conc. HCl, provided hybrids **30** in yields ranging from 43 to 61%. Instead, the reaction of compounds **26** with cold conc. sulfuric acid at room temperature for 24 h afforded hybrids **29** in yields ranging from 62 to 78% (Scheme 5). Unfortunately, both the structures in the Schemes and IUPAC names in the original paper were incorrectly referred to as derivatives of a regioisomer of **1** [31].

Hybrids **21**–**30** were tested for their antibacterial activity by the agar disc-diffusion method against *S. aureus*, *E. coli*, and *Proteus mirabilis*, a facultative anaerobic Gram-negative bacterium, at a concentration of 1 mM in DMSO and it was found that: (i) all tested compounds except **23b** (Ar = 4-nitrophenyl, Ar^1^ = 3-nitrophenyl) displayed activity against *P. mirabilis*; (ii) all tested compounds except **22a** (Ar = 4-nitrophenyl) and **23d** (Ar = 4-bromophenyl, Ar^1^ = *p*-tolyl) were active towards *E. coli*; and (iii) several hybrids including **29a** (Ar = 4-nitrophenyl) exhibited high inhibition potency against *S. aureus* [31].

**Scheme 6:**
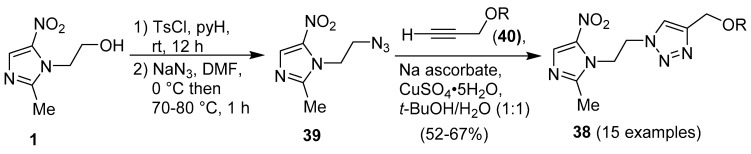
Synthesis of metronidazole/1,2,3-triazole conjugates **38.**

Still in 2009, Rawat and coworkers [32] conducted a study concerning the development of compounds capable of overcoming the phenomenon of resistance to metronidazole (**1**) [33,34] and in this context they synthesised metronidazole/1,2,3-triazole conjugates **38** and tested the antibacterial activity of these compounds against Gram-negative *E. coli* and *P. aeruginosa* and Gram-positive *S. aureus*. Compounds **38** were synthesised via the route shown in Scheme 6, in which the key intermediate, 1-(2-azidoethyl)-2-methyl-5-nitro-1*H*-imidazole (**39**), was prepared in two steps from **1**. The subsequent reaction of **39** with the appropriate 1-alkynes **40** in a 1:1 mixture of *t*-BuOH and water in the presence of sodium ascorbate and CuSO_4_ ·5H_2_O afforded hybrids **38** in yields ranging from 52 to 67%.

{1-[2-(2-Methyl-5-nitro-1*H*-imidazol-1-yl)ethyl]-1*H*-1,2,3-triazol-4-yl}methanol (**38a**) (R = H) and compound **38o** (R = *p*-ClC_6_H_4_) were found to exhibit potent activity against *E. coli* and *P. aeruginosa*. The IC_50_ values of **38a** against these bacteria were of 360 and 710 nM, respectively, and the MIC values of **38o** were 8 and 55 nM, respectively. Tetracycline, which was used as a reference drug, possessed IC_50_ values of 200 and 140 nM, respectively, against these two bacterial strains. Interestingly, compound **38a** also exhibited antibacterial activity against *S. aureus* and *S. epidermidis* with IC_50_ values of 1.4 μM. Potent activity against *E. coli* and *P. aeruginosa* (IC_50_ = 200 nM) was also displayed by compound **38l** (R = 4-(CHO)C_6_H_4_), but it was inactive against Gram-positive bacteria. Compounds **38b** (R = Me), **38c** (R = Ph), **38d** (R = 2-MeC_6_H_4_), **38i** (R = 2-(NO_2_)C_6_H_4_), **38j** (R = 4-(NO_2_)C_6_H_4_), **38f** (R = 2-MeC_6_H_4_), **38h** (R = 3-MeC_6_H_4_), **38k** (R = 2-(CHO)C_6_H_4_), **38m** (R = 4-(MeCO)C_6_H_4_), and **38n** (R = 2-ClC_6_H_4_) had no activity towards the tested Gram-positive and Gram-negative bacteria [32].

**Scheme 7:**
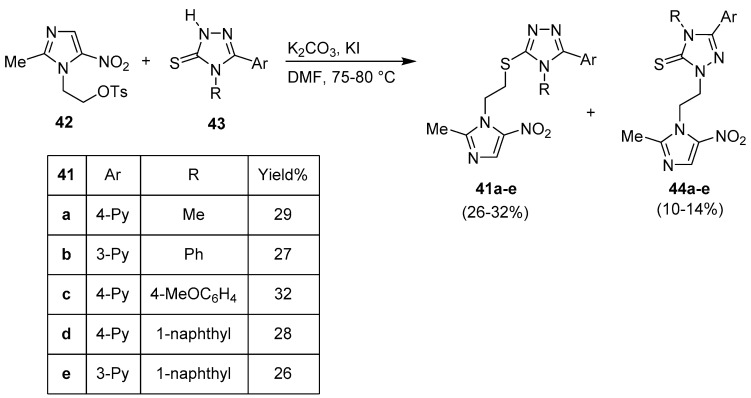
Synthesis of 5-nitroimidazole/3-sulfanyl-1,2,4-triazole hybrids **41a**–**e** and 5-nitroimidazole/1,2,4-triazole-3-thione hybrids **44a**–**e.**

In 2010, Saadeh, Mubarak, and coworkers [35] investigated the antimicrobial activity of some 5-nitroimidazole/3-sulfanyl-1,2,4-triazole hybrids of general formula **41**, which were synthesised via the route shown in Scheme 7 starting from metronidazole tosylate (**42**) [36] and 1,2,4-triazole-3-thiones **43** [37].

Specifically, compound **42** was treated with compounds **43** in DMF at 75–80 °C in the presence of K_2_CO_3_ and KI affording a mixture of 5-nitroimidazole/3-sulfanyl-1,2,4-triazole hybrids **41** and 5-nitroimidazole/1,2,4-triazole-3-thione hybrids **44** in 26–32% and 10–14% yield, respectively. These compounds, which were separated by flash chromatography, were tested for their antibacterial activity against bacterial species including *P. aeruginosa*, *E. coli*, *S. aureus*, and *Clostridium sporogenes*, a Gram-positive anaerobic bacterium, and it was found that many of them had not detectable activity at concentration as high as 0.5 mg/cm^3^. Nevertheless, hybrids **41c** and **41e** turned out to exhibit antibacterial activity against *C. sporogenes* with MIC values of 18 ± 6 and 17 ± 6 µM, respectively, lower than that of metronidazole (**1**) (43 ± 16 µM), which was used as the reference compound [35].

**Scheme 8:**
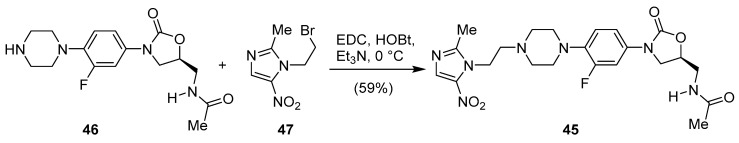
Synthesis of (*S*)-*N*-{[3-(3-fluoro-4-{4-[2-(2-methyl-5-nitro-1*H*-imidazol-1-yl)ethyl]-piperazin-1-yl}phenyl)-2-oxooxazolidin-5-yl]methyl}acetamide (**45**).

Still in 2010, Sahu and coworkers reported that (*S*)-*N*-{[3-(3-fluoro-4-{4-[2-(2-methyl-5-nitro-1*H*-imidazol-1-yl)ethyl]piperazin-1-yl}phenyl)-2-oxooxazolidin-5-yl]methyl}acetamide (**45**), a 5-nitroimidazole/oxazolidinone hybrid, was a potent antibacterial compound against *Bacillus cereus* MTCC 430, a facultative anaerobic Gram-positive bacterium, with an MIC value of 200 nM [38]. Compound **45** was synthesised in 59% yield by reaction of the piperazine derivative **46** with 1-bromomethyl-2-methyl-5-nitro-1*H*-imidazole (**47**) in Et_3_N at 0 °C in the presence of hydroxybenzotriazole (HOBt) and (3-dimethylaminopropyl)-1-ethylcarbodiimide (EDC) (Scheme 8) [38].

**Scheme 9:**
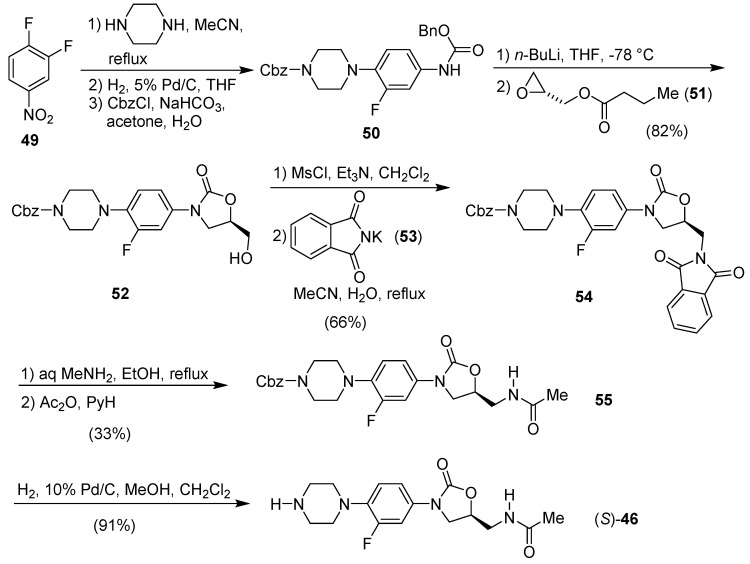
Synthesis of (*S*)-*N*-({3-[3-fluoro-4-(piperazin-1-yl)phenyl]-2-oxooxazolidin-5-yl} methyl)acetamide hydrochloride (*S*)-(**46**).

In 2011, Foroumadi and coworkers [39] synthesised hybrid (*S*)-*N*-{[3-[3-fluoro-4-[4-[5-(1-methyl-5-nitro-1*H*-imidazol-2-yl)-1,3,4-thiadiazol-2-yl]-1-piperazinyl]phenyl-2-oxooxazolidin-5-yl]methyl}acetamide (**48**) and described the experimental details of the preparation of the key intermediate of this hybrid, the piperazine derivative (*S*)-**46**, starting from 3,4-difluoronitrobenzene (**49**). Scheme 9 shows the synthetic procedure used for the synthesis of (*S*)-**46** from **49**. The latter compound was converted in three steps and 44% yield to benzyl 4-(4-{[(benzyloxy) carbonyl]amino}-2-fluorophenyl)piperazine-1-carboxylate (**50**). The subsequent reaction of **46** with 1.6 M *n*-BuLi–hexane in THF at −78 °C for 1.5 h followed by addition of commercially available (*R*)-glycidyl butyrate (**51**), stirring of the resulting mixture at room temperature for 3.5 h, addition of saturated aqueous NH_4_Cl, and extraction with AcOEt provided compound **52** in 82% yield. 

**Scheme 10:**
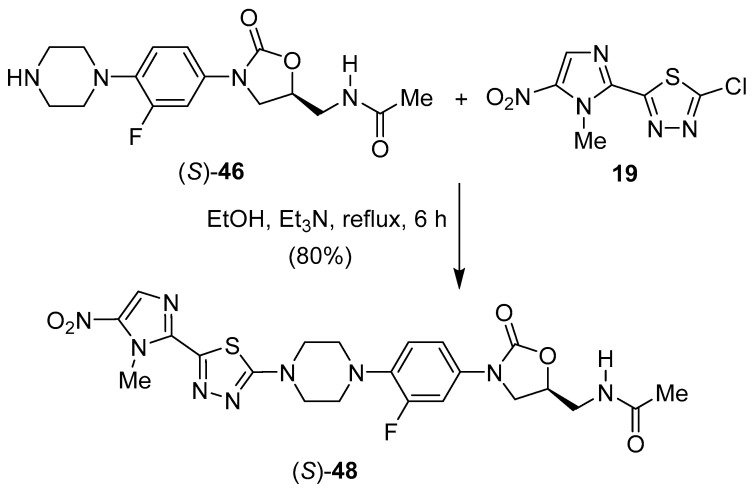
Synthesis of (*S*)-*N*-{[3-[3-fluoro-4-[4-[5-(1-methyl-5-nitro-1*H*-imidazol-2-yl)-1,3,4-thiadiazol-2-yl]-1-piperazinyl]phenyl-2-oxooxazolidin-5-yl]methyl}acetamide (*S*)-(**48**) from (*S*)-**46** and chlorothiadiazole **19.**

Such intermediate was then treated with methanesulfonyl chloride in CH_2_Cl_2_ in the presence of Et_3_N at 0 °C for 1.5 h and at room temperature for 3 h and then with potassium phthalimide (**53**) in MeCN under reflux for 48 h affording phthalimide **54** in 66% yield. Deprotection of the latter compound by treatment with 40% MeNH_2_ in water and EtOH under reflux for 6.5 h followed by reaction with Ac_2_O and pyridine provided benzyl (*S*)-4-{4-[5-(acetamidomethyl)-2-oxooxazolidin-3-yl]-2-fluorophenyl}piperazine-1-carboxylate (**55**) in 33% yield. The next step involved the reaction of **55** with a catalytic amount of 10% Pd on carbon in MeOH and CH_2_Cl_2_ under hydrogen atmosphere for 48 h, which gave rise to compound (*S*)-**46** in 91% yield (Scheme 9) [39]. Finally, compound (*S*)-**46** was converted to target (*S*)-**48** in 80% yield by treatment with chlorothiadiazole **19** in EtOH in the presence of Et_3_N (Scheme 10) [39].

Foroumadi and coworkers also tested the antibacterial activity of hybrid (*S*)-**48** and discovered that this compound displayed potent activity at non-cytotoxic concentrations that was higher than that of the reference drugs against the tested Gram-positive bacteria, i.e., *B. subtilis* PTCC 1023, *Corynebacterium glutamicum* ATCC 13032, *Micrococcus luteus* ATCC 9341, MRSA 3, MRSA 5, MRSA 17, *Staphylococcus aureus* ATCC 6538p, *S. lentus* ATCC 29070, *S. saprophyticus* ATCC 15305, *S. warneri* ATCC 27836, and *S. xylosus* ATCC 29971 [39]. For example, (*S*)-**48** had MIC values of 180 nM against *B. subtilis* PTCC 1023 and against *S. aureus* ATCC 6538p, of 44 nM against MRSA 17 and *S. saprophyticus* ATCC 15305, and of 11 nM against *S. warneri* ATCC 27836 [39].

**Scheme 11:**
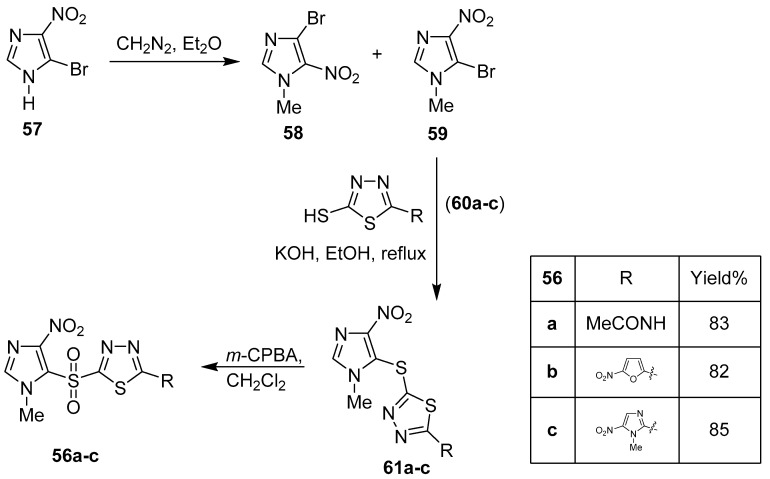
Synthesis of 2-[(1-methyl-4-nitro-1*H*-imidazol-5-yl)sulfonyl]-1,3,4-thiadiazoles **56a**–**c**.

Still in 2011, Foroumadi and coworkers reported the synthesis and in vitro antibacterial activity of three new 2-[(1-methyl-4-nitro-1*H*-imidazol-5-yl)sulfonyl]-1,3,4-thiadiazoles **56** [40]. These hybrids were prepared via the route outlined in Scheme 11, in which 4(5)-bromo-5(4)-nitro-1*H*-imidazole (**57**) [41] was treated with diazomethane in Et_2_O affording a mixture of 4-bromo-1-methyl-5-nitro-1*H*-imidazole (**58**) and 5-bromo-1-methyl-4-nitro-1*H*-imidazole (**59**), in which the latter compound was the major component. Treatment of **59** with the appropriate 1,3,4-thiadiazole-2-thiols **60** in EtOH under reflux in the presence of KOH afforded compounds **61**. Finally, a mixture of **61** and 3 eq of *m*-chloroperbenzoic acid and 3 eq of NaHCO_3_ in CH_2_Cl_2_ was stirred at room temperature for three days providing the required hybrids **56a**–**c** in 82–85% yield.

It was then found that 2-[(1-methyl-4-nitro-1*H*-imidazol-5-yl)sulfonyl]-5-(5-nitrofuran-2-yl)-1,3,4-thiadiazole (**56b**) and 2-[(1-methyl-4-nitro-1*H*-imidazol-5-yl)sulfonyl]-5-(1-methyl-5-nitro-1*H*-imidazol-2-yl)-1,3,4-thiadiazole (**56c**) possessed antibacterial activity against *K. pneumoniae* with MIC values of 41 and 80 μM, respectively, and *E. coli* with an MIC value of 41 and 40 μM, respectively. However, compounds **56b** and **56c** turned out to be less active than the reference drug norfloxacin (780 and 410 nM against *K. pneumoniae* and *E. coli*, respectively). Furthermore, *N-*{5-[(1-methyl-4-nitro-1*H*-imidazol-5-yl)sulfonyl]-1,3,4-thiadiazol-2-yl}acetamide (**56a**) showed no activity against Gram-positive and Gram-negative bacteria [40].

**Scheme 12:**
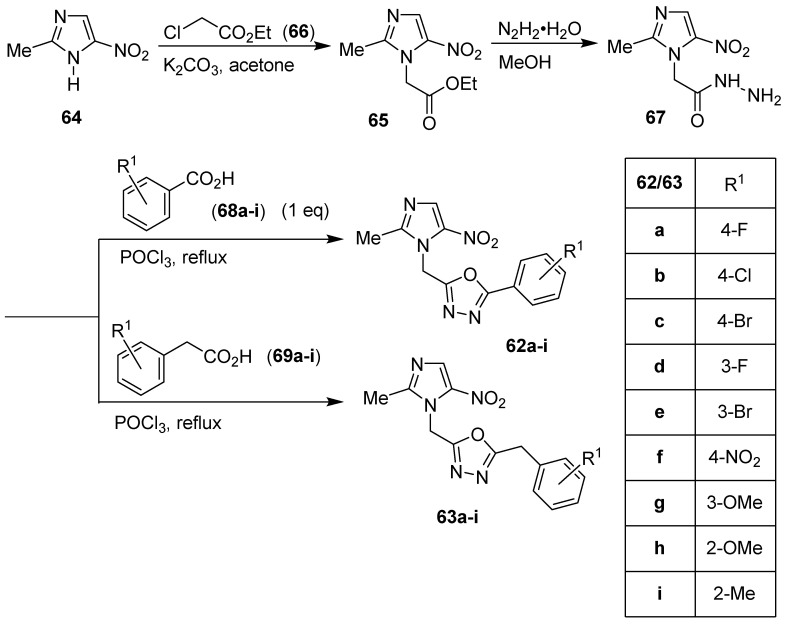
Synthesis of 5-nitroimidazole/1,3,4-oxadiazole hybrids **62a**–**i** and **63a**–**i.**

In 2012, two novel series of 5-nitroimidazole/1,3,4-oxadiazole hybrids of general formula **62** and **63** were synthesised from Gong, Zhu, and coworkers starting from commercially available 2-methyl-5-nitro-1*H*-imidazole **64** using the protocol depicted in Scheme 12 [42]. Compound **65**, which was obtained by reaction of **64** with ethyl chloroacetate (**66**) in acetone in the presence of K_2_CO_3_, was converted to 2-(2-methyl-5-nitro-1*H*-imidazol-1-yl)acetohydrazide (**67**) by hydrazinolysis in MeOH. Subsequent treatment of **67** with an equimolar amount of benzoic acids **68a**–**i** in phosphoryl chloride under reflux for 10–16 h provided hybrids **62a**–**i**. Instead, the reaction of **67** with phenylacetic acids **69** in refluxing POCl_3_ gave hybrids **63**. Unfortunately, the yields of hybrids **62** and **63** were not reported [42]. 

All these hybrids were then screened for their antibacterial activities against two Gram-negative bacterial strains, *E. coli* and *P. aeruginosa*, and two Gram-positive bacterial strains, *B. subtilis* and *S. aureus*, by the MTT assay [43]. Hybrids **62e**, **62h**, and **62i** were found to possess significant antibacterial activities with MIC values of 4.9–17 µM against *E. coli* ATCC 35128, but hybrids **63e**, **63h**, and **63i** displayed less potent activity with MIC values of 42–160 µM. Compounds **62h** and **62i** turned out to be the most potent among the tested hybrids [42].

**Figure 5:**
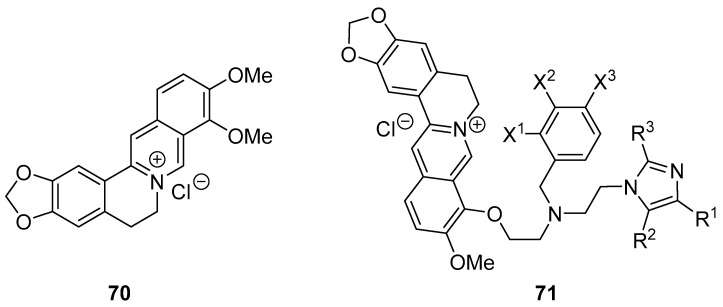
Structures of berberine (**70**) and hybrids **71.**

In 2013, Geng, Zhou, and coworkers [44] synthesised, characterised, and evaluated the antimicrobial activity of a series of hybrids of nitroimidazoles and berberine (**70**) (Figure 5), the major isoquinoline alkaloid isolated from a number of medicinal plants including *Coptis chinensis* Franch. and *Berberis* spp. [45] that has been widely used as a drug for treatment of many diseases and exhibits antibacterial activity against MRSA [46], *E. coli* [47], *S. epidermidis* [48], *Vibrio vulnificus* [49], *Streptococcus agalactiae* [47], *Acromonas hydrophila* [47], and *Edwardsiella ictaluri* [47].

**Scheme 13:**
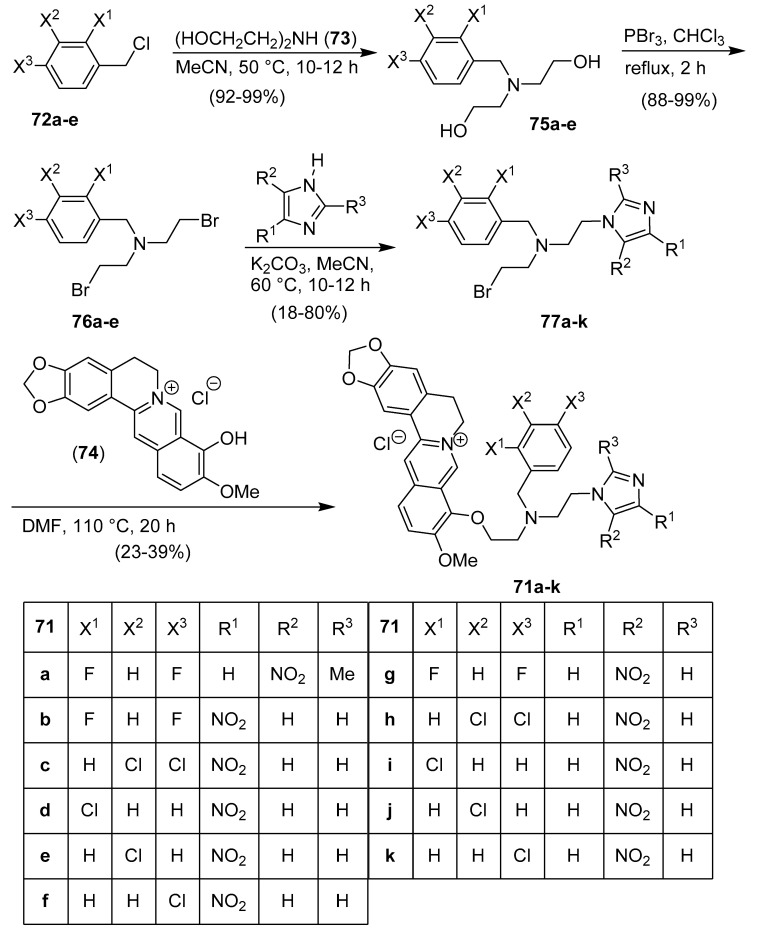
Synthesis of nitroimidazole/berberine hybrids **71a**–**k.**

As shown in Scheme 13, the target berberine/nitroimidazole hybrids **71a**–**k** (Figure 5) were prepared from commercially available halobenzyl chlorides **72a**–**e**, diethanolamine (**73**), and berberrubine (**74**), one of the major metabolites of the naturally occurring alkaloid berberine with appreciable anti-ulcerative colitis effect [47].

*N*-Benzylation of diethanolamine with compounds **72** produced intermediates **75**, which were treated with PBr_3_ in CHCl_3_ to afford dibromides **76**. The subsequent reaction between 1 eq of compounds **76** and 1 eq of 4-nitro-1*H*-imidazole or 2-methyl-5-nitro-1*H*-imidazole in MeCN at 60 °C in the presence of 2 eq of K_2_CO_3_ provided compounds **77a**–**k** in modest to good yields. Finally, target hybrids **71a**–**k** were obtained in 23–39% yields by treatment of compounds **77** with 0.98 eq of berberrubine (**74**) at 110 °C in DMF for 20 h. Compound **74** was in turn obtained in 88% yield by demethylation of berberine (**70**) at 190 °C under reduced pressure for 15 min [44].

Notably, most of the target hybrids turned out to exhibit effective antibacterial activity against Gram-positive and Gram-negative bacterial strains and their potency against MRSA was superior or comparable to that of reference drugs norfloxacin, chloramphenicol [50], and berberine. In particular, hybrid **71g** had low inhibitory concentration towards the Gram-negative bacteria *Shigella dysenteriae* and *Proteus vulgaris* ATCC 6896 with MIC values of 6 µM for both bacteria. Instead, the MIC values for the reference drug chloramphenicol were 100 µM towards both bacterium species, while the MIC values for norfloxacin towards *S. dysenteriae* and *P. vulgaris* were 13 and 25 µM, respectively. It is also worth noting that hybrids bearing 4-nitroimidazole moiety, such as **71b** and **71f** displayed antimicrobial activities lower than those of hybrids bearing 5-nitroimidazole moiety such as **71g** [44].

**Scheme 14:**
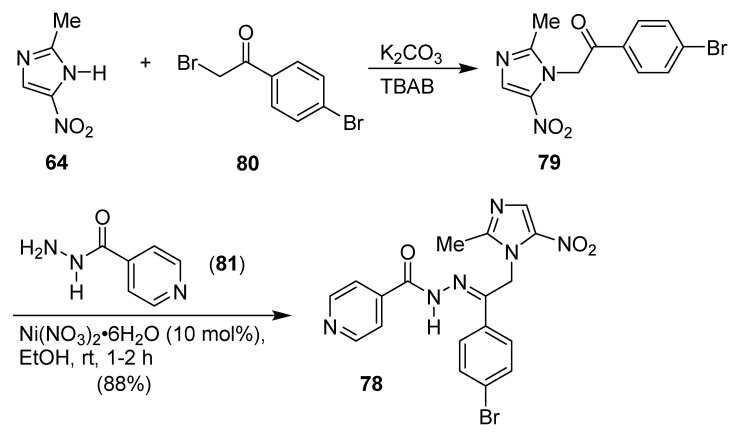
Synthesis of hybrid **78.**

Still in 2013, Zhu and coworkers reported that the Schiff’s base derivative **78** bearing a 5-nitroimidazole moiety exhibited effective inhibitory activity towards *E. coli* ATCC 35218 (MIC = 7.1 μM) that was similar to that of both standards used, kanamycin B and penicillin G (6.5 and 9.4 μM, respectively) [51]. (*E*)-*N*’-[1-(4-Bromophenyl)-2-(2-methyl-5-nitro-1*H*-imidazol-1-yl)ethylidene] isonicotinohydrazide (**78**) was synthesised via a two-step protocol in which the first step involved the preparation of the key intermediate, 1-(4-bromophenyl)-2-(2-methyl-5-nitro-1*H*-imidazol-1-yl)ethan-1-one (**79**) by treatment of 2-methyl-5-nitro-1*H*-imidazole (**64**) with 2-bromo-1-(4-bromophenyl)ethan-1-one (**80**) in MeCN in the presence of K_2_CO_3_ and phase transfer catalyst tetra-*n*-butylammonium bromide (TBAB), and the second step involved the Ni(NO_3_)_2_·6H_2_O-catalysed reaction of **79** with hydrazide **81** in EtOH at room temperature (Scheme 14) [51].

**Scheme 15:**
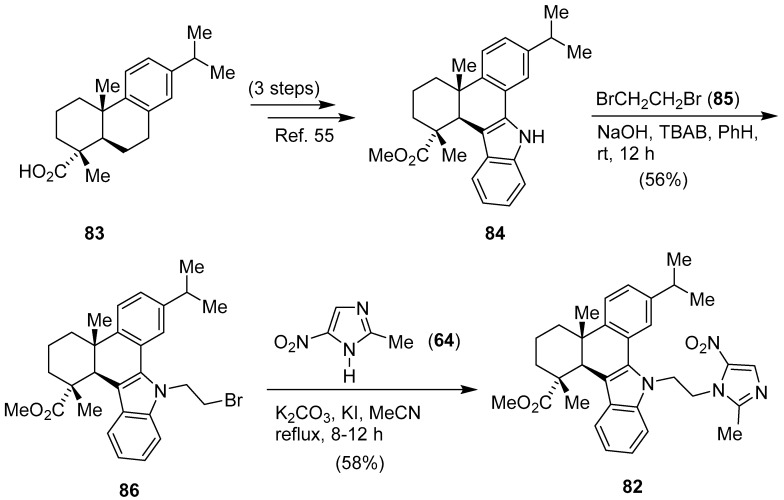
Synthesis of methyl (1*R*,4*aS*)-7-isopropyl-1,4*a*-dimethyl-9-[2-(2-methyl-5-nitro-1*H*-imidazol-1-yl)ethyl]-2,3,4,4*a*,9,13c-hexahydro-1*H*-dibenzo[*a*,*c*]carbazole-1-carboxylate (**82**).

In 2014, Gu and coworkers synthesised methyl (1*R*,4*aS*)-7-isopropyl-1,4*a*-dimethyl-9-[2-(2-methyl-5-nitro-1*H*-imidazol-1-yl)ethyl]-2,3,4,4*a*,9,13c-hexahydro-1*H*-dibenzo[*a*,*c*]carbazole-1-carboxylate (**82**), a novel hybrid of 2-methyl-5-nitro-1*H*-imidazole (**64**) and a 1*H*-dibenzo [*a*,*c*]carbazole derivative, from dehydroabietic acid (**83**) (Scheme 15) and evaluated the antimicrobial activity of this hybrid against Gram-positive bacteria *B. subtilis* CGMCC 1.1162 and *S. aureus* CGMCC 1.1361 and Gram-negative bacteria *E. coli* CGMCC 1.1571 and *Pseudomonas fluorescens* CGMCC 1.182086 [52]. The developed synthetic route involved the conversion of naturally occurring **83**, a compound of which many derivatives have been shown to possess interesting biological activities [53,54], to intermediate **84** as described in the literature [55]. *N*-Alkylation of the latter compound by treatment with a large molar excess of 1,2-dibromoethane (**85**) in the presence of NaOH and TBAB afforded the *N*-bromoethyl carbazole derivative **86** in 56% yield. Finally, the target hybrid **82** was obtained in 58% yield by reaction of **86** with 2 eq of nitroimidazole **64** in MeCN under reflux in the presence of 5 eq of K_2_CO_3_ and 1 eq of KI. 

Gu and coworkers then found that hybrid **82** displayed potent antibacterial activity against *B. subtilis* CGMCC 1.1162 with an MIC value 1.6 μM that was comparable to that of the reference antibiotic amikacin (1.5 μM). Hybrid **82** also turned out to possess significant antibacterial activity against *S. aureus* CGMCC 1.1361, but low activity against *E. coli* CGMCC 1.1571 and *P. fluorescens* CGMCC 1.182086 [52].

**Scheme 16:**
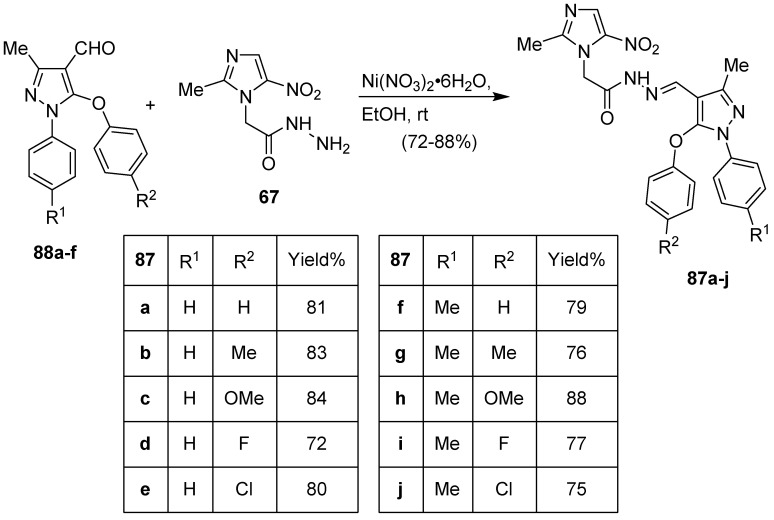
Synthesis of hybrids **87a**–**j.**

In 2015, Sangani, Zhu, and coworkers [56] synthesised Schiff’s base derivatives **87a**–**j** bearing 5-nitroimidazole and pyrazole moieties by Ni(NO_3_)_2_·6H_2_O-catalysed reaction of 5-aryloxypyrazole-4-carbaldehydes **88a**–**j** [57] with 2-(2-methyl-5-nitro-1*H*-imidazol-1-yl) acetohydrazide (**67**) [58] in EtOH at room temperature. As shown in Scheme 16, this reaction provided hybrids **87a**–**j** in 75–88% yield. 

These compounds were tested for their antibacterial properties against *E. coli* ATCC 25922, *P. aeruginosa* ATCC 27853, *B. subtilis* ATCC 530, and *S. aureus* ATCC 25923 and for inhibition of *E. coli* FabH activity, and many of these hybrids proved to be effective against the applied bacterial strains. Compounds **87h** (MIC = 6.2 μM) and **87i** (MIC = 6.4 μM) showed higher activity against *E. coli* than other hybrids and their activities were comparable to that of antibiotic kanamycin B (MIC = 6.5 μM). Furthermore, **87i** turned out to show the most effective inhibition (IC_50_ = 4.6 ± 0.2 μM) by binding into the active site of *E. coli* FabH receptor with minimum binding energy [56].

**Scheme 17:**
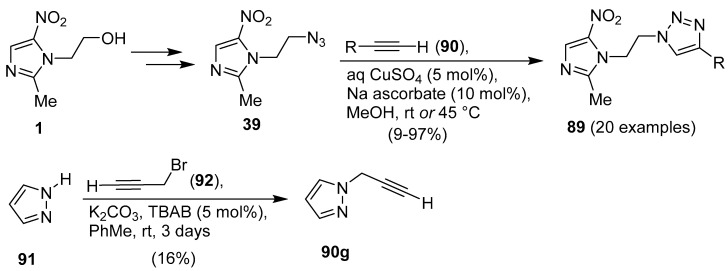
Synthesis of metronidazole/triazole conjugates **89** and 1-(prop-2-yn-1-yl)-1*H*-pyrazole (**90g**).

Still in 2015, Cooper and coworkers [59], taking into account that resistance to metronidazole (**1**) had been observed in Gram-positive and Gram-negative anaerobic bacteria [21,56], reasoned that suitable metronidazole derivatives consisting of metronidazole/triazole conjugates (Mtz-triazoles) might exhibit potent activity against anaerobic bacteria for which compound **1** is used as a treatment. Thus, they synthesised metronidazole/triazole conjugates **89** via the route shown in Scheme 17.

Specifically, azide **39**, which was prepared in high yield from mesylate prepared from **1**, was submitted to a CuSO_4_·5H_2_O/sodium ascorbate-catalysed 1,3-dipolar cycloaddition to a library of 1-alkynes **90** in MeOH at 45 °C, affording Mtz-triazoles **89** in yields ranging from 9 to 97%. All 1-alkynes were commercially available with the exception of **90g**, which was prepared in 16% yield by reaction of pyrazole (**91**) with propargyl bromide (**92**) and K_2_CO_3_ in toluene in the presence of TBAB (Scheme 17) [59].

Compounds **89** were then tested for their activity under aerobic conditions against *C. difficile*, microaerophilic *H. pylori*, and other microorganisms including ESKAPE pathogens (MRSA, *E. coli*, *K. pneumoniae*, *P. aeruginosa*, and *A. baumannii*). For example, the MIC values of Mtz-triazoles **89a** (R = Ph), **89b** (R = 4-MeOC_6_H_4_), **89j** (R = 2-pyridyl), **89n** (R = 3-thienyl), and **89t** (R = CH_2_NH_2_) were higher than 100 μM against the representative ESKAPE pathogens, but **89a** and **89q** (R = CH_2_OH) had MIC values higher than 210 μM against a panel of eight additional drug resistant *S. aureus* strains, vancomycin resistant *E. faecium*, and multidrug-resistant, penicillin non-susceptible Gram-positive *Streptococcus pneumoniae*. It is worth noting that this lack of activity contrasted with what was reported in 2009 by Rawat and coworkers [32], but it was consistent with the reported inactivity of 378 Mtz-triazoles against *E. coli* that was observed in the study of Eckmann and coworkers [48]. It must also be pointed out that: (i) several metronidazole/triazole conjugates **89** displayed potent activity against multiple strains of *C. difficile*, including two major pathogenic strains of NAP1/027 and a VPI 10463 strain associated with epidemics; and (ii) cross-resistance to metronidazole was observed against stable metronidazole resistant *C. difficile* strains [59].

In a study carried out in 2015, Geng, Zhou, and coworkers synthesised and characterised quinolone-based metronidazole derivatives **93a**–**l** and tested these hybrids for their antibacterial activities towards the Gram-negative bacteria *E. coli*, *Proteus hauseri* [60], *P. aeruginosa*, and *Salmonella enterica* and the Gram-positive bacteria MRSA, *S. aureus*, *B. subtilis*, and *M. luteus* [61].

**Scheme 18:**
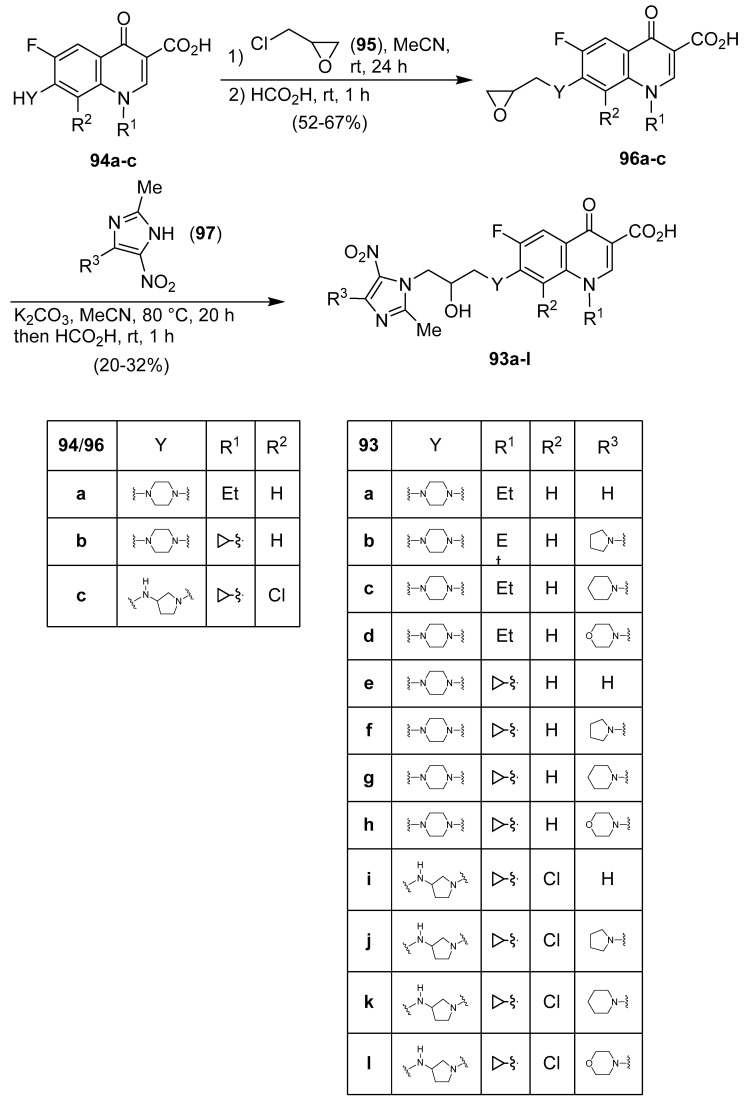
Synthesis of quinolone/imidazole hybrids **93a**–**l.**

As shown in Scheme 18, compounds **93** were synthesised by *N*-alkylation of commercially available 4-quinolone-2-carboxylic acids **94a**–**c** with 2-(chloromethyl)oxirane (**95**) in MeCN at room temperature, followed by treatment of the resulting compounds **96** with formic acid to adjust the pH value to 5.5–6.5 and by addition of an equimolar amount of 2-methyl-5-nitro-1*H*-imidazoles **97** in MeCN at 80 °C for 20 h in the presence of K_2_CO_3_. Hybrids **93a**–**l** were so obtained in yields ranging from 20 to 32% [61].

**Figure 6:**
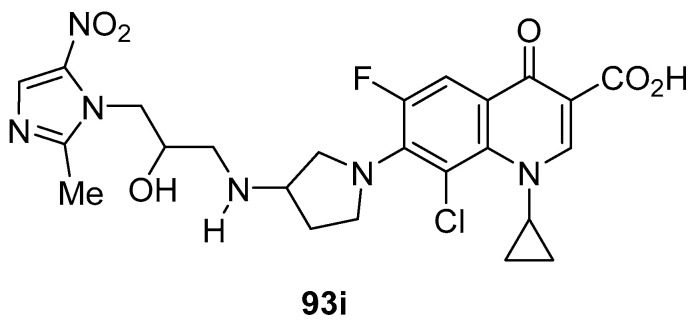
Structure of hybrid **93i.**

It is noteworthy that most of them exhibited good antibacterial activity towards the above-mentioned Gram-positive and Gram-negative bacteria, and that 8-chloro-1-cyclopropyl-6-fluoro-7-(3-{[2-hydroxy-3-(2-methyl-5-nitro-1*H*-imidazol-1-yl)propyl]amino}pyrrolidin-1-yl)-4-oxo-1,4-dihydroquinoline-3-carboxylic acid (**93i**) (Figure 6) possessed antibacterial activity against Gram-negative *P. aeruginosa* with an MIC value of 460 nM, which was lower than that of the reference drugs chloramphenicol [50] (50 μM), norfloxacin (13 μM), ciprofloxacin (3 μM), and clinafloxacin (3 μM) [61].

The interaction of hybrid **93i** with *P. aeruginosa* DNA and Cu^2+^ ion was also investigated, and it was established that **93i** could intercalate into *P. aeruginosa* DNA through Cu^2+^ ion bridge to form a steady **93i**-Cu^2+^-DNA ternary complex, which might further block DNA replication to exert the powerful bioactivities [61].

In a study carried out in 2015 by Hurdle and coworkers, the synthesis of twenty-six *N*-substituted metronidazole/tetramic acid hybrids **98** was reported, and clear evidence was provided that the localisation of metronidazole by a lactobacilli-inspired tetramic acid motif improves treatment outcomes in the hamster model of *C. difficile* infection [62]. Four leads, i.e., **98a** (R = isobutyl), **98b** (R = 4-phenylbenzyl), **98c** (R = 1-naphthylmethyl), and **98d** (R = 1-methyl-3-(1*H*)-indolylmethyl), were found to be more effective than metronidazole (**1**) in *C. difficile*-infected animals retaining the mode of action of metronidazole and demonstrated lack of propensity for de novo resistance. This study also suggested a role for the tetramic acid motif for colon-specific drug delivery. Furthermore, the structure-activity relationship of metronidazole/tetramic acid hybrids showed that substitution at the 5-position of the tetramic core was an important factor for activity, as the hybrid lacking a 5-substituent, i.e., **98e** (R = H), was, on a molar basis, more than 80 times less active than **98a** [62].

**Scheme 19:**
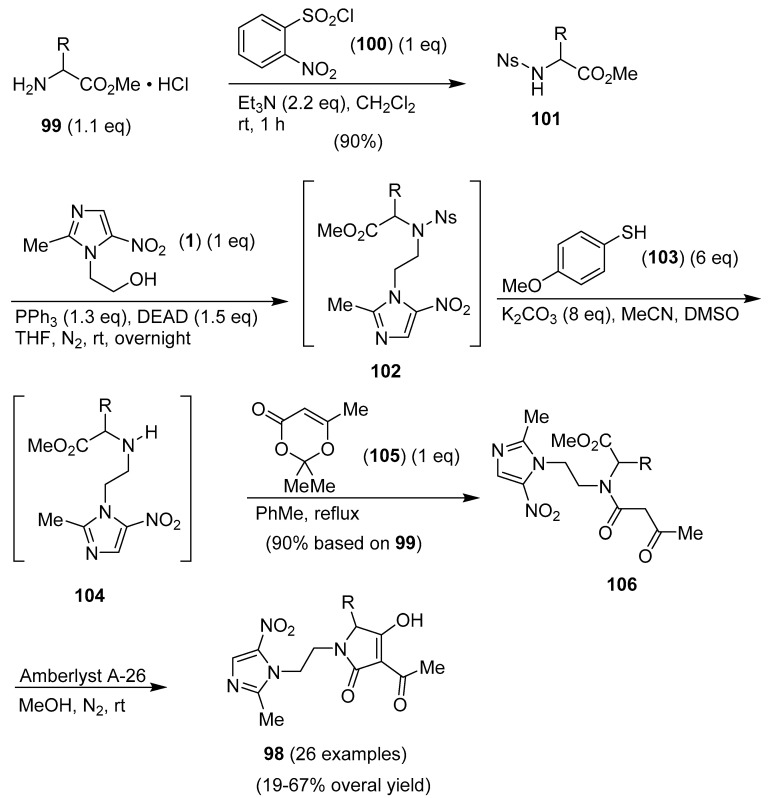
Synthesis of *N*-substituted metronidazole/tetramic acid hybrids **98.**

Compounds **98** were synthesised via the route shown in Scheme 19.

In particular, α-aminoacid methyl ester hydrochlorides **99** were treated with 2-nitrobenzenesulfonyl chloride (nosyl chloride) (**100**) in CH_2_Cl_2_ in the presence of Et_3_N, and the resulting compounds **101** treated with metronidazole (**1**), PPh_3_, and diethyl azodicarboxylate (DEAD) in THF under N_2_ atmosphere to afford compounds **102**. These crude intermediates were treated with a large molar excess of 4-methoxybenzenethiol (**103**) in CH_2_Cl_2_ in the presence of K_2_CO_3_ and the resulting crude compounds **104** treated with 2,2,6-trimethyl-4*H*-1,3-dioxin-4-one (**105**) in dry toluene under reflux for 2 h to give compounds **106**. Finally, treatment of **106** with 2 eq of Amberlyst A-26 (OH) resin in dry MeOH overnight at room temperature under N_2_ atmosphere provided the required hybrids **98** in yields ranging from 19 to 67% (Scheme 19) [62].

**Scheme 20:**
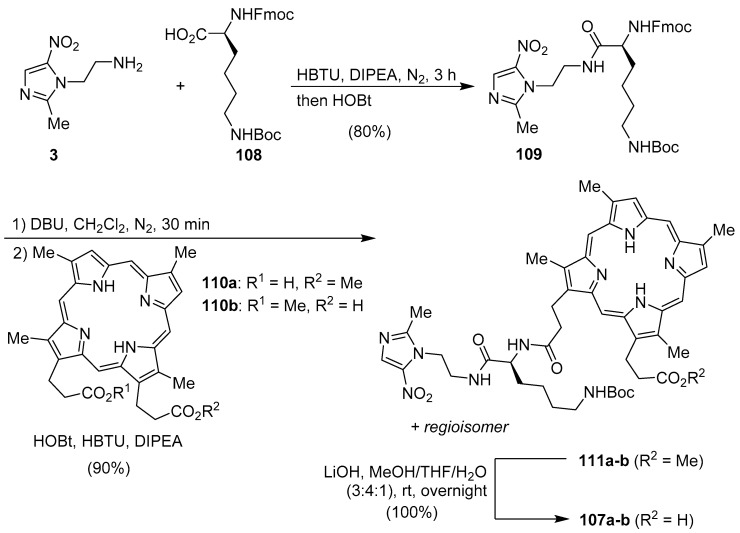
Synthesis of *L*-aminoacid-linked porphyrin-nitroimidazole adducts DPIX-Lys(Boc)-Nim adducts **107a** and **107b.**

Still in 2015, Crossley and coworkers developed a successful methodology for the synthesis of amino-acid linked porphyrin-nitroimidazole antibiotics targeting *Porphyromonas gingivalis* [63], the Gram-negative anaerobic periodontal pathogen that requires porphyrin supplementation for growth. The study carried out by these authors involved the synthesis of *L*-amino acid-linked deuterioporphyrin-nitroimidazole DPIX-Lys(Boc)-Nim adducts **107a** and **107b** via the reaction sequence shown in Scheme 20. In particular, the reaction of 1.03 eq of aminoimidazole **3** [64] with a solution of 1 eq of Fmoc-Lys(Boc)-OH **108** (1 eq) in DMF and CH_2_Cl_2_ in the presence of 3.1 eq of *N*,*N*’-diisopropylethylamine (DIPEA) and 1.03 eq of *O*-(benzotriazol-1-yl)-*N*,*N*,*N*’,*N*’-tetramethyl uronium hexafluorophosphate (HBTU) at room temperature for 3 h gave Fmoc-Lys(Boc)-Nim **109** in 80% yield. At the same time, DPIX monomethyl esters **110a** and **110b** were prepared in 49% yield by controlled hydrolysis of DPIX dimethyl ester with 4 M HCl in refluxing methanol. Subsequently, compound **109** was Fmoc deprotected by treatment with 1.2 eq of DBU and the resulting amino derivative was treated with the carboxylic group of monomethyl esters **110** affording adducts **111a** and **111b** in 90% yield. Finally, treatment of the latter regioisomeric compounds with a molar excess of LiOH in a mixture of MeOH, THF, and water overnight at room temperature provided free carboxylic acids **107a** and **107b** in quantitative yield (Scheme 20) [63].

Remarkably, DPIX-Lys(Boc)-Pro-Nim adducts **107a** and **107b** with a proline-lysine linker bridging metronidazole to deuterioporphyrin IX (DPIX 2) retarded the growth but did not kill *P. gingivalis* at 20 μM and, unlike metronidazole, they did not kill a range of other anaerobic bacteria isolated from the human gastro-intestinal tract [63].

**Scheme 21:**
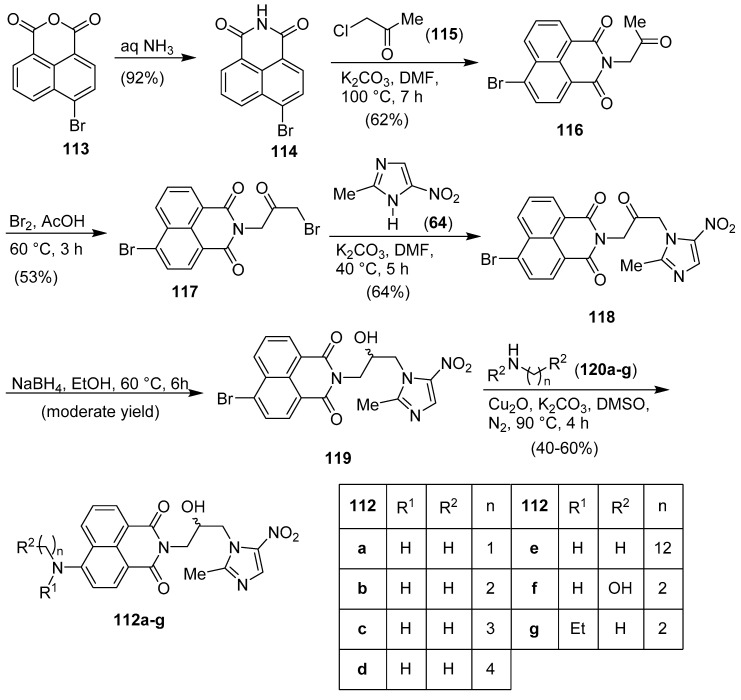
Synthesis of naphthalimide-derived metronidazoles **112a**–**g.**

In 2017, Li, Zhou, and coworkers synthesised novel naphthalimide-derived metronidazoles **112a**–**g** and tested their in vitro antibacterial activities towards Gram-positive and Gram-negative bacteria [65]. The synthesis of hybrids **112** was carried out via the reaction sequence shown in Scheme 21, in which commercially available 4-bromo-1,8-naphthalic anhydride (namely, 6-bromo-1*H*-benzo[*de*]isoquinoline-1,3(2*H*)-dione) (**113**) was the starting material.

Compound **113** was treated with aqueous ammonia producing intermediate **114** in 92% yield. The subsequent reaction of **114** with chloroacetone (**115**) in DMF in the presence of K_2_CO_3_ afforded compound **116** in 62% yield. The latter was treated with bromine in AcOH at 60 °C for 3 h providing compound **117** in 53% yield. The coupling of **117** with 2-methyl-5-nitro-1*H*-imidazole (**64**) in DMF in the presence of K_2_CO_3_ gave in 64% yield the nitroimidazole derivative **118**, which was reduced with NaBH_4_ in EtOH at 60 °C for 6 h affording compound **119** in a moderate yield. Finally, target hybrids **112a**–**g** were obtained in 40–60% yield by reaction of **119** with amines **120a**–**g** in DMSO at 90 °C for 4 h under N_2_ atmosphere using K_2_CO_3_ as base and Cu_2_O as catalyst [65].

Among these hybrids, compound **112b** (R^1^ = R^2^ = H; n = 2) proved capable of not only exhibiting effective inhibition towards the growth of both *P. vulgaris* (MIC = 2 μM), a Gram-negative bacterium that inhabits the intestinal tract of humans and animals, and *S. dysenteriae* (MIC = 10 μM), a Gram-negative fluoroquinolone-resistant bacterium that can cause shigellosis (bacillary dysentery), but also of rapidly killing the tested strains and to prevent development of bacterial resistance. Interestingly, hybrid **112b** also proved capable of intercalating into calf thymus DNA to form a steady supramolecular complex, which might block DNA replication to display the antibacterial activity and being effectively transported by human serum albumin [65].

**Scheme 22:**
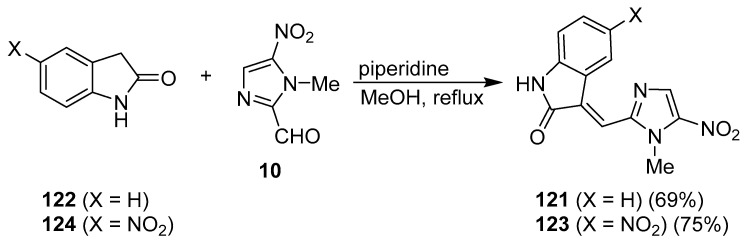
Synthesis of (*E*)-3-[(1-methyl-5-nitro-1*H*-imidazol-2-yl)methylene]indolin-2-one (**121**) and (*E*)-3-[(1-methyl-5-nitro-1*H*-imidazol-2-yl)methylene]-5-nitroindolin-2-one (**123**).

In 2018, Yang, Luo, and coworkers [66], considering that indolin-2-ones are known to possess antibacterial activity [67,68], synthesised (*E*)-3-[(1-methyl-5-nitro-1*H*-imidazol-2-yl)methylene] indolin-2-one (**121**), a hybrid of indolin-2-one and 1-methyl-5-nitro-1*H*-imidazole. Such a compound was obtained in 69% yield by reaction of indolin-2-one (**122**) with 1.2 eq of 1-methyl-5-nitro-1*H*-imidazole-2-carbaldehyde (**10**) and 1.5 eq of piperidine in MeOH under reflux (Scheme 22).

Hybrid **121** was found to exhibit antibacterial activity against *S. aureus* strains with MIC values of 7 μM and 4 μM on methicillin-sensitive *S. aureus* ATCC 25923 and MRSA ATCC 33591, respectively [66].

The protocol employed for the synthesis of **121** was then used for the synthesis of (*E*)-3-[(1-methyl-5-nitro-1*H*-imidazol-2-yl)methylene]-5-nitroindolin-2-one (**123**) in 75% yield from **10** and 5-nitroindolin-2-one (**124**) (Scheme 22). Hybrid **123** was found to possess remarkable antibacterial activity against *E. coli* ATCC 25922, *P. aeruginosa* ATCC 27853, and vancomycin-resistant *Enterococcus* strain B148, and its efficacy against ATCC 25923 was, on a molar basis, 18 times higher than that of **121** [66].

**Scheme 23:**
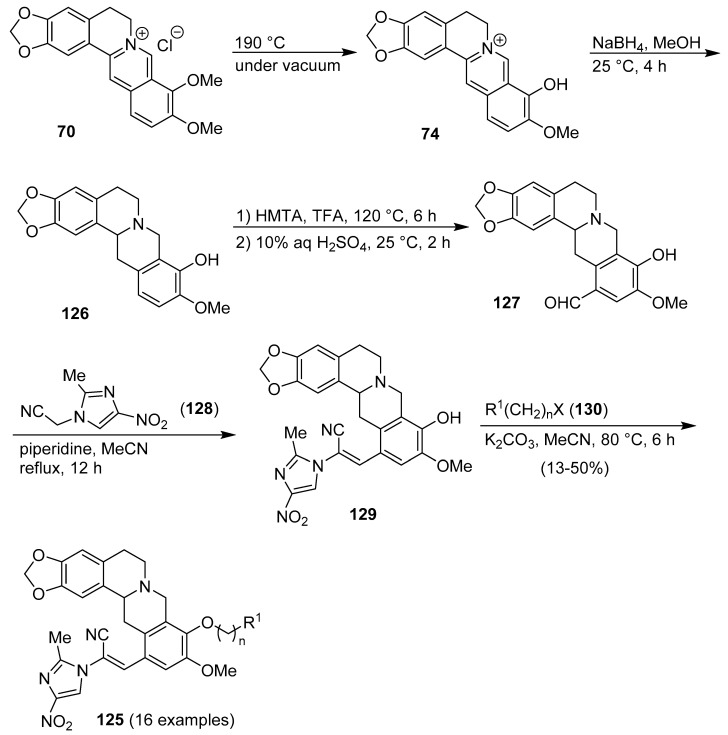
Synthesis of berberine/nitroimidazole hybrids **125.**

In 2018, Cai, Zhou, and coworkers synthesised berberine/nitroimidazole hybrids **125** via the reaction sequence outlined in Scheme 23, in which berberine (**70**) was the starting material [69]. Berberrubine (**74**), which was obtained in 90% yield by selective demethylation of **70**, was reduced with NaBH_4_ in MeOH generating compound **126** in 57% yield. Formylation of **126** by treatment with HMTA in trifluoroacetic acid produced aldehyde **127**, which was condensed with 2-(2-methyl-4-nitro-1*H*-imidazol-1-yl)acetonitrile **128** in the presence of piperidine affording compound **129**. Finally, *O*-alkylation of the latter compound with alkyl/aryl halides **130** provided hybrids **125** in yields ranging from 13 to 50%. 

Cai, Zhou, and coworkers next evaluated the antibacterial activities of hybrids **125** against MRSA, *E. faecalis*, *S. aureus*, *S. aureus* ATCC 25923, *B. subtilis* ATCC 6633, *M. luteus* ATCC 4698, *K. pneumoniae*, *E. coli*, *E. coli* ATCC 25922, *P. aeruginosa*, *P. aeruginosa* ATCC 27853, and *A. baumannii* and found that, among these compounds, tetrahydroberberine-9-(2-fluorobenzyl)-12-[2-(2-methyl-5-nitroimidazolyl)acrylonitrile (**125p**) (R^1^ = 2-FC_6_H_4_, n = 1) a berberine/nitroimidazole hybrid with an improved aqueous solubility (4.54 ± 0.08 μg/mL), not only exhibited strong antibacterial activity against drug resistant *E. coli* with an MIC value of 3 µM and was 33 times more potent than norfloxacin, but also displayed low toxicity towards RAW 264.7 mouse cancer cells. Cai, Zhou, and coworkers also investigated the antibacterial mechanism and found that compound **125p** might target *E. coli* DNA polymerase II through the formation of hydrogen bonds, effectively permeability-resistant *E. coli* cell membrane, and intercalate into DNA isolated from resistant *E. coli* to form **125p**–DNA complex, thus blocking DNA replication [69]. They also reported that resistance in *E. coli*, *Klebsiella* spp., and *Enterobacter* spp. is mainly related to the production of extended-spectrum β-lactamase, but other resistance mechanisms are also possible [69].

**Scheme 24:**
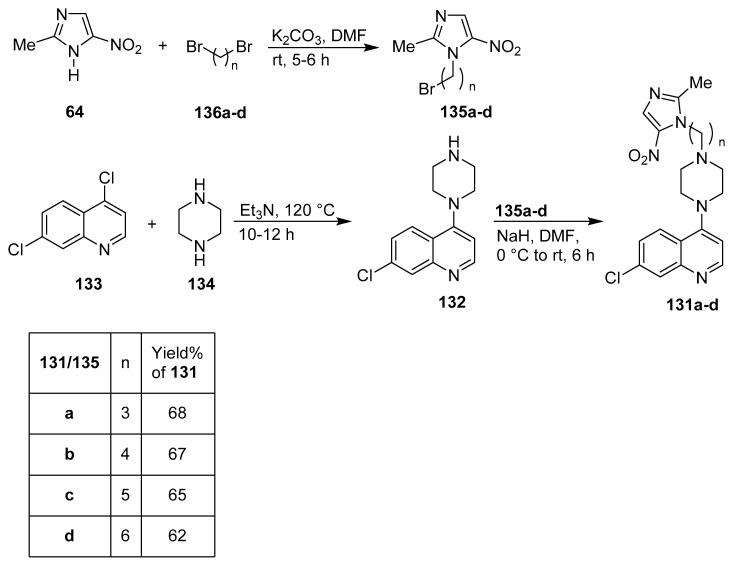
Synthesis of nitroimidazole/7-chloroquinoline conjugates **131a**–**d.**

Still in 2018, studies on the synthesis and evaluation of the antimicrobial activity of molecular hybrids and conjugates containing nitroimidazole moiety were also carried out by other research teams. Kumar and coworkers [70], in continuation of their studies on the synthesis of new anti-tubercular scaffolds [71,72], synthesised nitroimidazole/7-chloroquinoline conjugates **131a**–**d** (Scheme 24) and assessed both their activities towards *Mycobacterium tuberculosis* and their cytotoxicity towards the J774 murine macrophage cell line. 

The synthesis of compounds **131** began with the preparation of 4-piperazinyl-7-chloroquinoline (**132**) by the site-selective reaction of 4,7-dichloroquinoline (**133**) with a molar excess of piperazine (**134**) in Et_3_N at 120 °C for 10–12 h [73]. Subsequently, a solution of 1 eq of **132** was treated at 0 °C with a suspension of 2 eq of NaH in DMF, followed by the addition of 1.3 eq of 1-(bromoalkyl)-2-methyl-5-nitro-1*H*-imidazoles **135**. The resulting mixture was stirred at room temperature for 2 h affording conjugates **131a**–**d** in 62–68% yield. Compounds **135** were in turn prepared by reaction of 1 eq of 2-methyl-5-nitro-1*H*-imidazole (**64**) with 1.2 eq of the required 1,ω-dibromoalkanes **136** in DMF at room temperature in the presence of 2 eq of K_2_CO_3_ [70].

The anti-mycobacterial properties of conjugates **131** were subsequently evaluated, and it was found that the activity of these compounds was not higher than that of the standard drug isoniazid. Nevertheless, these compounds had appreciable activity with minimal cytotoxicity. 7-Chloro-4-{4-[4-(2-methyl-5-nitro-1*H*-imidazol-1-yl)butyl]piperazin-1-yl}quinoline (**131b**) proved to be the most potent among these conjugates with a IC_50_ value of 5.1 µM [70]. 

In the same year, Zhou and coworkers [74] investigated the antibacterial activities of naphthalimide/nitroimidazole hybrids **137a**–**d** and **138a**–**j** towards resistant *A. baumannii*, a clinically important nosocomial aerobic Gram-negative pathogen that can cause various infective diseases [75]. The synthesis of hybrids **137** and **138** was designed taking into account that: (i) naphthalimides can effectively inhibit the bacterial growth and that these compounds, in combination with nitrogen heterocycles, exhibit potent antimicrobial activities also towards bacterial resistant strains [76,77,78,79]; and (ii) nitroimidazoles are a class of antimicrobial drugs that have a remarkable spectrum of activity against anaerobic Gram-positive and Gram-negative bacteria [16].

**Scheme 25:**
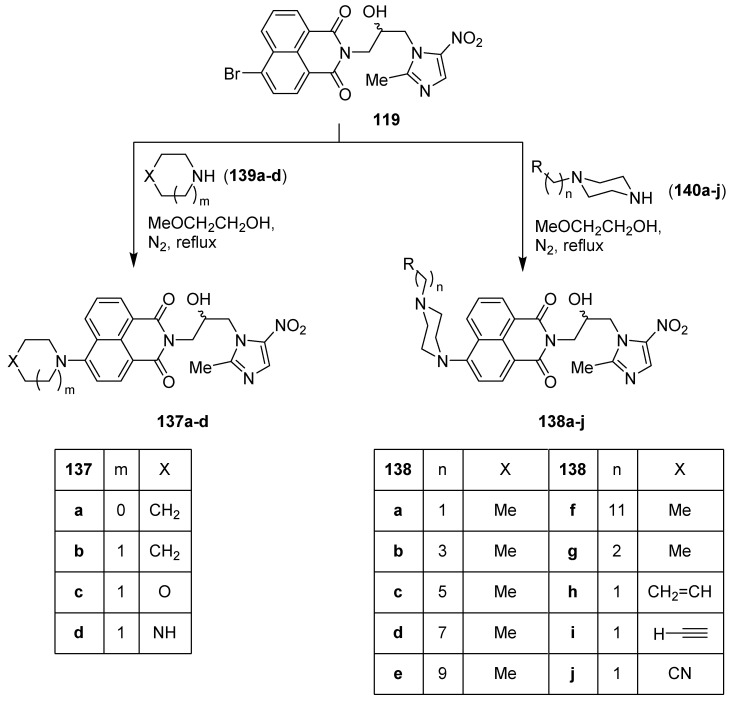
Synthesis of naphthalimide/nitroimidazole hybrids **137a**–**d** and **138a**–**j.**

Naphthalimide/nitroimidazole hybrids **137a**–**d** and **138a**–**j** were synthesised in yields ranging from 30 to 60% by the route outlined in Scheme 25. In particular, compound **119**, which was prepared from commercially available **113** according to the protocol illustrated in Scheme 21, was treated with alicyclic amines **139a**–**d** in 2-methoxyethanol under reflux for 4 h to afford hybrids **137a**–**d**. Instead, hybrids **138a**–**j** were prepared by reaction between **119** and *N*-alkylpiperazines **140** in 2-methoxyethanol under reflux and N_2_ atmosphere [74].

Hybrids **137** and **138** were then tested for their in vitro antibacterial activities against five Gram-positive bacterial strains (MRSA, *S. aureus*, *S. aureus* ATCC 25923, *S. aureus* ATCC 29213, and *E. faecalis*) and six Gram-negative bacterial strains (*E. coli*, *E. coli* ATCC 25922, *K. pneumoniae*, *P. aeruginosa*, *P. aeruginosa* ATCC 27853, and *A. baumannii*). The obtained results showed that these hybrids were able to inhibit the growth of the tested strains and that their effectiveness, except that of compound **137a**, was better than that of compound **119**. It was also found that hybrid **138e** showed high antibacterial activity (MIC = 13 µM) against resistant *A. baumannii* with rapid killing effect and no obvious resistance development [74]. Furthermore, it was discovered that when **138e** was used in combination with chloramphenicol [50], norfloxacin, or clinafloxacin, the antibacterial potency of this compound against resistant *A. baumannii* was improved [74].

In 2019, Zang, Zhang, Zhou, and coworkers turned their attention to the development of novel compounds possessing the ability to combat the resistance of Gram-positive pathogen MRSA [80]. The resistance is due to the acquisition of *mecA* gene, which, unlike of any PBP (penicillin binding protein) normally produced by *S. aureus*, encodes the protein PBP2a that has a low affinity for β-lactam antibiotics such as penicillin and methicillin [80]. In particular, the above-mentioned researchers directed their efforts towards the development of novel structural candidates of enone-bridged indole/nitroimidazole scaffolds. In fact, satisfactory antimicrobial activity of this type of conjugates had already been highlighted [81]. Furthermore, it was kept in mind that α,β-unsaturated carbonyl derivatives are linkers commonly used as functional structures for drug design [82].

**Scheme 26:**
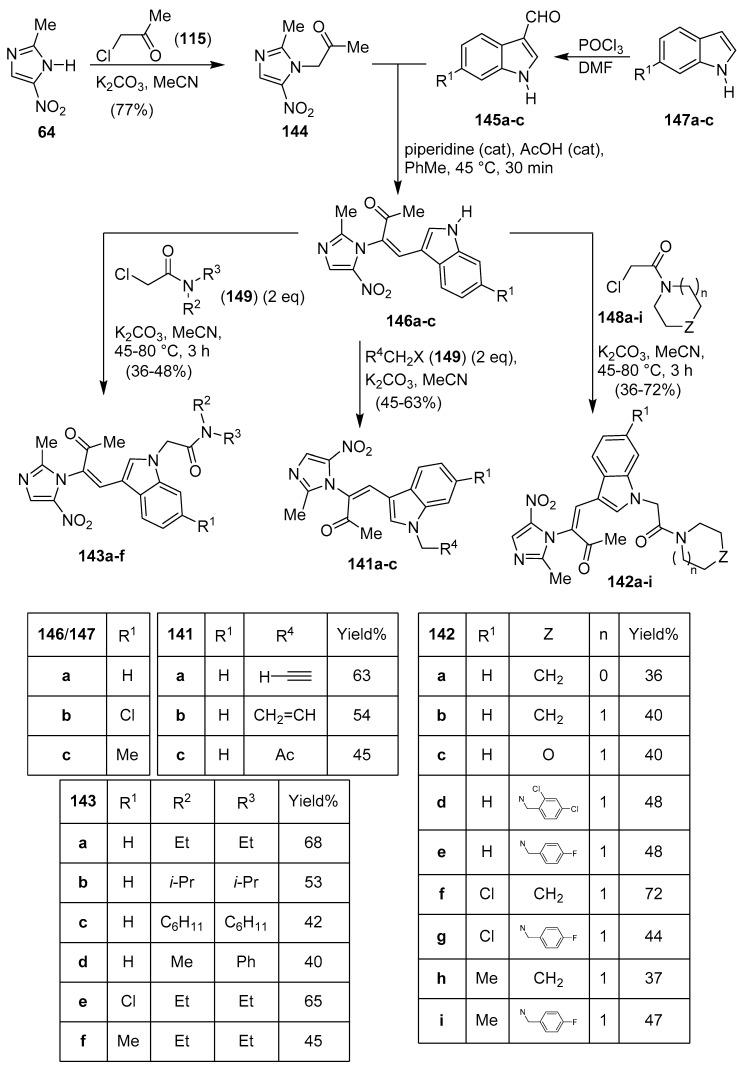
Synthesis of enone bridged indole/nitroimidazole conjugates **141a**–**c**, **142a**–**i**, and **143a**–**f.**

The target enone bridged indole/nitroimidazole conjugates **141a**–**c**, **142a**–**i**, and **143a**–**f** were synthesised as outlined in Scheme 26. In particular, 2-methyl-5-nitro-1*H*-imidazole (**64**) was treated with chloroacetone (**115**) in the presence of K_2_CO_3_ to give compound **144** in 77% yield. The reaction of **144** with 1*H*-indoles-3-carbaldehydes **145a**–**c** in toluene using piperidine and AcOH as catalysts afforded compounds **146a**–**c**, which were used as key intermediates in the synthesis of conjugates **141**, **142**, and **143**. Compounds **145a**–**c** were in turn obtained by the Vilsmeier-Haack reaction of indoles **147a**–**c**. Treatment of intermediates **146** with the chloroacetyl derivatives **148** and **149** in MeCN in the presence of K_2_CO_3_ afforded conjugates **142** and **143**, respectively. Compounds **143a**–**f** were obtained in 36–48% yield and compounds **142a**–**i** were prepared in yields ranging from 36 to 72%. Indole/nitroimidazole conjugates **141a**–**c** were instead prepared in satisfactory yields by reaction of **146** with the appropriate alkyl halides **130** in MeCN in the presence of K_2_CO_3_ [80].

The results of the evaluation of the antimicrobial activity of conjugates **141a**–**c**, **142a**–**i**, and **143a**–**f** showed that most of these conjugates displayed inhibitory efficiency towards Gram-positive bacteria. It was also discovered that (*E*)-3-(2-methyl-5-nitro-1*H*-imidazol-1-yl)-4-{1-[2-oxo-2-(piperidin-1-yl)ethyl]-1*H*-indol-3-yl}but-3-en-2-one (**142b**) possessed a satisfactory inhibitory activity on MRSA (MIC = 2 μM) and could effectively prevent the development of bacterial resistance, could intercalate into DNA, and also permeate MRSA membrane. It is worth noting that this hybrid also exhibited low cytotoxicity towards normal lung epithelial cell line BEAS-2B [80].

Concluding this section of the review it finally deserves to be mentioned that, in 2016, Kedar and coworkers designed and synthesised a set of new double nitroimidazoles and that one of these compounds, bis[1-(2-methyl-5-nitro-1*H*-imidazol-1-yl)propan-2-yl]-2,2′-(piperazine-1,4-diyl) diacetate (**150**) exhibited significant antibacterial activity against *E. coli* and *S. aureus* higher than those evaluated for other tested compounds [83].

**Scheme 27:**
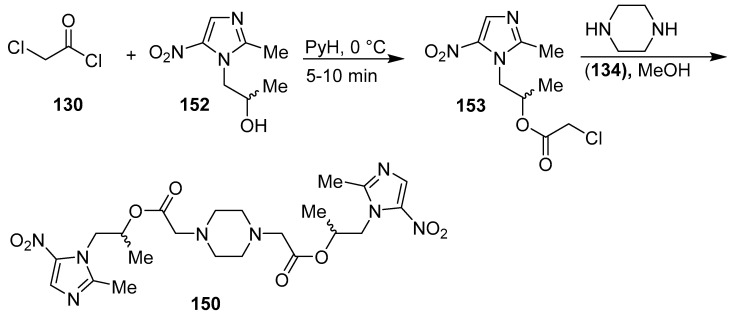
Synthesis of double nitroimidazole **150.**

Compound **150** was synthesised by reaction of chloroacetyl chloride (**151**) with secnidazole [1-(2-methyl-5-nitro-1*H*-imidazol-1-yl)propan-2-ol] (**152**) in pyridine at 0 °C for 5–10 min, followed by treatment of the resulting compound **153** with piperazine (**134**) in MeOH (Scheme 27). Compound **152** is an antibiotic approved in the USA by the FDA for the treatment of bacterial vaginosis in adult women.

The zones of inhibition of **150** against *E. coli* at the concentrations of 50, 100, 300, and 500 μg/mL were 2, 7, 10, and 14 mm, respectively, while those of ofloxacin, the standard drug, were 13, 16, 24, and 31 mm, respectively. On the other hand, the zones of inhibition of **150** against *S. aureus* at the concentrations of 100, 300, and 500 μg/mL were 5, 12, and 14 mm, respectively, while those of ofloxacin were 16, 20, and 28 mm, respectively [83].

## 3. Coumarin/Imidazole Hybrids and Conjugates

Synthetic and naturally occurring coumarins have been the subject of various studies concerning their antibacterial properties [84,85,86,87,88,89,90] and it has been observed that although these compounds do not display relevant activity (MIC ≥ 128 μg/mL), they are capable of modulating the antibiotic resistance through a mechanism that most likely involves inhibition of bacterial pump efflux [91]. Furthermore, in recent years, in order to enhance the antibacterial activities of these substances, coumarin/imidazole hybrids and conjugates have been designed, synthesised, and tested in vitro.

**Scheme 28:**
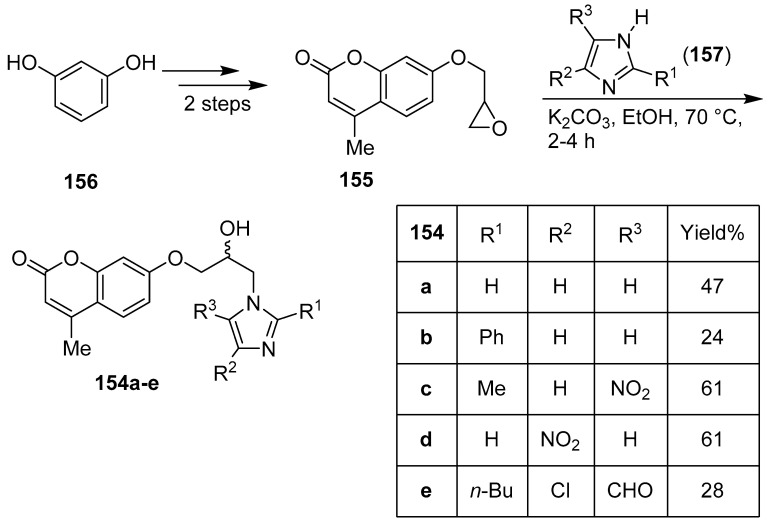
Synthesis of hybrids **154a**–**e.**

In 2016, Zhou and coworkers [92] synthesised and characterised 7-[2-hydroxy-3-(1*H*-imidazol-1-yl)propoxy]-4-methyl-2*H*-chromen-2-ones **154a**–**e** via the route shown in Scheme 28, in which compound **155**, which was prepared in two steps form resorcinol (**156**), was the key intermediate. Treatment of **155** with the appropriate imidazoles **157** in EtOH using K_2_CO_3_ as base afforded hybrids **154** in yields ranging from 24 to 61%.

These compounds were next tested for their antibacterial activities against MRSA, *B. subtilis*, *M. luteus* ATCC 4698, *E. coli* JM 109, *P. aeruginosa* ATCC 27853, and *S. dysenteriae* and it was found that **154a**–**c** and **154e** did not display significant antibacterial properties. Instead, hybrid **154d** exhibited activity against the tested Gram-positive bacteria with an MIC value of 93 μM against MRSA, *B. subtilis*, and *M. luteus.* Noticeably, the MIC value of **154d** against *B. subtilis* turned out to be comparable to that of the reference drug chloramphenicol (99 μM) but higher than that of norfloxacin (MIC = 13 μM) [92].

**Scheme 29:**
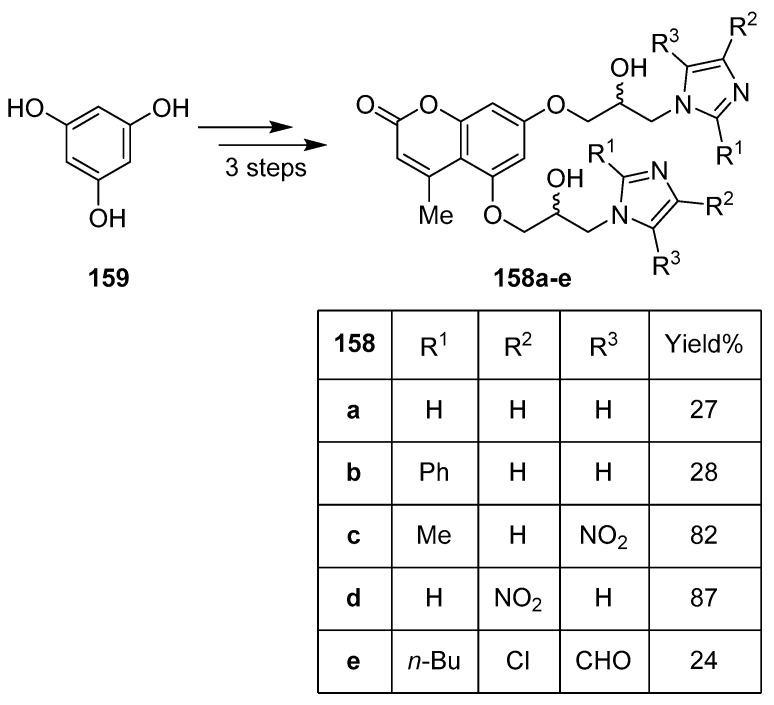
Synthesis of hybrids **158a**–**e.**

Zhou and coworkers also prepared compounds **158a**–**e** bearing bis-imidazolyl ethanol groups starting from phloroglucinol (**159**) (Scheme 29) via a three-step route, but found that these substances did not show appreciable antibacterial activity against the above-mentioned bacterial pathogens [92].

**Scheme 30:**
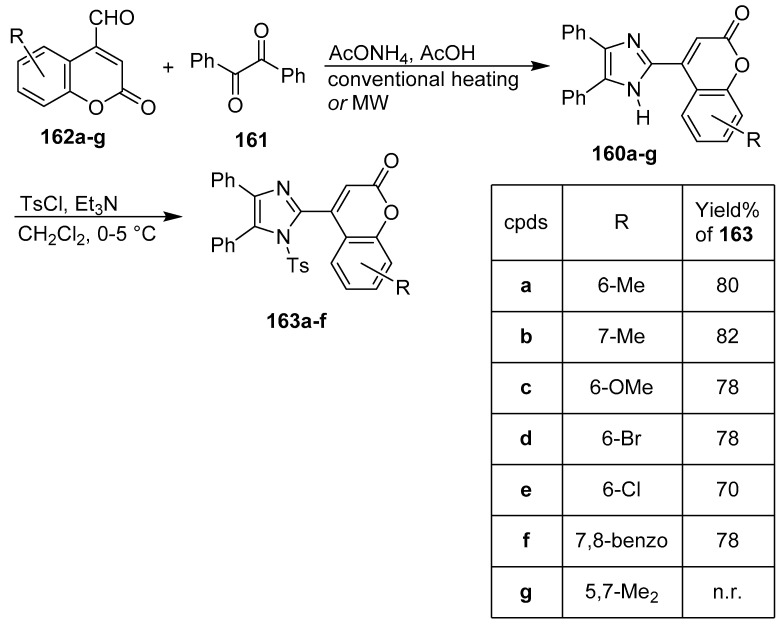
Synthesis of conjugates **160a**–**g** and **163a**–**f**.

In 2018, Shastri and coworkers [93] synthesised 4-(4,5-diphenyl-1*H*-imidazol-2-yl)-2*H*-chromen-2-ones **160a**–**g** in good to excellent yields by cyclocondensation of benzil (**161**) with 4-formylcoumarins **162a**–**g** [94] and AcONH_4_ in AcOH using both conventional heating conditions and microwave irradiation (Scheme 30). As expected, the yields of the microwave promoted cyclocondensation reactions were higher than those of the reactions carried out using conventional heating conditions. Hybrids **160a**–**f** were then converted to 4-(4,5-diphenyl-1-tosyl-1*H*-imidazol-2-yl)-2*H*-chromen-2-ones **163a**–**f** by treatment with 1.5 eq of TsCl in CH_2_Cl_2_ at 0–5 °C in the presence of Et_3_N (Scheme 30).

Conjugates **160** and **163** were then evaluated for their antimicrobial activities against Gram-positive *Bacillus flexus* and Gram-negative *Pseudomonas* spp. bacterial strains using ciprofloxacin (MIC = 2.1 μM) as the reference drug. Hybrids **163** were found to have MIC values lower than those of the corresponding conjugates **160** and compounds **163d** and **163f** had an MIC value 300–400 nM against both *B. flexus* and *Pseudomonas* spp. Instead, the MIC values the corresponding conjugates **160d** and **160f** against *B. flexus* were 0.9 and 1.4 μM, respectively, and against *Pseudomonas* spp., they were 0.9 and 1.2 μM, respectively [93].

**Figure 7:**
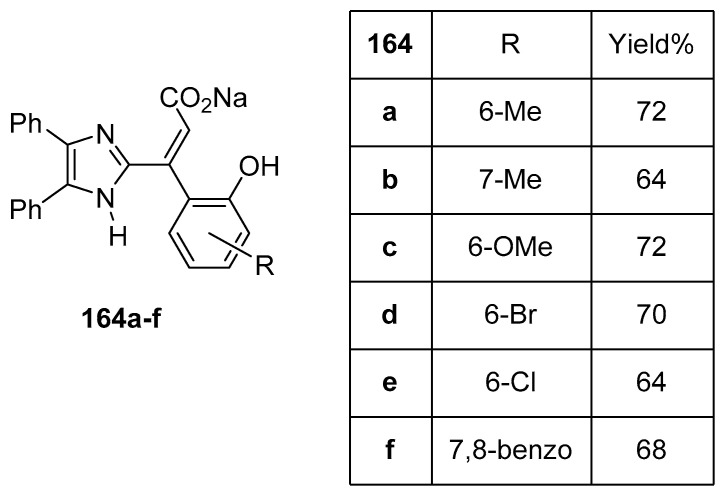
Structure of sodium (*Z*)-4-(2-hydroxyphenyl)-4-(4,5-diphenyl-1*H*-imidazol-2-yl)but-3-enoates **164a**–**f.**

It was finally observed that sodium (*Z*)-4-(2-hydroxyphenyl)-4-(4,5-diphenyl-1*H*-imidazol-2-yl)but-3-enoates **164a**–**f** (Figure 7), which were obtained by treatment of conjugates **160a**–**f** with 25% NaOH in EtOH at 80 °C, were, on a molar basis, highly potent and effective as hybrids **163** against both bacterial strains [93].

**Scheme 31:**
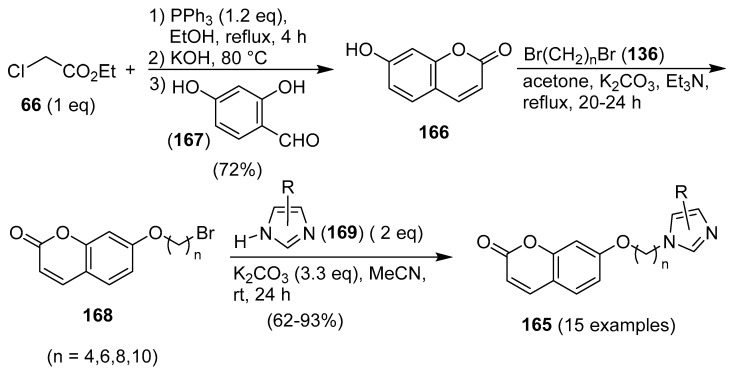
Synthesis of coumarin derivatives **165** containing imidazole skeleton.

Still in 2018, Wang and coworkers synthesised numerous coumarin derivatives containing imidazole skeleton and tested twenty-two of these hybrids of general formula **165** for their antibacterial activities against two Gram-negative bacteria, *E. coli* and *Flavobacterium columnare*, and two Gram-positive bacteria, *S. aureus* and *S. agalactiae*, using the fluoroquinolone antibiotics enrofloxacin and norfloxacin as reference drugs [95]. 7-Hydroxycoumarin (**166**), which was used as starting material in the synthesis of compounds **165** was prepared in 72% yield by reaction of ethyl chloroacetate (**66**) with PPh_3_ in EtOH, followed by treatment with 2,4-dihydroxybenzaldehyde (**167)** and KOH at 80 °C (Scheme 31).

The subsequent reaction of **166** with K_2_CO_3_ in acetone under reflux in the presence of Et_3_N, followed by the addition of 2 eq of α,ω-dibromoalkanes **136** provided intermediate coumarin bromides **168**. Finally, the reaction of compounds **168** with 2 eq of 1*H*-imidazoles **169** and K_2_CO_3_ in MeCN at room temperature provided hybrids **165** (Scheme 31) [95].

Investigations into the antibacterial activities of these hybrids were then undertaken and the obtained results showed that compounds **165c** (n = 8; R = H), **165g** (n = 8, R = 2-Me), and **165k** (n = 8, R = 4-Me) exhibited remarkable activities against *F. columnare* and *S. agalactiae*. Hybrid **165g** (n = 8, R = 2-Me) with MIC value of 2 and 4 μM, respectively, turned out to be the most potent among the tested hybrids and its potency was comparable to or higher than that of enrofloxacin. However, this compound did not exhibit significant antibacterial activity against *E. coli.* By contrast, potent activity against *E. coli* was exhibited by hybrid **165f** (n = 6, R = 2-Me), which had an MIC value of 8 μM [95]. It was finally demonstrated that the potent antibacterial activities of the above-mentioned hybrids were probably correlated to their FabI and FabK inhibitory activities. FabI and Fabk are bacterial enoyl–acyl carrier protein (ACP) reductase that catalyse the rate limiting step of bacterial fatty acid biosynthesis [96].

**Scheme 32:**
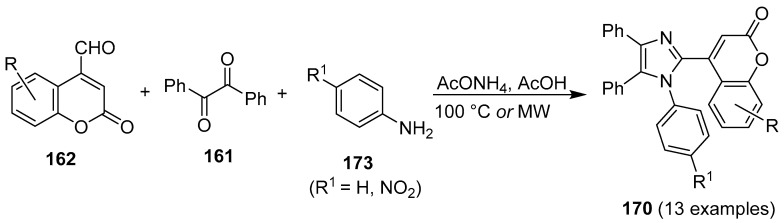
Synthesis of coumarin/imidazole conjugates **170.**

Finally, in 2020, Shastri and coworkers [97] continued their studies undertaken two years earlier on the synthesis and evaluation of the antimicrobial properties of coumarin/imidazoles conjugates and in this context they synthesised three new coumarin/imidazole conjugates of general formula **170**, **171**, and **172** using AcOH-catalysed four component cyclocondensation reactions and evaluated the antimicrobial activity of these compounds against Gram-positive *B. flexus* and Gram-negative *Pseudomonas* spp. bacterial strains. The optimised synthesis of conjugates **170** was carried out in good yields (> 65%) by treatment of 4-formylcoumarins **162** with benzil (**161)**, *p*-substituted anilines **173** and AcONH_4_ in the presence of AcOH using conventional heating at 100 °C as well under microwave irradiation (Scheme 32).

**Scheme 33:**
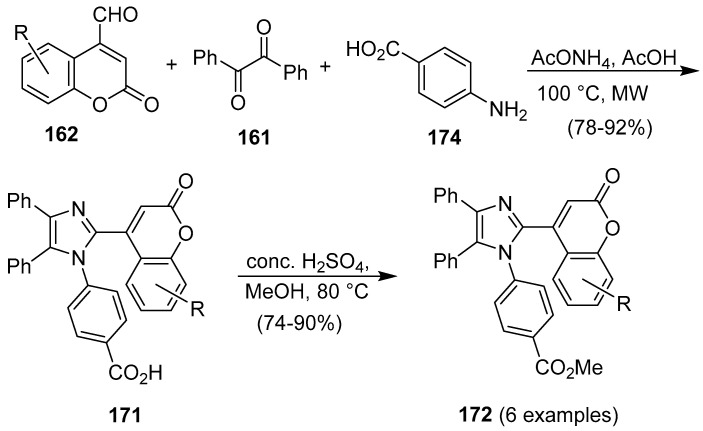
Synthesis of coumarin/imidazole conjugates **171** and **172.**

Coumarin/imidazole conjugates **172** were similarly synthesised in good to excellent yields by reaction of aldehydes **162** with benzil (**161**), *p*-aminobenzoic acid (**173**), and AcONH_4_ in AcOH at 100 °C (Scheme 33). Conjugates **171** were then converted to the corresponding methyl esters **172** by reaction with MeOH in the presence of conc. sulfuric acid (Scheme 33) [97].

The results of antibacterial assays showed that conjugates **170** bearing an unsubstituted 1-phenylimidazole moiety exhibited excellent antibacterial activities against both bacterial strains with MIC values ranging from 200 to 860 nM. On the other hand, coumarin/imidazole conjugates **171** bearing a 1-(4-carboxy-substituted phenyl) moiety exhibited a decrease in the bacterial activity, but when they were converted to the corresponding methyl esters **172** they revealed improved activity compared to the corresponding compounds **170** [97].

## 4. Furanchalcone/Imidazole Hybrids

In 2019, Araque, Cardona-G, and coworkers reported the synthesis and evaluation of the antimicrobial activity in silico of six methylimidazolium/furanchalcone hybrids **175** that are molecules with two structural domains having different biological functions [98]. Furans, which are constituents of the furanchalcone domain have been shown to exhibit significant antibacterial activity [99,100,101]. Furthermore, chalcone and its derivatives have proven to be potent antibacterial agents [102].

**Scheme 34:**
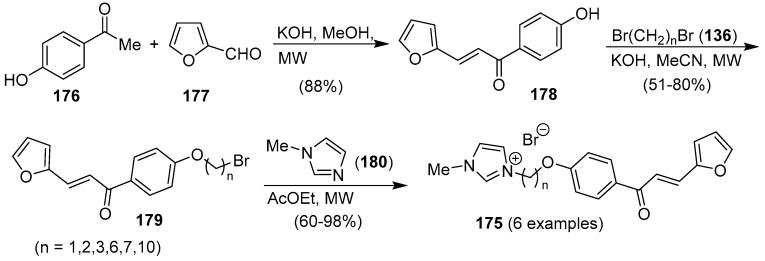
Synthesis of methylimidazolium/furanchalcone hybrids **175.**

As shown in Scheme 34, hybrids **175** were prepared by ultrasonic irradiation-assisted Claisen-Schmidt condensation reaction of 4-hydroxyacetophenone (**176**) with furfural (**177**), which provided (*E*)-3-furan-2-yl-1-(4-hydroxyphenyl)prop-2-en-1-one (**178**) in 88% yield. The subsequent microwave-promoted Williamson etherification of the latter compound with the appropriate α,ω-dibromoalkanes **136** afforded compounds **179** in 51–80% yields. Finally, microwave-assisted *N*-alkylation of 1-methyl-1*H*-imidazole (**180**) with bromides **179** provided the required hybrids **175** in 60–98% yield [98].

These compounds were then tested for their antimicrobial activities against the Gram-negative bacteria *E. coli* and *P. aeruginosa*, and the Gram-positive bacteria *S. aureus*, *S. agalactiae*, *Streptococcus mutans, Bacillus cereus*, and *B. subtilis* subsp. *spizizenii* and it was found among these hybrids compound **175d** (n = 6) exhibited high activity against *S. aureus* ATCC 25923 (IC_50_ = 14.6 ± 0.5 mM) and *S. mutans* (clinical isolate) (IC_50_ = 18.7 ± 0.7 mM). However, the antibacterial activities of hybrids **175** against all the tested bacterial strains were lower in comparison to that of the standard drug oxytetracycline (IC_50_ = 14.0 ± 0.7 µM), but they were better than those of the lead compounds, i.e., furanchalcone, 1-methylimidazolium, or their mixture [98].

## 5. Hybrids Based on Benzofuran, Quinazolinone, and Imidazolium Moieties

Over the past two decades, several benzofuran derivatives have been prepared and introduced as antibacterial agents [103]. A lot of research has also been conducted on the synthesis of quinazolinone derivatives with potent antimicrobial activity especially against Gram-positive strains [104,105].

**Scheme 35:**
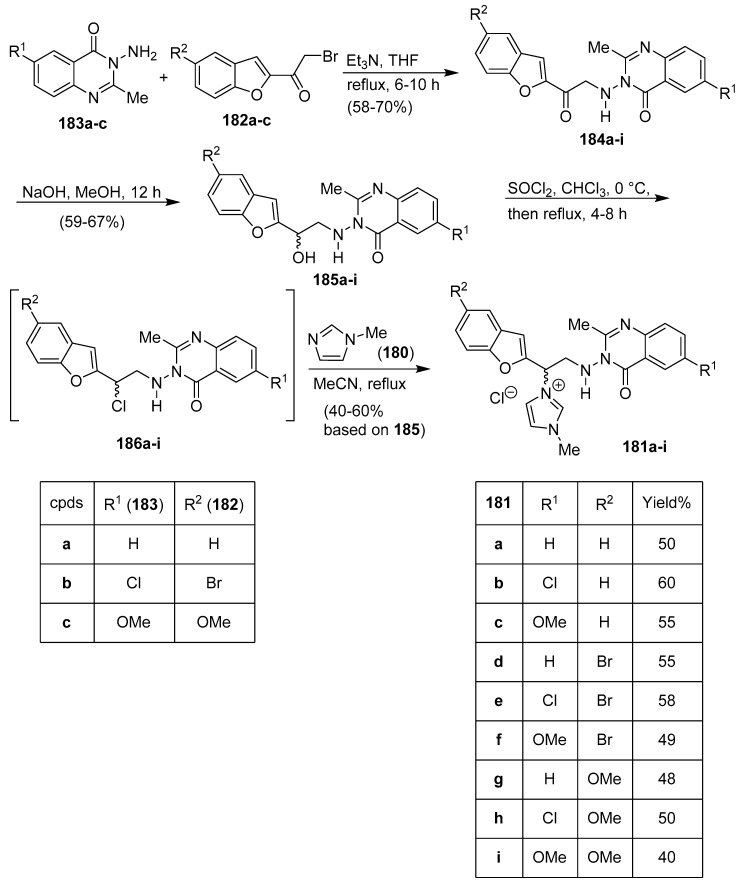
Synthesis of 3-{1-(benzofuran-2-yl)-2-[(2-methyl-4-oxoquinazolin-3(4*H*)-yl)amino] ethyl}-1-methyl-1*H*-imidazol-3-ium chlorides **181a**–**i.**

In 2017, Khodarahmi and coworkers hypothesised that the potency of these pharmacophores could be enhanced by incorporating them into hybrids containing imidazolium moieties [106]. Thus, they synthesised and characterised 3-{1-(benzofuran-2-yl)-2-[(2-methyl-4-oxoquinazolin-3(4*H*)-yl) amino]ethyl}-1-methyl-1*H*-imidazol-3-ium chlorides **181a**–**i** and tested their in vitro activities against three Gram-positive bacteria (*S. aureus* PTCC 1023, *B. subtilis* PTCC 1023, and *Listeria monocitogenes* PTCC 1165) and three Gram-negative bacteria (*E. coli* PTCC 1338, *P. aeruginosa* PTCC 1074, and *Salmonella* Enteritidis (namely, *S. enterica* subsp*. enterica* sv. Enteritidis) PTCC 1091) using fluoroquinolone antibiotic ciprofloxacin as the reference compound. Hybrids **181a**–**i** were synthesised via the route outlined in Scheme 35.

**Scheme 36:**
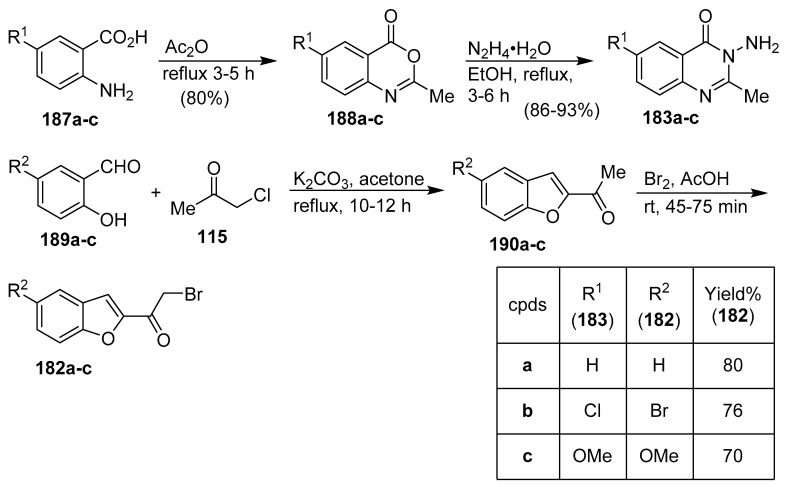
Synthesis of compounds **183a**–**c** and **182a**–**c.**

In particular, a solution of 1-(benzofuran-2-yl)-2-bromoethan-1-one derivatives **182** in THF was treated with a mixture of quinazolin-4(3*H*)-ones **183**, Et_3_N, and THF under reflux for 6–10 h and the resulting 3-{[2-(benzofuran-2-yl)-2-oxoethyl]amino}-2-methylquinazolin-4(3*H*)-ones **184** were reduced with NaBH_4_ in MeOH at room temperature for 12 h affording intermediates **185** in yields ranging from 59 to 67%. Treatment of the latter compounds with SOCl_2_ in CHCl_3_ under reflux for 4–8 h provided 3-{[2-(benzofuran-2-yl)-2-chloroethyl]amino}-2-methylquinazolin-4(3*H*)-ones **186**, which finally were converted to hybrids **181** in 40–60% yield based on compounds **185** by reaction with 1-methyl-1*H*-imidazole (**180**) in MeCN under reflux for 24–36 h. Compounds **183** were in turn prepared by reaction of anthranilic acids **187** with Ac_2_O under reflux for 3–8 h, followed by treatment of the resulting 2-methyl-4*H*-benzo[*d*][1,3]oxazin-4-ones **188** with a solution of hydrazine hydrate in EtOH under reflux for 3–6 h (Scheme 36). On the other hand, the preparation of compounds **182** was carried out by treatment of salicylaldehydes **189** with chloroacetyl chloride (**115**) in acetone under reflux in the presence of K_2_CO_3_, followed by reaction of the resulting (benzofuran-2-yl)ethanones **190** with a solution of bromine in AcOH (Scheme 36) [106].

Hybrids **181** were then tested for their antimicrobial activities against the above-mentioned strains of microorganisms, and hybrid **181e** turned out to be the most active against *S. aureus* and *B. subtilis* with MIC values of 29 and 58 μM, respectively, which were, however, higher than those of ciprofloxacin. The MIC values of this antibiotic against these two bacterial species were 12 and 24 μM, respectively. All other hybrids **181** had MIC values against the tested bacterial strains that ranged from 230 to 1200 μM [106].

## 6. 1*H*-Imidazoles Containing Azetidin-2-one (β-lactam) Derivatives

Azetidin-2-one (β-lactam) antibiotics have been widely used for treatment of a wide variety of infections mainly caused by aerobic Gram-negative bacteria [107,108,109]. These drugs, which act by binding to and inactivating the enzymes required for bacterial cell wall synthesis, have been the subject of several reviews concerning their structures, synthesis, and mode of action [110,111,112,113]. However, for some time a rapid appearance of a great number of bacteria presenting resistance to these agents has been observed and numerous investigations have been conducted in order to elucidate and counter these phenomena of resistance [114,115,116,117].

**Scheme 37:**
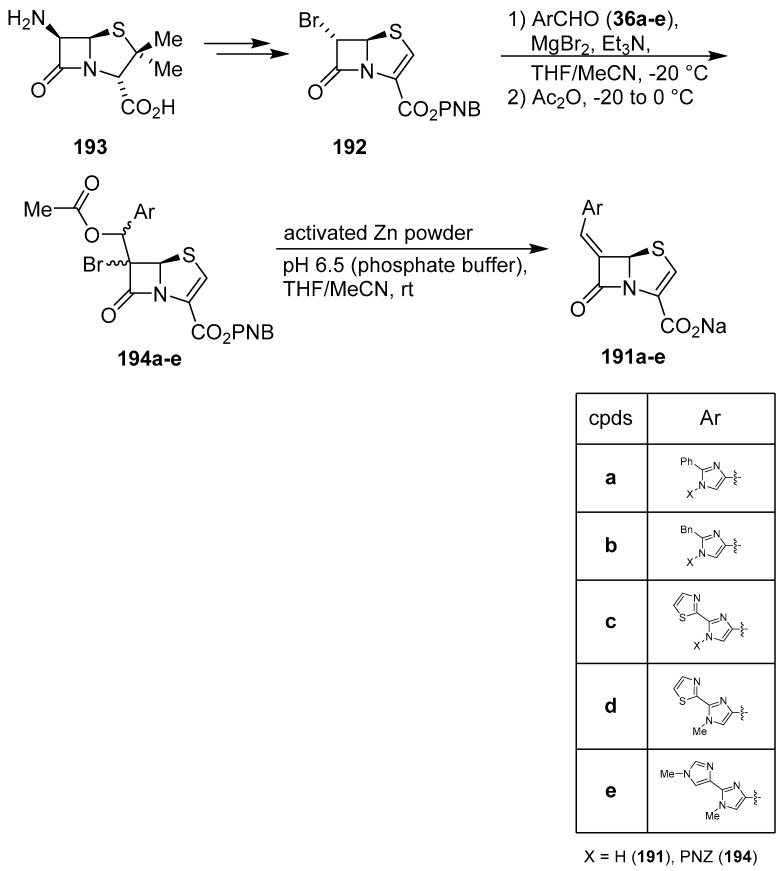
Synthesis of imidazole substituted 6-methylidene-penems **194a**–**e** and **191a**–**e.**

β-Lactamases, which are serine-dependent enzymes produced by the bacteria in defence against all classes of β-lactam antibiotics, particularly in Gram-negative bacteria are a major determinant of resistance [118]. Production of class-A, class-B, and class-C enzymes by the bacteria causes inefficiency in some cases of β-lactam antibiotics. In order to overcome this resistance, several β-lactamase inhibitors have been developed and used in clinics in combination with β-lactam antibiotics. In this context, in 2004, Venkatesan and coworkers synthesised 6-methylidene-penem carboxylic acid sodium salts **191a**–**e** (Scheme 37) as broad-spectrum β-lactamase inhibitors and tested these compounds against various β-lactamase producing isolates [119].

Sodium salts **191a**–**e** were synthesised by a process (Scheme 37), in which 4-nitrobenzyl (5*R*,6*S*)-6-bromo-7-oxo-4-thia-1-azabicyclo[3.2.0]hept-2-ene-2-carboxylate (**192**), which was used as the starting material, was prepared from commercially available 6-aminopenicillanic acid (**193**) by a multistep procedure reported in the literature [120].

The anhydrous MgBr_2_-mediated aldol condensation reaction between **192** and the appropriately substituted aldehydes **36a**–**c** in MeCN at −20 °C in the presence of Et_3_N under N_2_ atmosphere followed by treatment with Ac_2_O in one portion, warming to 0 °C, and stirring for 27 h provided acetoxy bromohydrins **194**. Finally, the latter compounds were treated with activated Zn powder activated in an amount that was four times the weight of the substrate bromohydrins. The reaction, which was carried out at room temperature in a mixture of MeCN, THF (1:2), and 0.5 M phosphate buffer (pH 6.5) led to the (*Z*)-stereoselective formation of compounds **191**. Interestingly, this elimination reaction with a rather surprising stereochemical result also involved the deprotection of the carboxyl functionality of compounds **194** [119].

Methylidene-penem derivatives **191a**–**e** were next tested in vitro against TEM-1 β-lactamase, Imi-1 (class A), CerA (class B), and AmpC (class C) enzymes for their inhibitory ability and, except for compound **191e**, which was found to be less active against Imi-1, these compounds turned out to be potent inhibitors of both class-A and class-C enzymes. Interestingly, they had a spectrum of activity that was broader than that of the inhibitors then available in the market. It was also found that compound **191b** in vivo enhanced the activity of piperacillin (a broad-spectrum β-lactam antibiotic of the ureidopenicillin class) against *E. coli* LSU 80-8, a TEM-1 producing organism. The in vivo ED_50_ value of piperacillin and **191b** (4:1 ratio) in a murine acute lethal infection model with *E. coli* LSU 80-8 was 43 ± 5 mg/kg, while the ED_50_ value of piperacillin alone was >128 mg/kg [119].

**Figure 8:**
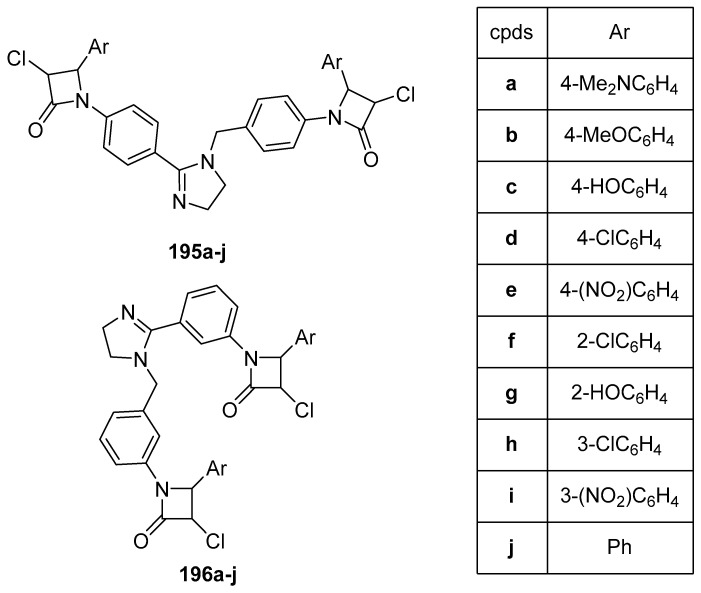
Structures of imidazole containing bisazetidinones **195** and **196.**

Some years after this study, Pagadala and coworkers described a practical and efficient synthesis of imidazole containing bisazetidinones **195a**–**j** and **196a**–**j** (Figure 8) and evaluated the in vitro antibacterial activity of these compounds against the Gram-positive bacteria *B. subtilis*, *P. vulgaris*, and *S. aureus* and the Gram-negative bacteria *E. coli* and *K. pneumoniae* using the penicillin antibiotic ampicillin as the standard drug [121].

**Scheme 38:**
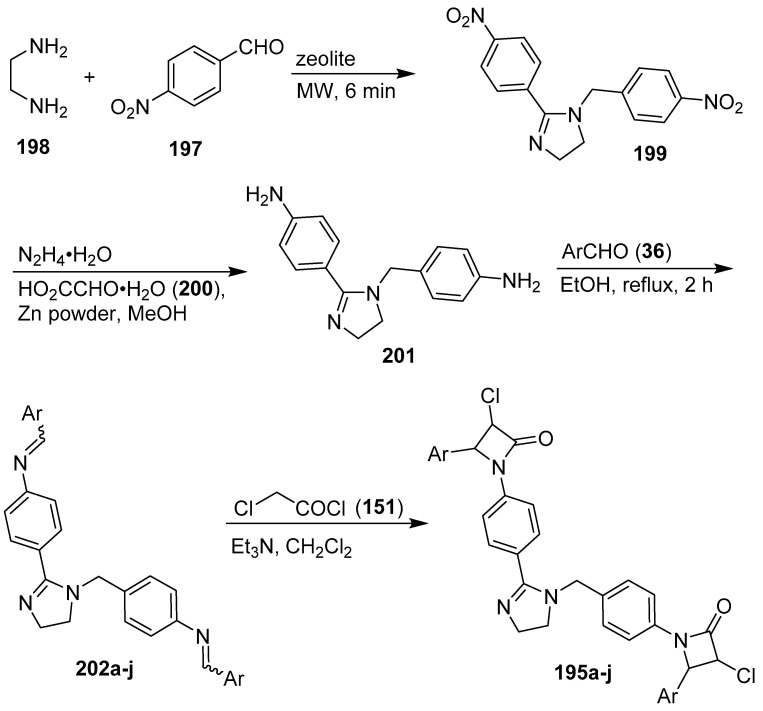
Synthesis of compounds **195a**–**j.**

Compounds **195a**–**j** were synthesised in excellent yields through the reactions illustrated in Scheme 38.

In accordance with a previously reported procedure [122], *p*-nitrobenzaldehyde (**197**) was treated with 1,2-diaminoethane (**198**) in the presence of zeolite under microwave irradiation in the absence of solvent to furnish compound **199** in a good yield. The nitro groups of this compound were then selectively reduced according to a literature procedure [123], which involved the reaction of this substrate with a suspension of zinc powder in MeOH at room temperature in the presence of hydrazine glyoxylate. The latter compound was prepared by neutralising slowly equal molar amounts of hydrazine hydrate and glyoxylic acid monohydrate (**200**). Condensation of the resulting diamine **201** with the appropriately substituted aryl aldehydes **36** in refluxing EtOH afforded bis-imines **202a**–**j**. Finally, the [2+2] cycloaddition reaction (wrongly named by the authors as a Staudinger reaction) that involved bis-imines **202a**–**j** and ketene generated in situ from chloroacetyl chloride (**151**) and Et_3_N provided bisazetidinones **196a**–**j** (Scheme 38) [121].

**Scheme 39:**
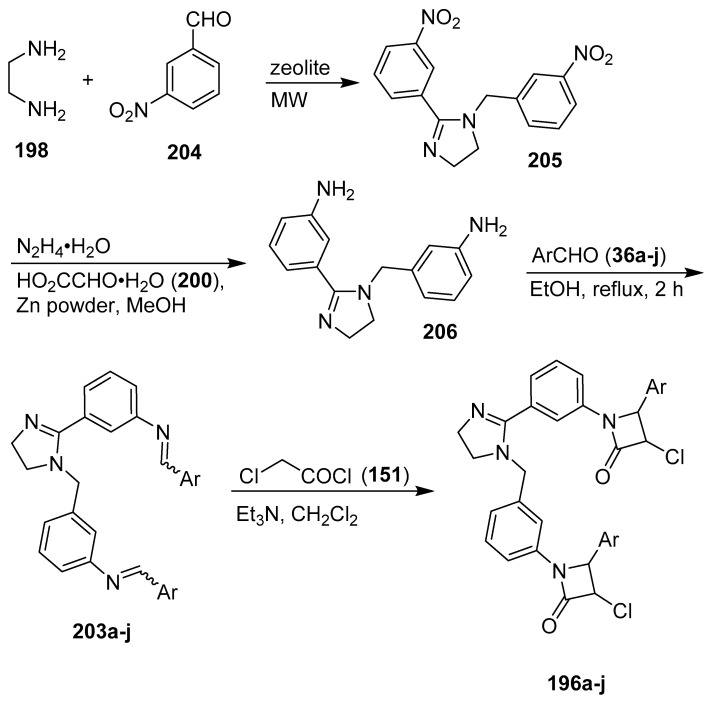
Synthesis of compounds **196a**–**j.**

On the other hand, a similar reaction sequence involving the [2+2] cycloaddition reaction of bis-imines **196a**–**j** and ketene generated in situ from chloroacetyl chloride (**151**) and Et_3_N, was employed for the synthesis of imidazole containing bisazetidinones **196a**–**j** in excellent yields (Scheme 39). Bis-imines **203a**–**j** were, in turn, prepared by reaction of **198** with 3-nitrobenzaldehyde (**204**) in the presence of zeolite under microwave irradiation and selective reduction of the nitro groups of the resulting compound **205** followed by condensation of the appropriate aldehydes **36** with diamine **206** resulting from the reduction reaction (Scheme 39) [121].

The antibacterial data of the synthesised imidazole containing bisazetidinones showed that at 100 µg/mL, compounds **195a, 195b**, and **196b** were highly active against *B. subtilis*. Compound **196a** was highly active against *P. vulgaris* and *S. aureus*, **195b** and **196c** proved to be highly active against *E. coli* and **195a** and **196b** displayed the high activity against *K. pneumoniae*, which turned out to be almost equivalent to that of ampicillin [121]. Instead, hybrids **195d, 196a**, **196c**, and **196d**, exhibited moderate activity against *B. subtilis* [121].

**Scheme 40:**
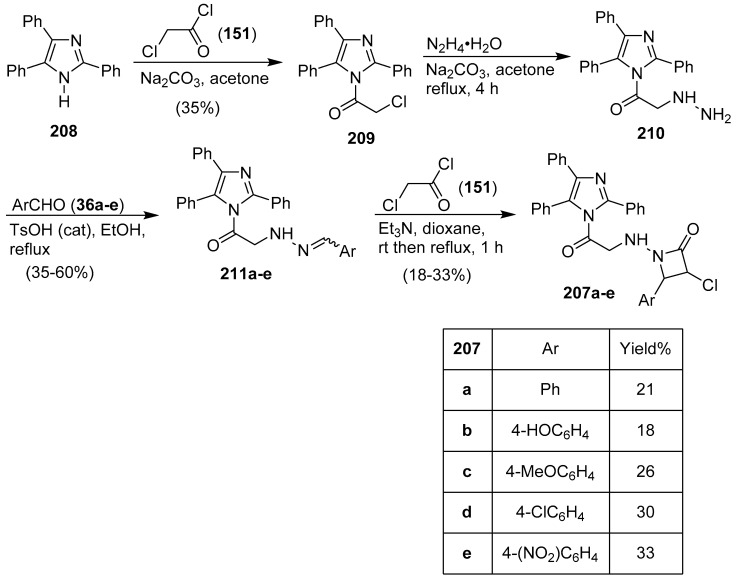
Synthesis of compounds **207a**–**e.**

In 2018, Noori and coworkers synthesised imidazole derivatives **207a**–**e** bearing β-lactam moiety and evaluated their antibacterial activity against *S. aureus*, *Enterococcus faecalis*, *E. coli*, *Streptococcus pyogenes*, and *K. pneumoniae* [124]. The synthesis of these hybrids, which in disagreement with Klahn’s definition [7] were named by Noori as conjugates, was carried out in a modest overall yield from lophine (2,4,5-triphenyl-1*H*-imidazole) (**208**) via the reaction sequence shown in Scheme 40.

Specifically, *N*-acylation of **208** (lophine) with chloroacetyl chloride (**151**) in acetone in the presence of anhydrous Na_2_CO_3_ afforded compound **209** in 35% yield. Subsequent treatment of **209** with 2 eq of hydrazine hydrate in acetone in the presence of anhydrous Na_2_CO_3_ provided compound **210** in 32% yield, which was converted into the Schiff base derivatives **211a**–**e** by reaction with the appropriate aldehydes **36** in EtOH under reflux in the presence of a catalytic amount of TsOH.

Compounds **211a**–**e**, which were obtained in 35–60% yield, were then treated with **130** in dioxane in the presence of Et_3_N providing the required hybrids **207a**–**e** in yields ranging from 18 to 33% [124].

Antibacterial activity tests showed that compound **207d** at a concentration of 900 µM was active against *E. coli* and *E. faecalis* with an inhibition zone of 15 mm and 20 mm, respectively, while the inhibition zone of the standard drug ceftazidime, a semisynthetic cephalosporin antibiotic, against these two bacterial species was 10 and 15 mm, respectively. However, **207d** did not exhibit antibacterial activity against *S. aureus* and *K. pneumoniae.* Good activity against all the bacterial species examined was instead displayed by hybrid **207c** (Ar = 4-MeOC_6_H_4_), which at a concentration of 900 µM had inhibition zones with diameters of 23, 20, 30, 42, and 37 mm against *S. aureus*, *K. pneumoniae*, *E. coli, E. faecalis*, and *S. pyogenes*, respectively [124].

## 7. Pyrrole/Imidazole and Indole/Imidazole Hybrids

**Figure 9:**
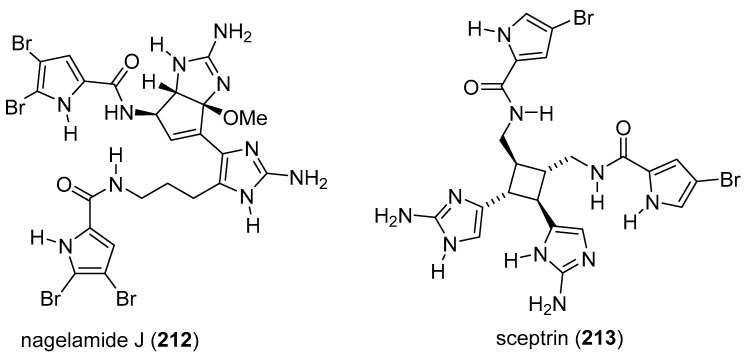
Structures of nagelamide J (**212**) and sceptrin (**213**).

Several pyrrole/imidazole hybrids have been isolated from marine sponges [125,126] and some of these alkaloids have been found to exhibit significant antimicrobial properties. Over the past two decades, several reviews summarising the isolation, synthesis, and biological properties of some of these substances have been published [126,127,128,129,130,131,132]. For example, nagelamide J (**212**) (Figure 9), was isolated in 2007 from an Okinawan marine sponge *Agelas* spp. and was found to exhibit antimicrobial activity against the yeast *Cryptococcus neoformans* with an MIC value of 21 μM [133]. However, to our knowledge, the total synthesis of this hybrid has not been reported so far.

Instead, many data on sceptrin (**213**) (Figure 9), which is the first naturally occurring dimeric pyrrole/imidazole hybrid, are available in literature. This compound, which was isolated in 1981 from the sponge *Agelas sceptrum* [134] and in 1993 from the South Pacific sponge *Agelas mauritiana* [135], was found to be an antimicrobial agent with a bacteriostatic rather than bactericidal effect on exponentially growing *E. coli* cells at the MIC [135]. Since 2004, numerous total syntheses of **213** have been reported in the literature [136,137,138,139,140]

**Scheme 41:**
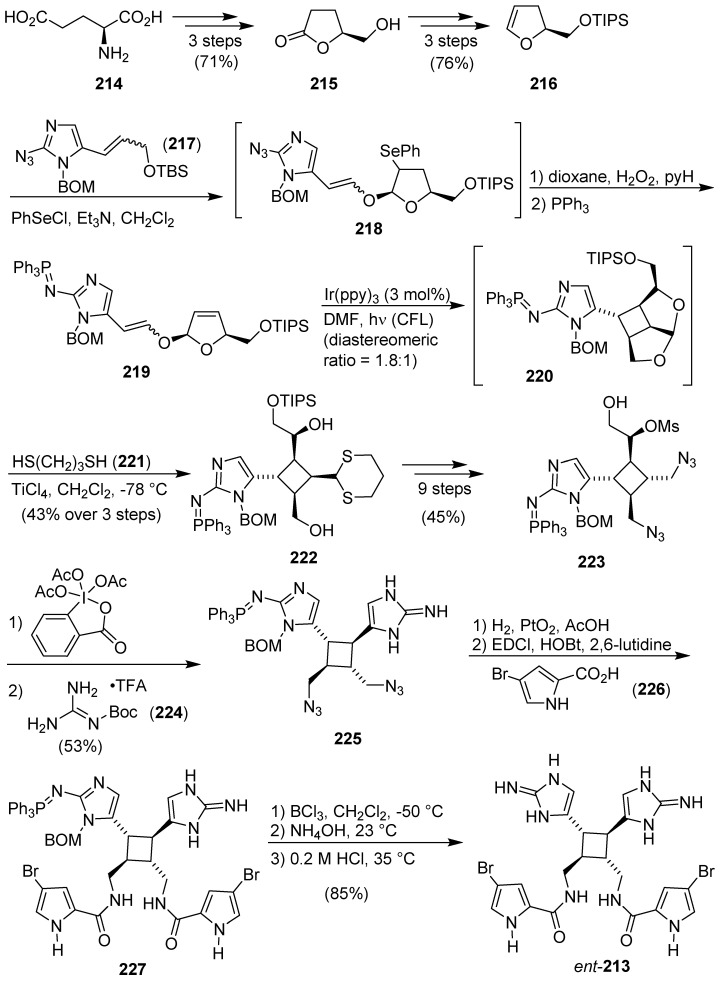
Total synthesis of *ent*-sceptrin (*ent*-**213**).

In 2014, Ma, Wang, and coworkers synthesised *ent*-**213** [140] while targeting the originally reported absolute stereochemistry of this alkaloid [134]. The multi-step synthesis of *ent*-**213** (Scheme 41), in which *L*-glutamic acid (**214**) was used as the source of chirality, involved the conversion of alcohol **215** to dihydrofuran **216** via a three-step reaction sequence. The subsequent electrophilic selenation and treatment with azidoimidazole **217** provided selenide **218**, which by oxidative elimination of the phenylselenyl group and treatment with PPh_3_ was converted to **219**. The unconventional [2+2] cycloaddition reaction of the latter compound according to the photoredox method developed by Yoon [141] provided cyclobutane **220**. In particular, the reaction involved treatment of a degassed DMF solution of **219** with tris[2-phenylpyridinate-*C^2^*,*N*]iridium(III) [Ir(ppy)_3_] (3 mol%) followed by irradiation with light generated from an 11 W CFL bulb at 23 °C. The resulting crude compound **220** was treated with propane-1,3-dithiol (**221**) and TiCl_4_ in CH_2_Cl_2_ at −78 °C affording dithiane **222** in 43% yield. A subsequent nine-step reaction sequence provided bis-azide **223**, which was oxidised using Dess-Martin periodinane. Treatment of the resulting unstable mesyl aldehyde with Boc-guanidine⋅TFA (**224**) provided aminoimidazole **225**. The azido groups of the latter compound were next reduced to amino groups, which were amidated by treatment with 4-bromo-1*H*-pyrrole-2-carboxylic acid (**226**) in DMF in the presence of EDC and HOBt. Finally, removal of the protecting groups of the resulting compound **227** provided *ent*-sceptrin in 0.4% yield based on *L*-glutamic acid (**213**) [140].

In 2014, Ma, Wang, and coworkers synthesised *ent*-**213** [140] while targeting the originally reported absolute stereochemistry of this alkaloid [134]. The multi-step synthesis of *ent*-**213** (Scheme 41), in which *L*-glutamic acid (**214**) was used as the source of chirality, involved the conversion of alcohol **215** to dihydrofuran **216** via a three-step reaction sequence. The subsequent electrophilic selenation and treatment with azidoimidazole **217** provided selenide **218**, which by oxidative elimination of the phenylselenyl group and treatment with PPh_3_ was converted to **219**. The unconventional [2+2] cycloaddition reaction of the latter compound according to the photoredox method developed by Yoon [141] provided cyclobutane **220**. In particular, the reaction involved treatment of a degassed DMF solution of **219** with tris[2-phenylpyridinate-*C^2^*,*N*]iridium(III) [Ir(ppy)_3_] (3 mol%) followed by irradiation with light generated from an 11 W CFL bulb at 23 °C. The resulting crude compound **220** was treated with propane-1,3-dithiol (**221**) and TiCl_4_ in CH_2_Cl_2_ at −78 °C affording dithiane **222** in 43% yield. A subsequent nine-step reaction sequence provided bis-azide **223**, which was oxidised using Dess-Martin periodinane. Treatment of the resulting unstable mesyl aldehyde with Boc-guanidine⋅TFA (**224**) provided aminoimidazole **225**. The azido groups of the latter compound were next reduced to amino groups, which were amidated by treatment with 4-bromo-1*H*-pyrrole-2-carboxylic acid (**226**) in DMF in the presence of EDC and HOBt. Finally, removal of the protecting groups of the resulting compound **227** provided *ent*-sceptrin in 0.4% yield based on *L*-glutamic acid (**213**) [140].

**Figure 10:**
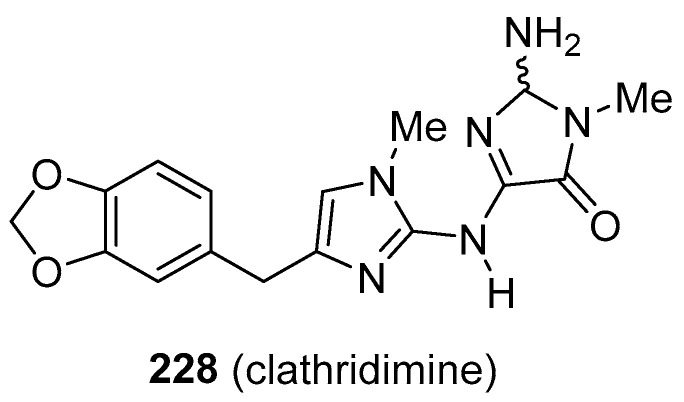
Structure of clathridimine (**228**).

In 2010, Bourguet-Kondracki and coworkers isolated 2-aminoimidazole alkaloid clathridimine (**228**) (Figure 10) from the Mediterranean calcareous sponge *Clathrina clathrus* and found that this metabolite exhibited selective anti-*E. coli* activity and that its zinc complex showed anti-*S. aureus* activity [142]. Unfortunately, this alkaloid has not yet been synthesised.

**Scheme 42:**
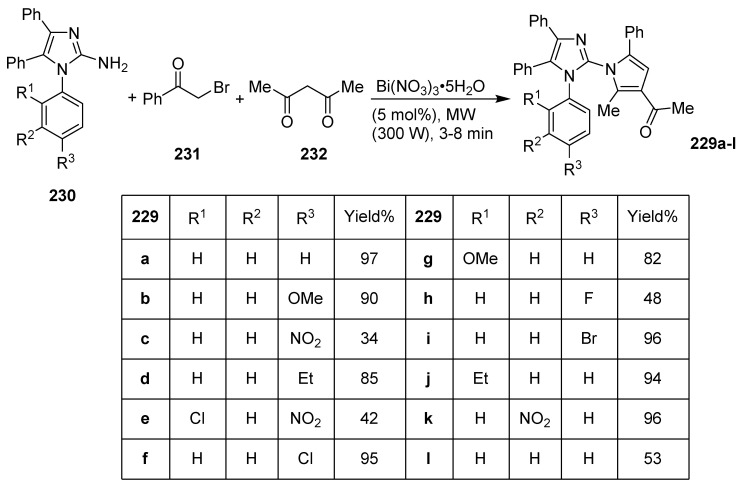
Synthesis of 1-[1-(1-aryl-4,5-diphenyl-1*H*-imidazol-2-yl)-2-methyl-5-phenyl-1*H*-pyrrol-3-yl]ethan-1-ones **229a**–**l.**

Recently, unnatural pyrrole/imidazole hybrids have also been the subject of synthetic and biological studies. In 2018, Chawla and Kapoor [143] synthesised 1-[1-(1-aryl-4,5-diphenyl-1*H*-imidazol-2-yl)-2-methyl-5-phenyl-1*H*-pyrrol-3-yl]ethan-1-ones **229a**–**l** in moderate to excellent yields by microwave-promoted Bi(NO_3_)_3_·5H_2_O-catalysed reaction of the appropriate 1-aryl-4,5-diphenyl-1*H*-imidazole-2-amines **230** [144] with phenacyl bromide (**231**) and 2,4-pentandione (**232**) (Scheme 42).

Hybrids **229** were next evaluated for their antibacterial activities against *S. aureus* MTCC 96, *B. subtilis* MTCC 121, *E. coli* MTCC 614, and *P. aeruginosa* MTCC 2453 using ciprofloxacin as standard drug, and it was found that compounds **229c**, **229h**, **229k**, and **229l** revealed significant activity that was better against the Gram-positive bacteria rather than the Gram-negative bacteria [143]. The zones of inhibition of **229c**, **229h**, and **229l** at 100 μg/mL towards *S. aureus* MTCC 96 were 24, 25, and 24 mm, respectively, while the zones of inhibition of these same compounds towards *P. aeruginosa* MTCC 2453 were 17, 19, and 20 mm, respectively [143].

**Scheme 43:**
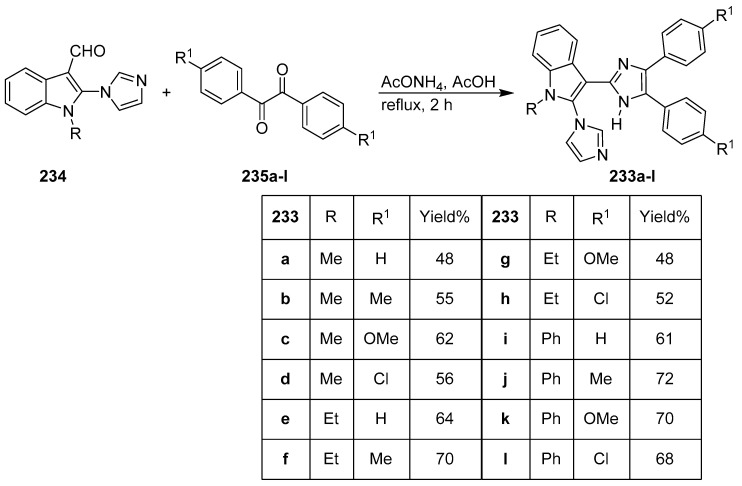
Synthesis of 1-substituted 3-(4,5-diaryl-1*H*-imidazol-2-yl)-2-(1*H*-imidazol-1-yl)-1*H*-indoles **233a**–**l.**

In 2004, a study on the synthesis and antimicrobial activities of some imidazole/indole hybrids was carried out by Benkli and coworkers [145]. These hybrids included 1-substituted 3-(4,5-diaryl-1*H*-imidazol-2-yl)-2-(1*H*-imidazol-1-yl)-1*H*-indoles **233a**–**l**, which were prepared in 48–72% yield by treatment of equimolar amounts of 1-substituted 2-(1*H*-imidazol-1-yl)-1*H*-indole-3-carbaldehydes **234** with the appropriate benzil derivatives **235** and AcONH_4_ in AcOH under reflux for 2 h (Scheme 43).

Among the twelve synthesised hybrids, the most significant antibacterial activity was exhibited by compound **233j**, which was prepared in 72% yield and had an MIC value (63 μM) against *E. coli* that was comparable to that of the control antibiotic chloramphenicol succinate (72 μM) [145].

**Scheme 44:**
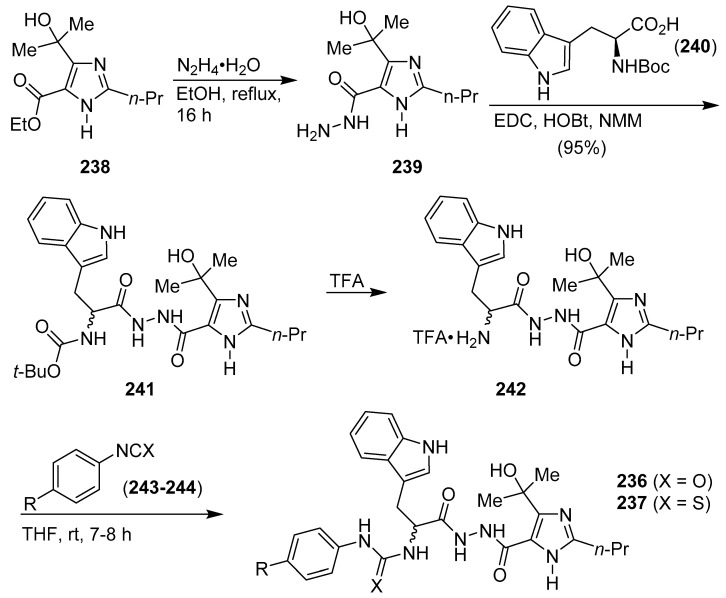
Synthesis of imidazole/indole hybrids **236** and **237.**

More recently, Channe Gowda and coworkers synthesised imidazole/indole hybrids **236** and **237** via the multi-step route outlined in Scheme 44 [146].

Imidazole **238** was treated with a molar excess of hydrazine hydrate to give compound **239**, which was treated with *N*-protected tryptophan **240**, EDC, and HOBt using 4-methylmorpholine (NMM) as a base to provide compound **241**. This intermediate was treated with TFA and the resulting compound **242** was treated with the required aryl isocyanates **243** or aryl isothiocyanates **244** providing ureas **236** and thioureas **237**, respectively. These imidazole/tryptophan hybrids were tested for their antibacterial properties against *E. coli* and *S. aureus* using streptomycin as the reference drug. The obtained results showed that hybrids **236a** (R = 4-FC_6_H_4_), **236b** (R = 4-(NO_2_)C_6_H_4_), **237a** (R = 4-FC_6_H_4_), and **237b** (R = 4-(NO_2_)C_6_H_4_) exhibited inhibitory activity higher than that of streptomycin and that urea derivatives **236** were more active than thiourea derivatives **237**. It was also observed that the electronic properties of hybrids **236** and **237** had a close relationship with the biological properties and that hybrids **236** and **237** bearing phenyl groups 4-substituted by electron-withdrawing groups (NO_2_ > F > Cl > OMe > Me) had antimicrobial activity higher than that of the reference drug [146].

**Scheme 45:**
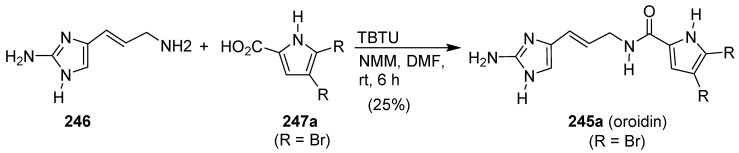
Synthesis of oroidin (**245a**) from (*E*)-4-(3-aminoprop-1-en-1-yl)imidazole)-2-amine (**246**) and 4,5-dibromo-1*H*-pyrrole-2-carboxylic acid (**247a**).

Over the past two decades, much attention was also paid to the synthesis and evaluation of the antimicrobial properties of oroidin [(*E*)-*N*-[3-(2-amino-1*H*-imidazol-4-yl)allyl]-4,5-dibromo-1*H*-pyrrole-2-carboxamide] (**245a**) [147,148,149,150,151,152], a naturally occurring pyrrole–2-aminoimidazole alkaloid, which was originally isolated from sponges of the genus *Agelas* [153,154,155,156]. A recent synthesis of **245a** involved the reaction of (*E*)-4-(3-aminoprop-1-en-1-yl)imidazole-2-amine (**246**) with 4,5-dibromo-1*H*-pyrrole-2-carboxylic acid (**247a**), the coupling reagent *N*,*N*,*N*′,*N*′-tetramethyl-*O*-(benzotriazol-1-yl)uronium tetrafluoroborate (TBTU) in NMM, and DMF at room temperature for 6 h (Scheme 45) [152]. This reaction produced naturally occurring hybrid **245a** in 25% yield [152].

Interestingly, naturally occurring hybrid **245a** at a concentration of 50 μM exhibited noticeably high activity against the Gram-positive bacteria *S. aureus* (>90% inhibition of growth) (IC_50_ = 31 µM) and *E. faecalis* (approximately 50% inhibition of growth), but was inactive against the Gram-negative bacterium *E. coli* [152].

**Figure 11:**
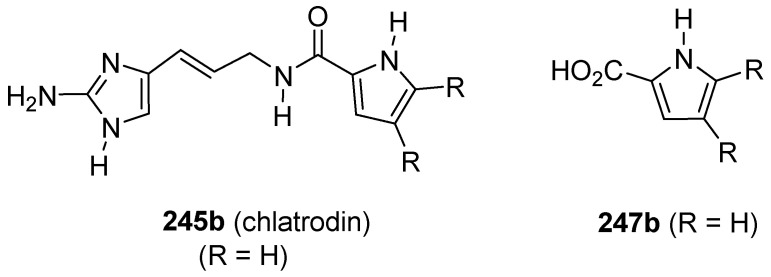
Structures of clathrodin (**245b**) and 1*H*-pyrrole-2-carboxylic acid (**247b**).

Tammela, Mašič, and coworkers also synthesised naturally occurring (*E*)-*N*-[3-(2-amino-1*H*-imidazol-4-yl)allyl]-1*H*-pyrrole-2-carboxamide (clathrodin) (**245b**) (Figure 11) in 18% yield by TBTU-mediated reaction of **246** with 1*H*-pyrrole-2-carboxylic acid (**247b**) in NMM and DMF at room temperature [152]. They also found that **245b** exhibited activity below the hit threshold (>80% inhibition of growth at a concentration of 50 μM) against all the microbial strains tested [152].

## 8. Pyrazole/Imidazole Hybrids

Over the past two decades, research has also been conducted on pyrazole-based compounds possessing significant anti-microbial activity [157,158,159,160]. Some of these studies focused in particular on the synthesis and evaluation of the in vitro activity of imidazole/pyrazole hybrids.

**Scheme 46:**
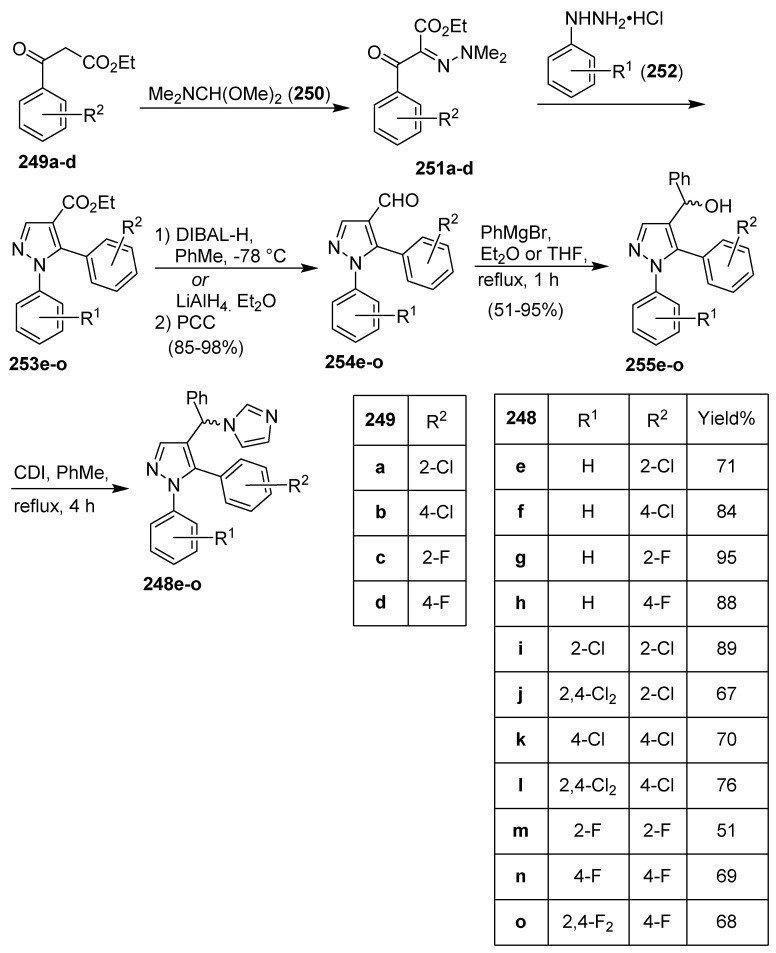
Synthesis of 1,5-disubstituted 4-[(1*H*-imidazol-1-yl)(phenyl)methyl]-1*H*-pyrazoles **248e**–**o.**

**Scheme 47:**
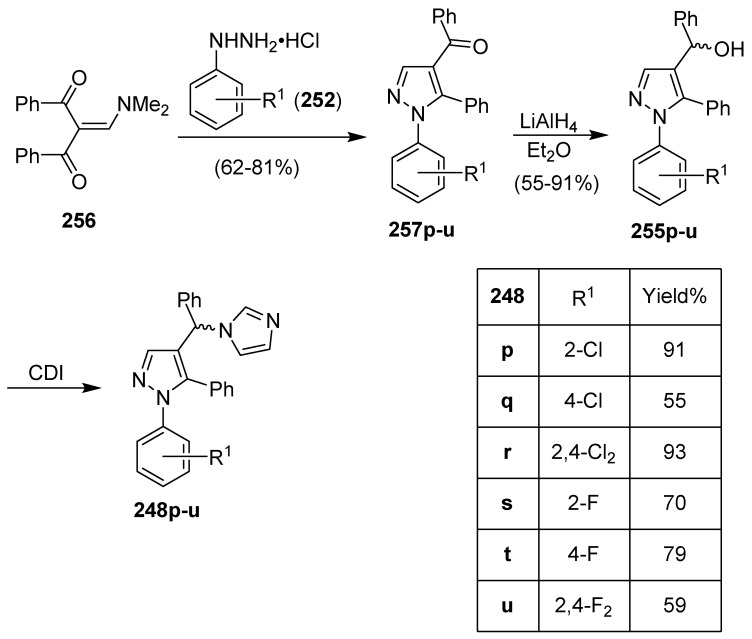
Synthesis of 1,5-disubstituted 4-[(1*H*-imidazol-1-yl)(phenyl)methyl]-1*H*-pyrazoles **248p**–**u.**

In 2004, Menozzi, La Colla, and coworkers synthesised 1,5-disubstituted 4-[(1*H*-imidazol-1-yl)(phenyl)methyl]-1*H*-pyrazoles **248e**–**o** and **248p**–**u** via the routes depicted in Scheme 46 and Scheme 47, respectively [161]. In particular, ethyl aroylacetates **249a**–**d** were treated with *N*,*N*-dimethylformamide dimethyl acetal (**250**) and the resulting compounds **251a**–**d** were treated with the appropriate arylhydrazines **252** affording pyrazoles **253e**–**o**. The alcohols, which were obtained by reduction of these compounds with diisobutylaluminum hydride (DIBAL-H) in toluene or with LiAlH_4_ in Et_2_O, were oxidised with pyridinium chlorochromate (PCC) in CH_2_Cl_2_ and the resulting aldehydes **254e**–**o** were treated with phenylmagnesium bromide yielding alcohols **255e**–**o**. Finally, treatment of these intermediates with *N*,*N*’-carbonyldiimidazole (CDI) in dry toluene under reflux for 4 h provided hybrids **248e**–**o** in moderate to good yields (Scheme 46).

Hybrids **248p**–**u** (Scheme 47) were instead prepared by reaction of 2-[(dimethylamino)methylene]-1,3-diphenylpropane-1,3-dione (**256**) [161] with the appropriate arylhydrazine hydrochlorides **252** in refluxing EtOH. Reduction of the resulting ketones **257** with LiAlH_4_ in Et_2_O led to alcohols **255**, which by treatment with CDI provided hybrids **248** [161].

Hybrids **248p**–**u** were proven to be weakly efficacious against *S. aureus* whereas other tested hybrids showed no activity. Furthermore, all hybrids **248** turned out to be inactive towards Gram-negative *Salmonella* spp. In addition, hybrids **248p**–**u** were proven to be the most potent derivatives against *M. tuberculosis* strains resistant to drugs commonly employed in the antitubercular therapy, and their activity was superior to that of clotrimazole and econazole, which were used as reference drugs [161].

**Scheme 48:**
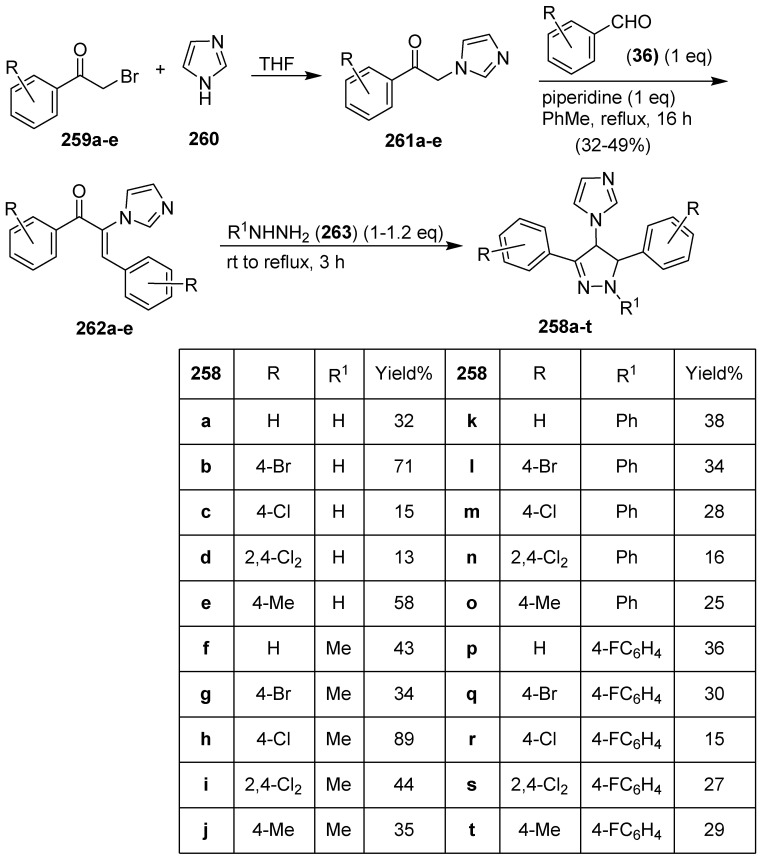
Synthesis of 4-(1*H*-imidazol-1-yl)-3,5-diaryl-4,5-dihydro-1*H*-pyrazole derivatives **258a**–**t.**

In 2008, Mamolo and coworkers described the synthesis of 4-(1*H*-imidazol-1-yl)-3,5-diaryl-4,5-dihydro-1*H*-pyrazole derivatives **258a**–**t** and tested the antitubercular activity of these hybrids against *M. tuberculosis* H_37_Rv [162]. The three-step synthesis of compounds **258** (Scheme 48) was carried out by treating substituted 2-bromoacetophenones **259** with imidazole (**260**) in THF providing 1-aryl-2-(1*H*-imidazol-1-yl)ethan-1-ones **261**. The subsequent condensation of these compounds with a series of substituted benzaldehydes **36** in refluxing toluene in the presence of piperidine, followed by treatment of the resulting α,β-unsaturated ketones **262** with EtOH solutions of hydrazines **263** (hydrazine hydrate, methylhydrazine sulfate, phenylhydrazine, and 4-fluorophenylhydrazine), produced hybrids **258a**–**t** in yields ranging from 15 to 89%.

All compounds **258** were evaluated for antitubercular activity against *M. tuberculosis* H_37_Rv using a micro-dilution Resazurin assay [163], and it was found that they showed good antimycobacterial activity, which in the case of hybrids **258m**, **258n**, **258o**, **258q**, **258r**, and **258t** reached MIC values of 7–10 μM [162].

**Scheme 49:**
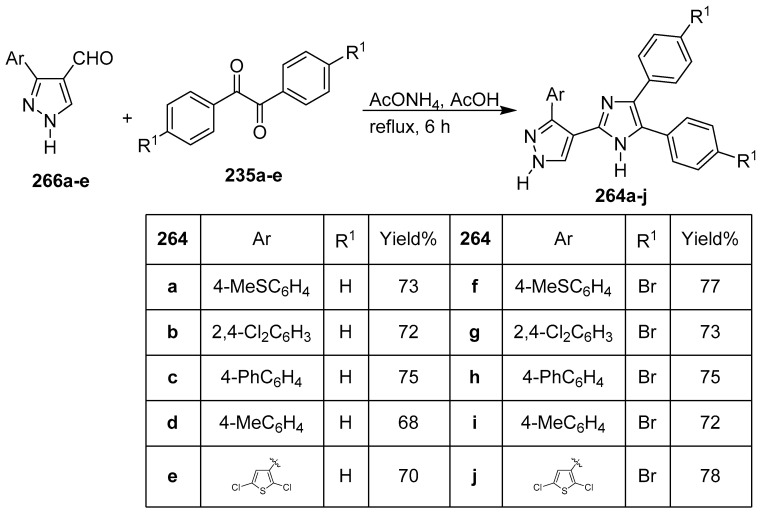
Synthesis of 3-aryl-4-(4,5-diaryl-1*H*-imidazol-2-yl)-1*H*-pyrazoles **264a**–**j.**

In 2011, Isloor and coworkers synthesised and characterised 3-aryl-4-(4,5-diaryl-1*H*-imidazol-2-yl)-1*H*-pyrazoles **264a**–**j** and methyl (*Z*)-2-(3-{[(*E*)-(3-aryl-1*H*-pyrazol-4-yl)] methylene}amino)-5-oxo-2-sulfanylideneimidazolidin-4-ylidene]acetates **265a**–**d** [164]. Hybrids **264** were synthesised in good yields by refluxing 3-aryl-1*H*-pyrazole-4-carbaldehydes 266 (1 eq) with 1,2-diketones 235 (1 eq) and AcONH_4_ (5 eq) in AcOH for 6–7 h (Scheme 49).

**Scheme 50:**
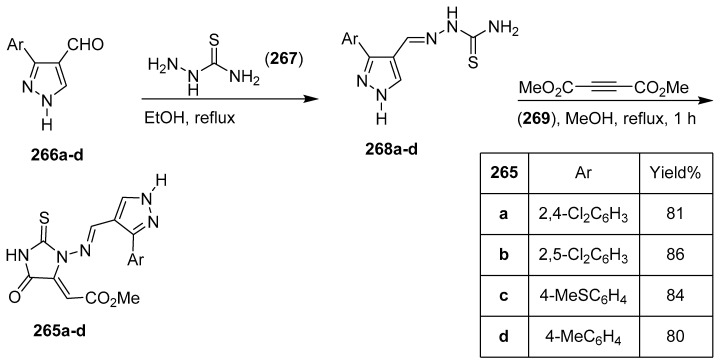
Synthesis of hybrids **265a**–**d.**

Hybrids **265** were instead prepared in good yields by reaction of aldehydes **266** with thiosemicarbazide (**267**) followed by treatment of the resulting thiosemicarbazones **268** with equimolar amounts of dimethyl acetylenedicarboxylate (**269**) in MeOH under reflux for 1 h (Scheme 50) [164].

The results of screening tests of the antibacterial properties of hybrids **264** and **265** against *E. coli*, *S. aureus*, *B. subtilis*, *Salmonella* Typhimurium (namely, *S. enterica* subsp. *enterica* sv. Typhimurium), *Clostridium perfringens*, and *P. aeruginosa* showed that methyl (*Z*)-2-{3-[({(*E*)-[3-(4-methylsulfanyl)phenyl]-1*H*-pyrazol-4-yl}]methylene)amino]-5-oxo-2-sulfanylideneimidazolidin-4-ylidene}acetate (**265c**) exhibited excellent activity against *P. aeruginosa* and *C. perfringens* at concentrations of 2.5 and 1.2 µM, respectively, compared to the standard drug streptomycin [164]. Instead, the remaining hybrids were found to possess moderately good activity against all six bacterial strains tested [164].

**Scheme 51:**
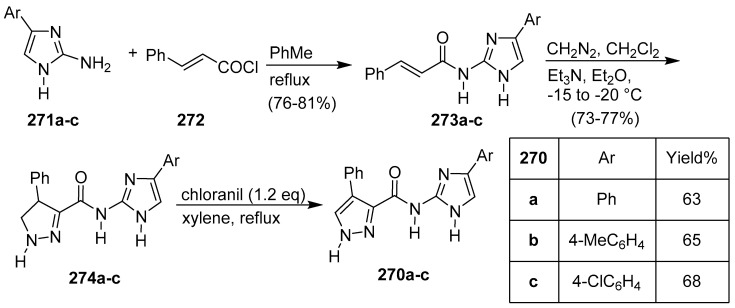
Synthesis of *N*-(4-aryl-1*H*-imidazol-2-yl)-4-phenyl-1*H*-pyrazole-3-carboxamides **270a**–**c.**

Still in 2011, Padmavathi and coworkers prepared *N*-(4-aryl-1*H*-imidazol-2-yl)-4-phenyl-1*H*-pyrazole-3-carboxamides **270a**–**c** via the three-step route shown in Scheme 51, which involved the use of 4-aryl-1*H*-imidazol-2-amines **271** as the starting material [165].

Treatment of amines **271a**–**c** with cinnamoyl chloride (**272**) in toluene under reflux gave (*E*)-*N*-(4-aryl-1*H*-imidazol-2-yl)cinnamamides **273a**–**c** in 76–81% yield. Subsequently, CH_2_Cl_2_ solutions of these compounds were treated with an Et_2_O solution of diazomethane at −15 to −20 °C in the presence of Et_3_N for 42–48 h affording compounds **274a**–**c** in 73–77% yield. Finally, these compounds were converted to the required hybrids **270a**–**c** in 63–68% yield by treatment with 1.2 eq of chloranil in xylene under reflux for 24–25 h [165].

Padmavathi and coworkers then found that, when compared to the standard drug chloramphenicol, hybrid **270c** at a concentration of 270 μM displayed significant antibacterial activity and its inhibition zones against *S. aureus*, *B. subtilis*, *P. aeruginosa*, and *K. pneumoniae* were 34, 35, 32, and 39 mm, respectively. Instead, the inhibition zones of chloramphenicol at 310 μM against these bacterial species were 35, 38, 30, and 42 mm, respectively [165].

**Scheme 52:**
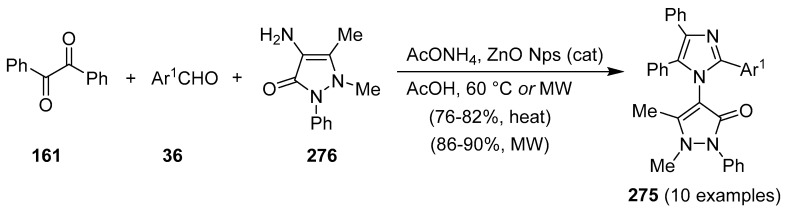
Synthesis of 4-(2-aryl-4,5-diphenyl-1*H*-imidazol-1-yl)-1,5-dimethyl-2-phenyl-1,2-dihydro-3*H*-pyrazol-3-ones **275.**

More recently, Narayana and coworkers synthesised ten 4-(2-aryl-4,5-diphenyl-1*H*-imidazol-1-yl)-1,5-dimethyl-2-phenyl-1,2-dihydro-3*H*-pyrazol-3-ones **275** via atom-economic, one-pot reaction of equimolar amounts of benzil (**161**), aryl aldehydes **36**, commercially available 4-aminoantipyrine (**276**), and AcONH_4_ in glacial acetic acid, in the presence of a catalytic amount of ZnO nanoparticles (ZnO Nps) (Scheme 52) [166]. The preparation of this catalyst, which could be reused even after four catalytic cycles, was performed according to a documented procedure [166]. The cyclocondensation reaction, when carried out at 60 °C, provided hybrids **275** in 76–82% yield, but higher yields (86–90%) were obtained by carrying out the cyclocondensation reaction under microwave irradiation using a domestic microwave oven with a continuous irradiation power.

All hybrids **275** were evaluated for their antibacterial activities against *S. aureus* and *E. coli* by resazurin reduction assay and demonstrated significant activity. The IC_50_ values of compounds **275c** (Ar^1^ = 4-FC_6_H_4_) and **275g** (Ar^1^ = 4-MeOC_6_H_4_), which were found to be the most active among the tested hybrids, were 46 and 53 μM, respectively, for *S. aureus*, and 88 and 23 μM, respectively, for *E. coli* [166].

## 9. 1,2,3- and 1,2,4-Triazole/Imidazole Hybrids and Conjugates

Over the last few decades, numerous 1,2,3-triazole-containing hybrids and conjugates possessing antibacterial activity have been identified [167,168,169,170,171,172,173,174] and some of them have been cited and commented on in an interesting review published in 2019 by Aisa and coworkers [174]. Unfortunately, this review did not include the synthesis and antibacterial properties of 1,2,3-triazole/imidazole hybrids and conjugates. Nevertheless, still in 2019, Gao, Wang, Xiao, and Huang summarised recent advances on the promising antibacterial activities of 1,2,4-triazole hybrids against both drug-sensitive and drug-resistant pathogens [175].

In this section of the review, we have summarised and commented the data on antibacterial 1,2,3- and 1,2,4-triazole/imidazole hybrids and conjugates, which were described in the literature from 2015 until the end of February 2020, noting that few of them [32,59] have already been discussed in Section 1 of the present review.

**Scheme 53:**
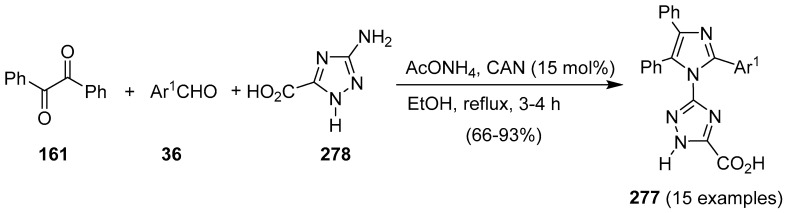
Synthesis of 3-(2-aryl-4,5-diphenyl-1*H*-imidazol-1-yl)-1*H*-1,2,4-triazole-5-carboxylic acid derivatives **277.**

In 2015, Nikalje and coworkers synthesised 3-(2-aryl-4,5-diphenyl-1*H*-imidazol-1-yl)-1*H*-1,2,4-triazole-5-carboxylic acid derivatives **277** and evaluated the in vitro antibacterial activities against *E. coli*, *S. aureus*, *S. pneumoniae*, and *S. pyogenes* using ampicillin as a reference drug [176]. As shown in Scheme 53, compounds **277** were synthesised in 66–93% yield by CAN catalysed the four-component cyclocondensation reaction of benzil (**161**), aryl aldehydes **36**, commercially available 3-amino-1*H*-1,2,4-triazole-5-carboxylic acid (**278**), and AcONH_4_ in EtOH under reflux for 3–4 h.

The results of the in vitro antibacterial data of compounds **277** showed that all these substances possessed moderate activity (MIC range = 130–1200 μM) when compared with ampicillin (MIC range = 290–720 μM). It was specifically found that **277e** (Ar^1^ = 4-ClC_6_H_4_) showed higher activity (MIC = 140 μM) against *E. coli*, **277b** (Ar^1^ = Ph) (MIC = 150 μM) against *S. pneumoniae*, and **277n** (Ar^1^ = 2,4-(MeO)_2_C_6_H_3_) (MIC = 130 μM) against *S. pyogenes* than ampicillin (290 μM). Furthermore, against *S. aureus*, **277n** was the most active among the tested compounds (MIC = 210 μM) compared to ampicillin (720 μM) [176].

**Scheme 54:**
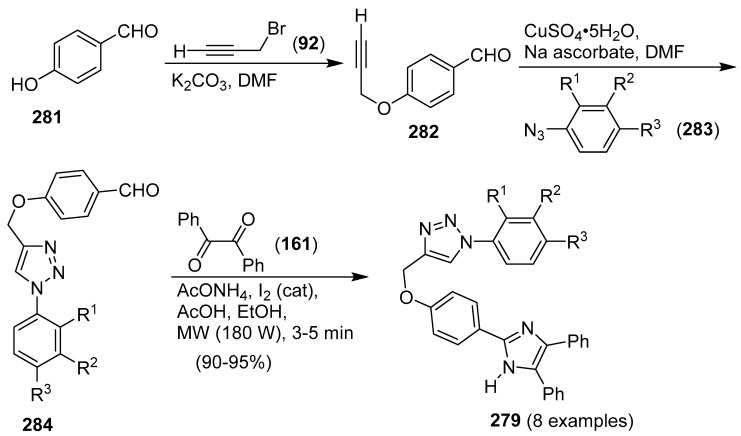
Synthesis of imidazole/1,2,3-triazole hybrids **279.**

In 2019, Subhashini and coworkers synthesised eight imidazole-linked mono-1,2,3-triazoles **279** and eight imidazole-linked bis-1,2,3-triazoles **280** and tested the antibacterial properties of these hybrids against *S. aureus*, *B. cereus*, *E. coli*, *P. vulgaris*, and the fungal species *Aspergillus fumigatus* using ampicillin as a reference drug [177]. Hybrids **279** were synthesised via the three-step procedure shown in Scheme 54 that involved the reaction of 4-hydroxybenzaldehyde (**281**) with propargyl bromide (**92**) in DMF in the presence of K_2_CO_3_.

The Cu(I)-catalysed click reaction between the resulting 1-alkyne **282** and aryl azides **283** in DMF in the presence of sodium ascorbate as a reduction reagent gave rise to 4-[(1-aryl-1*H*-1,2,3-triazol-4-yl)methoxy]benzaldehydes **284**. Finally, the microwave-mediated reaction of these derivatives (1 eq) with benzil (**161**) (1 eq) and AcONH_4_ (4 eq) in EtOH in the presence of a catalytic amount of iodine provided hybrids **279** in yields ranging from 90 to 95% (Scheme 54). It was also observed that when this multicomponent reaction was carried out using conventional heat sources at 80 °C for 4–5 h hybrids **279** were obtained in yields ranging from 60 to 64% [177].

**Scheme 55:**
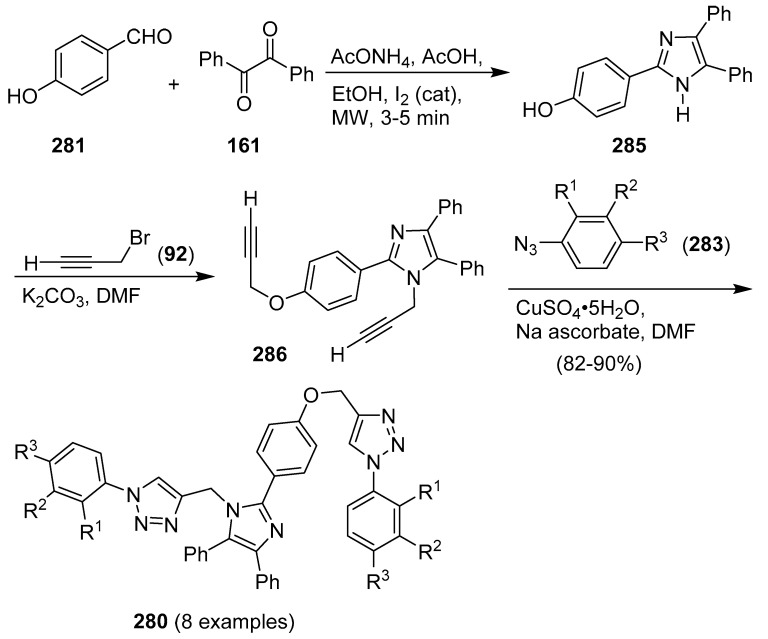
Synthesis of imidazole/1,2,3-triazole hybrids **280.**

Imidazole-linked bis-1,2,3-triazoles derivatives **280** were instead prepared via the route depicted in Scheme 55, in which the order of the sequence of reactions that were used for the synthesis of hybrids **284** was reversed.

In particular, 4-hydroxybenzaldehyde (**281**) was treated with benzil (**161**) and AcONH_4_ in AcOH in the presence of a catalytic amount of iodine under microwave irradiation at 180 W for 3–5 min, affording trisubstituted imidazole **285**. Bis-propargylation of **285** with propargyl bromide (**92**) in DMF in the presence of K_2_CO_3_ gave compound **286**, which underwent microwave-promoted Cu(I)-catalysed click reaction with azides **283** in DMF, providing hybrids **280** in good to excellent yields [177].

Hybrids **279** and **280** were next characterised and tested for their antibacterial activity and it was found that compounds **279c** (R^1^ = R = H; R^3^ = Me), **279h** (R^1^ = R^2^ = H; R^3^ = NO_2_), **280d** (R^1^ = OMe; R^2^ = R^3^ = H), and **280e** (R^1^ = OMe; R^2^ = H; R^3^ = NO_2_) were capable to inhibit the bacterial growth more effectively than other hybrids with MIC values ranging from 11 to 69 μM. For example, the MIC values of **280e** against *E. coli* and *B. cereus* were 11 and 12 μM, respectively, whereas the MIC values of ampicillin against these two bacterial strains were 31 and 24 μM, respectively. Furthermore, the MIC values of **279h** against *S. aureus*, *B. cereus*, *E. coli*, and *P. vulgaris* were 30, 26, 19, and 46 μM, respectively [177].

**Scheme 56:**
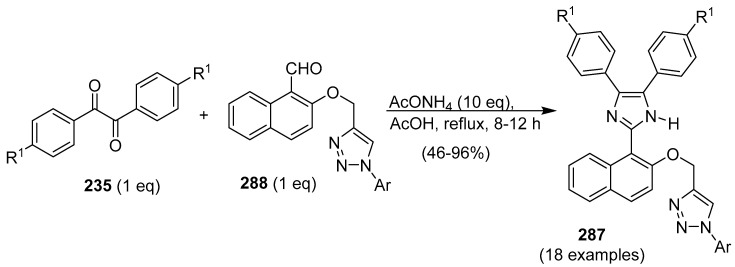
Synthesis of 2,4,5-trisubstituted 1*H*-imidazole/1,2,3-triazole hybrids **287.**

Still in 2019, Verma and coworkers synthesised in moderate to excellent yields 4-{[1-(4,5-diaryl-1*H*-imidazol-2-yl)naphthalen-2-yl)oxy]methyl}-1-substituted-1*H*-1,2,3-triazoles **287** by condensation reaction of α-diketones **235** with an equimolar amount of 1,2,3-triazole -substituted naphthaldehydes **288** and a large molar excess of AcONH_4_ in glacial acetic acid under reflux for 8–12 h (Scheme 56) [178].

The evaluation of hybrids **287** for their in vitro antibacterial activities against *B. subtilis*, *S. epidermidis*, *E. coli*, and *P. aeruginosa* using ciprofloxacin as a positive control showed that compound **287h** (R^1^ = H; Ar = 4-BrC_6_H_4_) was the most active against *S. epidermidis* (MIC = 5.2 μM) and that compound **287e** (R^1^ = H; Ar = 2-NO_2_C_6_H_4_) was highly active against *E. coli* (MIC = 5.5 μM). The MIC value of ciprofloxacin against all four bacterial strains tested was 4.7 μM. It was also found that hybrid **287e** was anchored in the binding site of DNA gyrase topoisomerase II of *E. coli* [178].

**Scheme 57:**
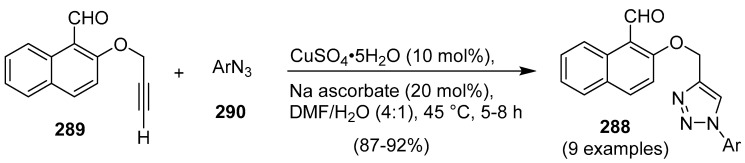
Synthesis of 1,2,3-triazole substituted naphthaldehydes **288.**

Nine 1,2,3-triazole-substituted naphthaldehydes **288** were in turn prepared in 87–92% yield by treating 2-(prop-2-yn-1-yloxy)-1-naphthaldehyde (**289**) with the appropriate aryl azides **290**, 10 mol% CuSO_4_·5H_2_O, and 20 mol% sodium ascorbate in a 4:1 mixture of DMF and water at 45 °C for 5–8 h (Scheme 57) [178].

## 10. Hybrids of Imidazole Derivatives and 5-Membered Heterocycles Containing Oxygen and Nitrogen

**Scheme 58:**
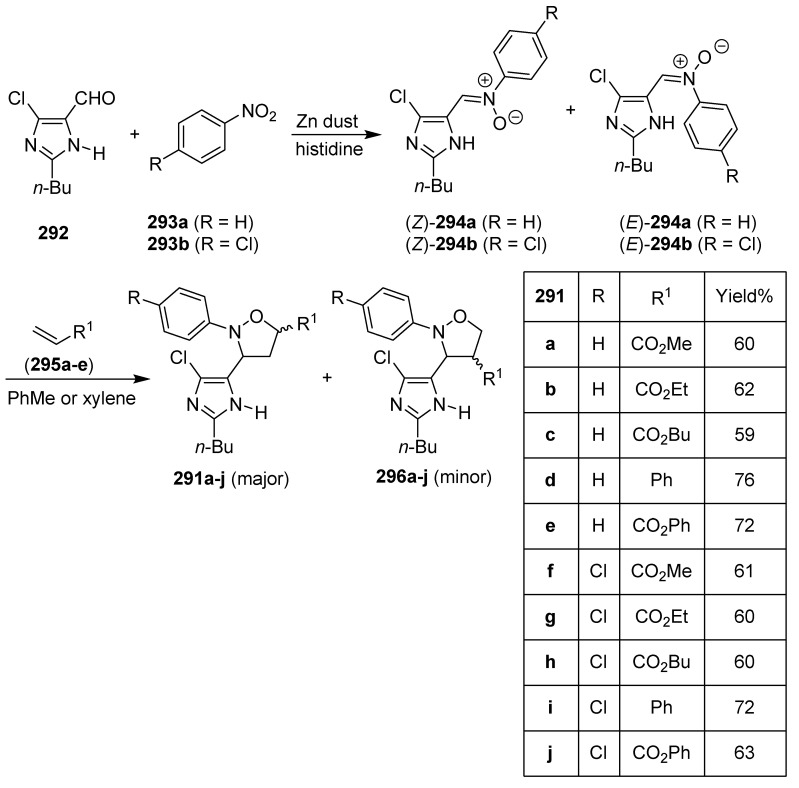
Synthesis of isoxazolidine/imidazole hybrids **291a**–**j.**

For several years, studies have been carried out to identify isoxazolidine derivatives with significant antibacterial properties [179,180,181,182]. In 2004, Rangappa and coworkers synthesised with high regioselectivity isoxazolidine/imidazole hybrids **291a**–**j** and tested their antibacterial activities against *S. aureus*, *E. coli*, and *B. subtilis* by using streptomycin as a positive control [183]. The synthesis of these hybrids (Scheme 58) was accomplished by treatment of 2-butyl-4(5)-chloro-1*H*-imidazole-5(4)-carbaldehyde (**292**) [184,185], a compound currently commercially available, with nitro compounds **293a** and **293b** and zinc dust, using histidine as catalyst, according to a previously reported procedure [186]. The resulting nitrones (*Z*)-**294a** and (*Z*)-**294b** (that were incorrectly reported as (*E*)-stereoisomers), which were contaminated by the corresponding (*E*)-stereoisomers, (*E*)-**294a** and (*E*)-**294b**, respectively, were subjected to 1,3-dipolar cycloaddition reaction with a molar excess of 1-alkenes **295** in a toluene or xylene solution giving rise to hybrids **291a**–**j** in yields ranging from 59 to 72% together with minor amounts of the corresponding regioisomers **296a**–**j**.

The results of the evaluation of the antibacterial activity of hybrids **291a**–**j** showed that compounds **291c**, **291d**, **291h**, and **291i** exhibited about 50–60% inhibition when compared with streptomycin and that the remaining hybrids were not effective against *S. aureus*, *E. coli*, and *B. subtilis*. For example, the zone of inhibition of *S. aureus* for compound **291c** at a concentration of 25 mM was 21 mm, while that for streptomycin was 35 mm [183].

**Scheme 59:**
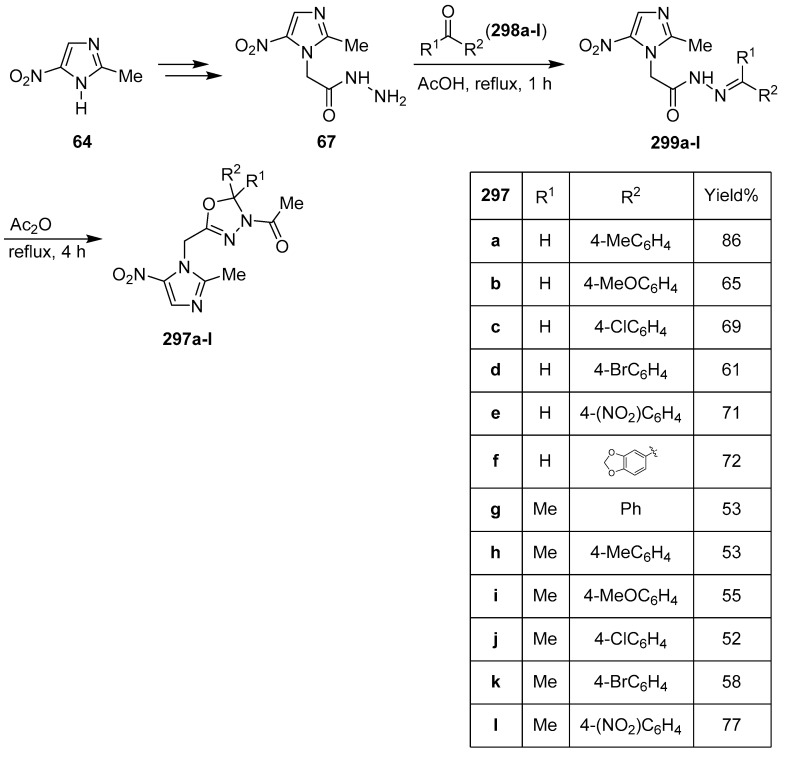
Synthesis of 1,3,4-oxadiazole/imidazole hybrids **297a**–**l.**

In 2005, Frank and Kalluraya synthesised 1,3,4-oxadiazole/imidazole hybrids **297a**–**l** in good yields by reaction of 2-(2-methyl-5-nitro-1*H*-imidazol-1-yl)acetohydrazide (**67**) with the appropriate ketones **298a**–**l** in glacial AcOH under reflux for 1 h, followed by treatment of the resulting compounds **299a**–**l** with a large molar excess of Ac_2_O under reflux for 4 h (Scheme 59) [187]. Compound **67** was in turn prepared in 67% yield by a two-step reaction sequence, in which 2-methyl-5-nitro-1*H*-imidazole (**64**) was the starting material (Scheme 59, cf. Scheme 12).

Hybrids **297a**–**l** when screened for their antibacterial activities against *S. aureus*, *P. aeruginosa*, *E. coli*, and *B. subtilis* using nitrofurazone as the standard drug turned out to display significant activity. For example, hybrid **297l** had an MIC value of 640 nM for each of these bacterial strains, while nitrofurazone had an MIC value of 2.5 μM for *S. aureus*, *P. aeruginosa*, and *B. subtilis* and of 1.3 μM for *E. coli* [187].

**Scheme 60:**
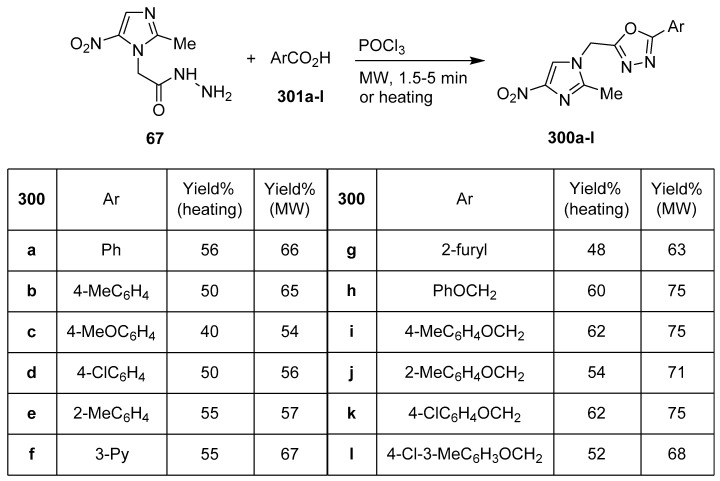
Synthesis of 2-aryl-5-[(2-methyl-4-nitro-1*H*-imidazol-1-yl)methyl]-1,3,4-oxadiazoles **300a**–**l.**

In 2007, Kalluraya and coworkers synthesised 2-aryl-5-[(2-methyl-4-nitro-1*H*-imidazol-1-yl)methyl]-1,3,4-oxadiazoles **300a**–**l** in moderate yields by treatment of hydrazide **67** with the appropriate arenecarboxylic acids **301a**–**l** in the presence of POCl_3_ under microwave irradiation or using the conventional heating method in an oil bath (Scheme 60) [188]. Higher yields of hybrids **300** (54–75%) were obtained when the reaction was carried out using microwave irradiation.

The antibacterial activities of hybrids **300a**–**l** was then evaluated at 10 mg/mL concentration against *S. aureus*, *K. pneumoniae*, *E. coli*, and *P. aeruginosa* using ciprofloxacin as a positive standard. The obtained results indicated that compounds **300e** and **300l** exhibited good activity against *K. pneumoniae*, *E. coli*, and *P. aeruginosa*, compound **300h** showed good activity against *E. coli* and *P. aeruginosa*, compound **300i** showed good activity against *P. aeruginosa* and *K. pneumoniae*, and compound **300j** showed good activity against *K. pneumoniae* [188]. For example, a diameter of zone inhibition of 28 mm was found for hybrids **300e** and **300i** against *P. aeruginosa*, while that of ciprofloxacin was 22 mm [188].

## 11. 1,8-Naphthalimide/Imidazole Hybrids

**Scheme 61:**
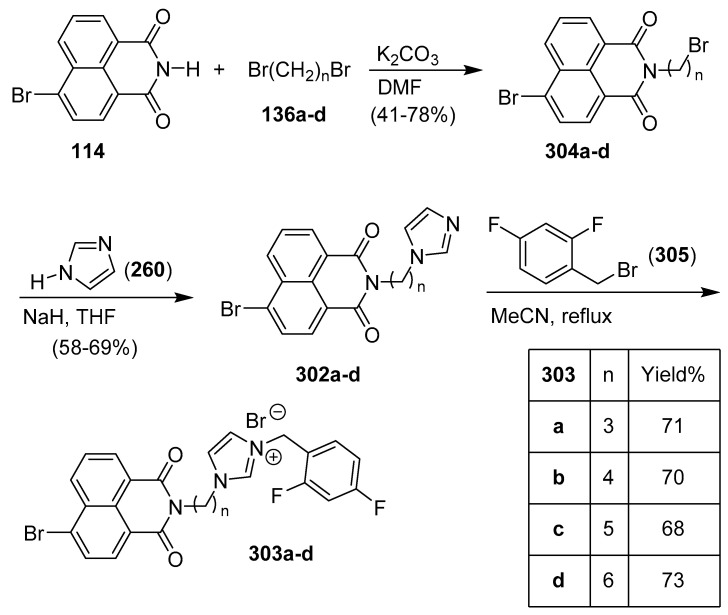
Synthesis of 1,8-naphthalimide-derived imidazoles **302a**–**d** and **303a**–**d.**

Several 1,8-naphthalimide derivatives are known as antibacterial agents [76,189,190,191]. In recent years, special attention has been paid to antibacterial 1,8-naphthalimide/imidazole hybrids [79,192,193]. In 2011, Zhang and Zhou synthesised 1,8-naphthalimide-derived imidazoles **302a**–**d** and **303a**–**d** via the route shown in Scheme 61 [76].

Specifically, compounds **304**, which were prepared in 41–78% yield by treatment of 4-bromo-1,8-naphthalimide (**114**) with a molar excess of α,ω-dibromoalkanes **136** in DMF in the presence of K_2_CO_3_, were treated with a mixture of imidazole (**260**) and NaH in THF providing hybrids **302a**–**d** in yields that ranged from 58 to 69%. Finally, the reaction of these compounds with 1-(bromomethyl)-2,4-difluorobenzene (**305**) in MeCN under reflux provided hybrids **303a**–**d** in good yields [76].

It deserves to be mentioned that in 2013 the synthesis of hybrids **302** and **303** via the route shown in Scheme 61 was repeated in detail by Lv, Zhou, and coworkers [77], and that in 2011 and in 2013, hybrids **302a**–**d** and **303a**–**d** were evaluated for their in vitro antimicrobial activities against Gram-positive bacteria *S. aureus* ATCC 25923, MRSA N315, *B. subtilis* ATCC 6633, *M. luteus* ATCC 4698, and Gram-negative bacteria *P. hauseri*, [60] *E. coli* JM 109, *P. aeruginosa*, and *Salmonella* Typhi (namely, *S. enterica* subsp. *enterica* sv. Typhi) [76,77,194]. Naphthalimide-based imidazoles **302a** and **302b** were found able to inhibit the growth of all bacterial strains tested with MIC values of 20–83 μM and hybrids **303a** and **303b** turned out to display better potency than other their analogues against the tested strains [76,77]. The MIC values of these two hybrids were 7 and 3 μM, respectively, against MRSA and this result suggested that they could be used as potential anti-MRSA agents in comparison with clinical drugs chloramphenicol [50] MIC = 12 μM) and orbifloxacin (MIC = 3 μM) [76]. Notably, compound **302b** with (CH_2_)_4_ linker exhibited efficacy against *E. coli* and *P. aeruginosa* with MIC values of 20 and 40 μM, respectively, which are comparable to that of the standard drug chloramphenicol (25 and 50 μM) [77].

In 2016, Cai, Zhou, and coworkers carried out the synthesis and biological evaluation of Schiff base-linked imidazolyl naphthalimides **306a**–**c**, **307**, **308a**–**e**, and **309a**–**k** and tested the in vitro antibacterial activities of these compounds against the Gram-positive bacteria MRSA, *S. aureus*, *B. subtilis*, and *M. luteus* and the Gram-negative bacteria *E. coli* DH52, *E. coli* JM109, *S. dysenteriae*, *P. aeruginosa*, *P. hauseri* [60], and *S.* Typhi [194,195].

**Scheme 62:**
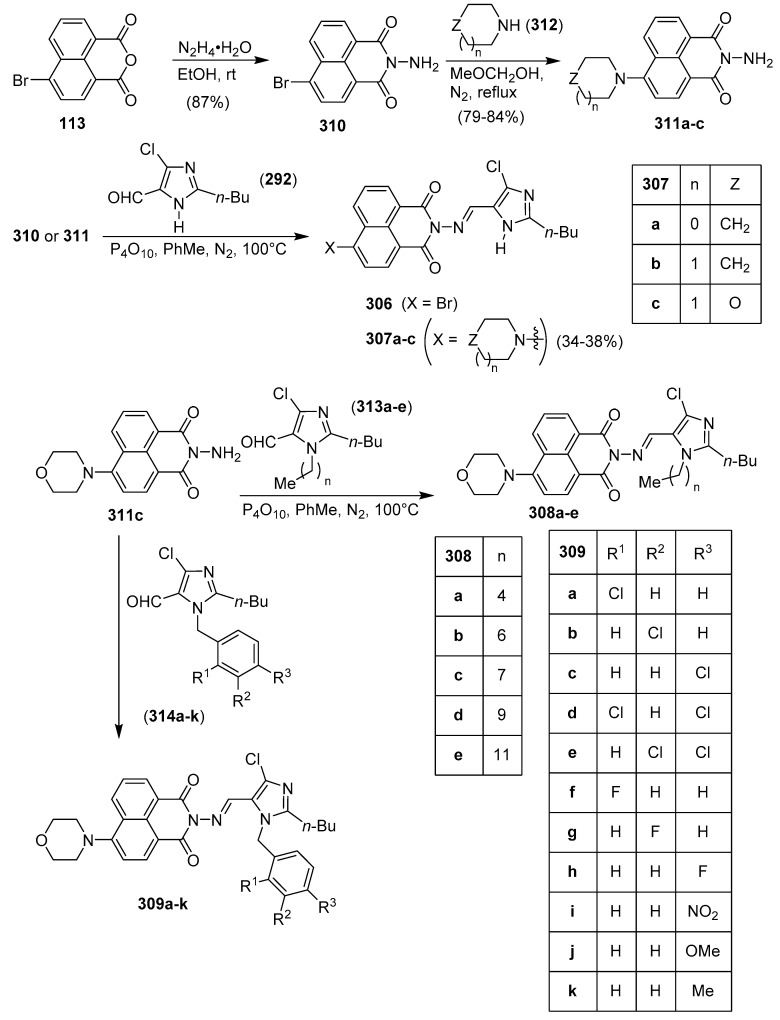
Synthesis of Schiff base-linked imidazolyl naphthalimides **306**, **307a**–**c**, **308a**–**e**, and **309a**–**k.**

The target hybrids were synthesised as outlined in Scheme 62. Compound **310**, which was obtained in 87% yield by reaction of anhydride **113** with hydrazine hydrate in EtOH at room temperature, was converted to naphthalimides **311a**–**c** in 79–84% yield by treatment with the appropriate alicyclic amines **312** in ethylene glycol monomethyl ether under reflux and N_2_ atmosphere. The subsequent condensation reaction of **311a**–**c** with 2-butyl-5-chloro-1*H*-imidazole-4-carbaldehyde (**292**) in toluene under reflux in the presence of 1 mol% P_4_O_10_ under N_2_ atmosphere provided hybrids **307a**–**c** in 34–8% yields. Compound **306** was instead obtained by the condensation reaction of **310** with **292** in an unspecified yield. Finally, target compounds **308** were synthesised in moderate yields by reaction of compound **311c** with the corresponding 1-alkylimidazolecarbaldehydes **313** and imidazolyl naphthalimides **309** were obtained by treatment of **311c** with the appropriate substituted 1-benzylimidazolecarbaldehydes **314** in toluene at 100 °C using P_4_O_10_ as dehydrating agent. Unfortunately, even in this case, the yields of the reactions were not reported [195].

All the above-mentioned target Schiff base-linked imidazolyl naphthalimides were tested for their in vitro antimicrobial activity against the Gram-positive bacteria MRSA, *S. aureus*, *B. subtilis*, and *M. luteus* and against the Gram-negative bacteria *E. coli* DH152, *E. coli* JM109, *S. dysenteriae*, *P. aeruginosa*, *P. hauseri* [60], and *S.* Typhi [194] using chloramphenicol [50] and norfloxacin as reference drugs. The results of preliminary screening tests showed that naphthalimides **307a**–**c** possessed antibacterial activity higher than that of compound **306**, and that **307c** exhibited the highest inhibition activity among these three compounds. It was also observed that the activity of hybrids **308a**–**c** was not much different from that of **307c**, whereas the antibacterial efficacies of hybrids **308d** and **308e** were better than that of compound **307c** against some tested strains. Interestingly, hybrid **308e**, which had an MIC value of 10 µM against *E. coli* JM109, was 10 times more potent than clinical drug chloramphenicol. It is also worth mentioning that: (i) hybrid **309b** proved capable of inhibiting the growth of B. subtilis at the concentration of 7 µM, was 14 times more potent than chloramphenicol and with activity comparable to that of norfloxacin; and (ii) hybrid **309i** had an MIC value of 3 µM against MRSA and was 17 and 7 times more potent than chloramphenicol and norfloxacin, respectively [195].

## 12. Bis-Imidazoles

Over the past few decades, considerable efforts have been directed toward the synthesis and evaluation of the biological properties of bis-imidazole derivatives, a class of heterocyclic compounds displaying significant levels of antifungal, antibacterial, and antimycobacterial activities [196,197,198,199,200,201,202,203].

**Scheme 63:**
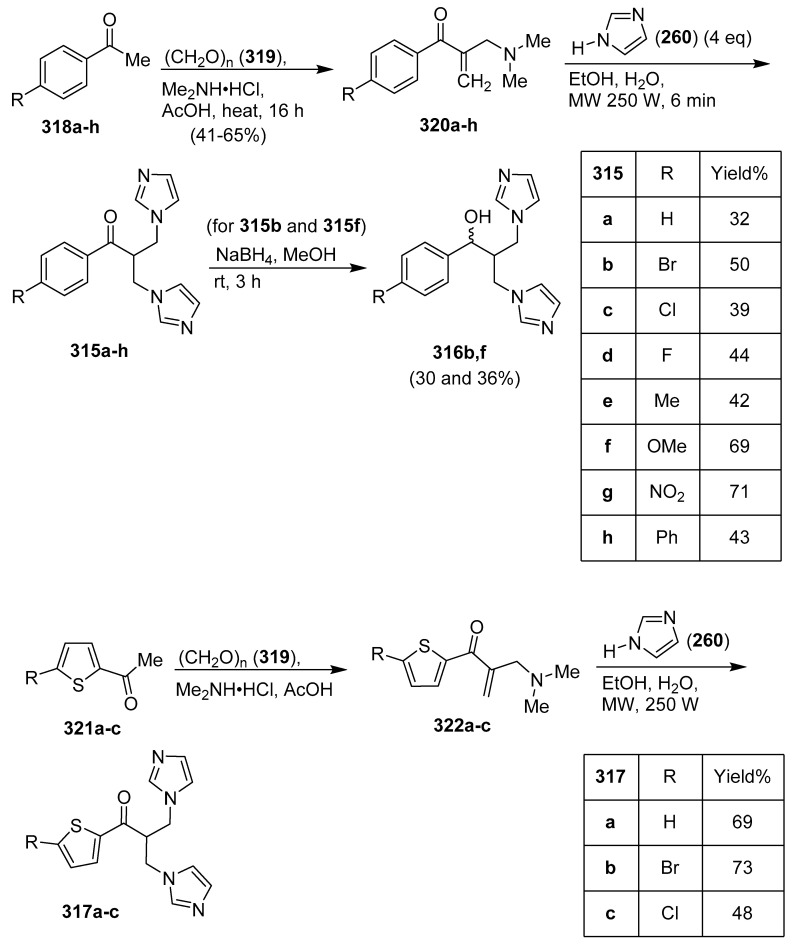
Synthesis of 1-aryl-3-(1*H*-imidazol-1-yl)-2-[(1*H*-imidazol-1-yl)methyl]propan-1-ones **315a**–**h**, 1-aryl-3-(1*H*-imidazol-1-yl)-2-[(1*H*-imidazol-1-yl)methyl]propan-1-ols **316b**,**f**, and 1-(5-substituted-thiophen-2-yl)-3-(1*H*-imidazol-1-yl)-2-[(1*H*-imidazol-1-yl)methyl]propan-1-ones **317a**–**c.**

In 2007, Mamolo and coworkers synthesised 1-aryl-3-(1*H*-imidazol-1-yl)-2-[(1*H*-imidazol-1-yl)methyl]propan-1-ones **315a**–**h**, 1-aryl-3-(1*H*-imidazol-1-yl)-2-[(1*H*-imidazol-1-yl)methyl] propan-1-ols **316b**,**f**, and 1-(5-substituted-thiophen-2-yl)-3-(1*H*-imidazol-1-yl)-2-[(1*H*-imidazol-1-yl)methyl]propan-1-ones **317a**–**c** via the reaction sequences shown in Scheme 63 [198]. Compounds **315** were prepared in 41–65% yield by the Mannich reaction of the appropriately 4-substituted acetophenones **318** with paraformaldehyde (**319**) and dimethylamine hydrochloride in AcOH. Subsequent irradiation of a water/EtOH solution of the resulting compounds **320a**–**h** with 4 eq of imidazole (**260**) at 250 W for 6 min in a domestic microwave oven provided bis-imidazoles **315a**–**h** in yields ranging from 32 to 71%. This reaction could also be carried out using conventional heating under reflux for 12–16 h, but the yields of the required bis-imidazoles were quite lower. Bis-imidazoles **316b** and **316f** were instead prepared in 30 and 36% yield, respectively, by treatment of **315b** and **315f** with NaBH_4_ in MeOH at room temperature for 3 h. Finally, bis-imidazole derivatives **317a**, **317b**, and **317c** were synthesised in 69, 73, and 48% yield, respectively, from the appropriate thienyl methyl ketones **321** using compounds **322a**–**c** as key intermediates [198].

Compounds **315a**–**h** and **316b**,**f** were then tested for their in vitro killing activities against *M. tuberculosis* H_37_Rv in comparison with antibiotic rifampicin and were found to exhibit a moderate activity with MIC values in the range 22–200 μM. It was particularly noted that the antimycobacterial activity of 1-[(1,1′-biphenyl)-4-yl]-3-(1*H*-imidazol-1-yl)-2-[(1*H*-imidazol-1-yl) methyl]propan-1-one (**315h**) was comparable to its antifungal activity towards clinical isolates of *Candida albicans* 3038 and *C. glabrata* 123, suggesting the presence of similar enzymatic interactions. Bis-imidazoles **317a**–**c** exhibited instead moderate antifungal activity against the strain of *C. albicans* 3038 [198].

**Scheme 64:**
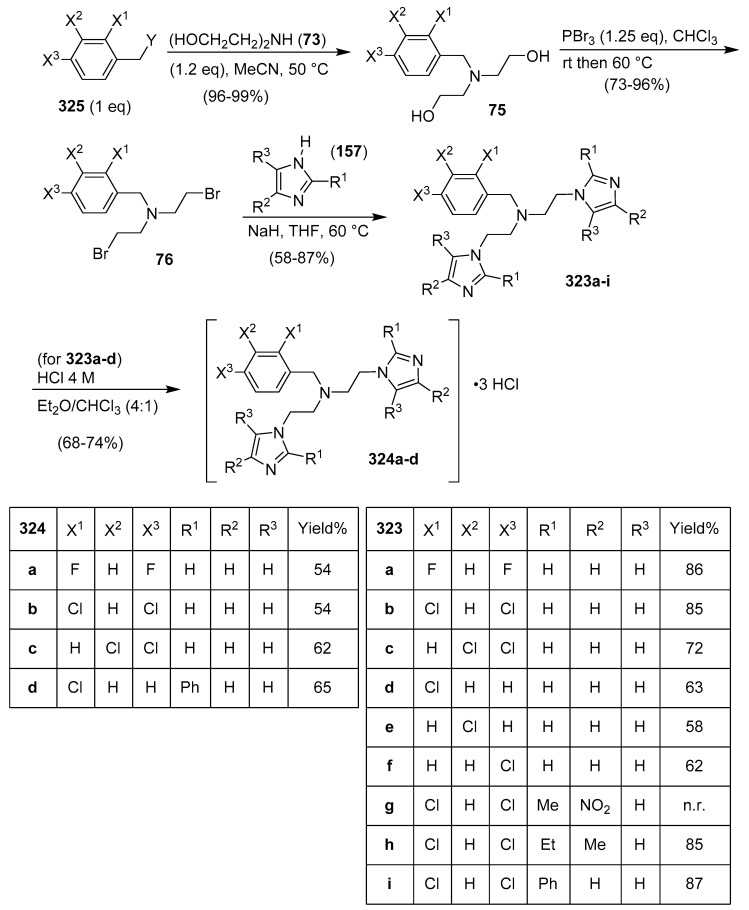
Synthesis of amine-derived bis-imidazoles **323a**–**i** and hydrochlorides **324a**–**d.**

In 2010, as part of a study concerning the evaluation of the bioactivity of structurally modified derivatives of fluconazole, Zhou and coworkers synthesised amine-derived bis-imidazoles **323a**–**i** and hydrochloride salts **324a**–**d** and evaluated the in vitro activities of all these compounds against *S. aureus* ATCC 29213, *S. aureus* N 315, *B. subtilis* ATCC 21216, *E. coli* ATCC 25922, *P. aeruginosa* ATCC 27853, and *P. hauseri* ATCC 13315 [60,199]. The synthesis of these hybrids was carried out as shown in Scheme 64.

A mixture of 1 eq of halobenzyl halides **325** and 1.2 eq of diethylethanolamine (**73**) in MeCN was stirred at 50 °C providing compounds **75** in excellent yields. The subsequent reaction of these diols with PBr_3_ in CHCl_3_ at room temperature for 2 h and then at 60 °C provided compounds **76** in 73–96% yield. Finally, treatment of a THF solution of the latter compounds with the sodium salts prepared from NaH and the appropriate 1*H*-imidazoles **157** gave amine-derived bis-imidazoles **323a**–**i** in yields ranging from 58 to 87%. Compounds **323a**–**d** were then converted to the corresponding hydrochlorides **324a**–**d** in good yields by treatment with HCl in a 4:1 mixture of Et_2_O and CHCl_3_ [199].

Notably, compounds **323a**–**c** exhibited remarkable antibacterial activities against all the tested bacterial strains and, among these hybrids, compound **323b** with an MIC value of 11 μM against *P. aeruginosa* proved to be, on a molar basis, 18 times more potent than the reference drug chloramphenicol. Remarkably, a significant improvement in the bacterial activity was in general observed when some of the above-mentioned azole compounds were converted to the corresponding water-soluble hydrochlorides **324**. The MIC values of these salts ranged from 3 to 150 μM [199].

**Scheme 65:**
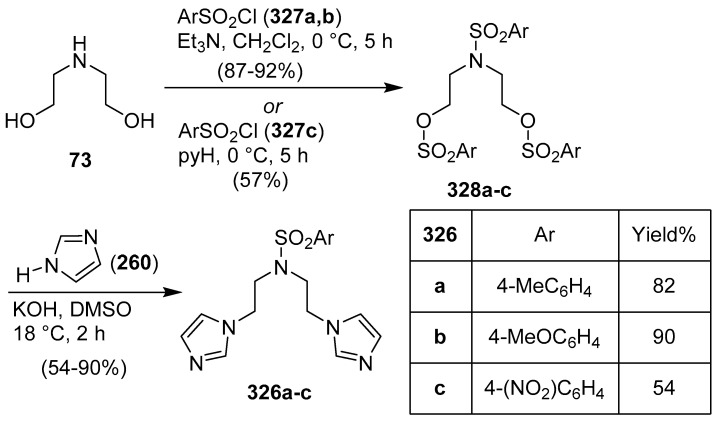
Synthesis of bis-imidazole sulfonamides **326a**–**c.**

In 2013, Al-Mohammed, Abdullah, and coworkers synthesised bis-imidazole sulfonamides **326a**–**c** by reaction of diethanolamine (**73**) with the appropriate 4-substituted benzenesulfonyl chlorides **327**, followed by treatment of the resulting compounds **328** with imidazole (**260**) under basic conditions (Scheme 65) [200].

The in vitro antibacterial activities of compounds **326a**–**c** against *E. coli*, *S. aureus*, *S.* Typhimurium, *B. subtilis*, *P. aeruginosa*, *Rhodococcus ruber*, *Acinetobacter calcoaceticus*, *E. faecalis*, *S. pyogenes*, and *S. epidermidis* were evaluated using the microbroth dilution assay using amoxicillin and kanamycin B as reference drugs, and it was found that the majority of the compounds investigated possessed significant antibacterial activity towards most of these microorganisms. It was specifically found that *N*,*N*-bis[2-(1*H*-imidazol-1-yl)ethyl]-4-methoxybenzenesulfonamide (**326b**) had an MIC value of 800 µM towards *P. aeruginosa* and an MIC value of 270 µM towards *B. subtilis*, while the MIC value of kanamycin B for both of these bacterial species was < 100 µM. Furthermore, bis-imidazoles **326a** and **326b** demonstrated inhibitory effects ranging between 830 and 930 µM against the Gram-positive bacterium *E. faecalis*, while the antibiotic kanamycin B was inactive against this bacterium at the concentration range of this study (<1 mM) [200].

**Scheme 66:**
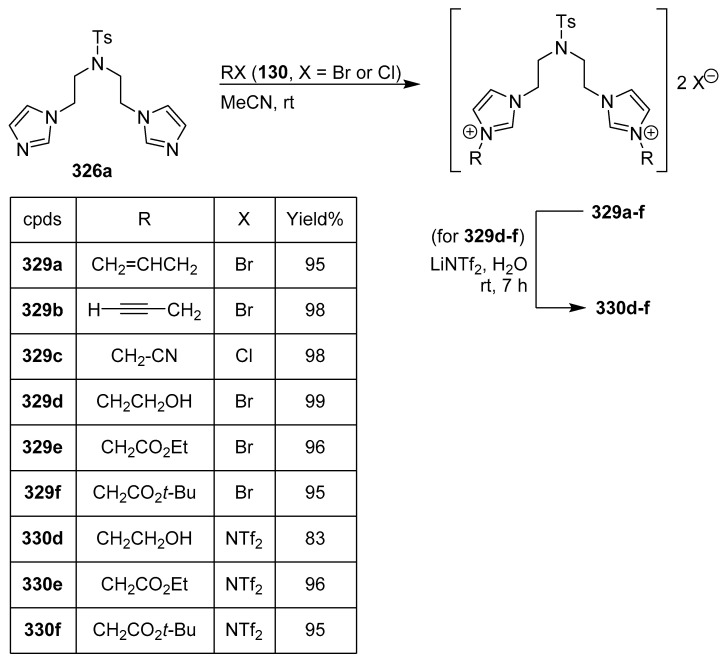
Synthesis of imidazolium geminal dicationic ionic liquids **329a**–**f** and **330d**–**f.**

In 2015, Al-Mohammed, Alias, and Abdullah synthesised imidazolium geminal dicationic ionic liquids **329a**–**f** in good to excellent yields by reaction of *N*,*N*-bis[2-(1*H*-imidazol-1-yl)ethyl]-4-methylbenzenesulfonamide (**326a**) with a molar excess of the appropriate alkyl bromides or chlorides (**130**) in MeCN at 20–55 °C under an N_2_ atmosphere (Scheme 66) [201]. Compounds **329d**–**f** were then converted to the corresponding bis(trifluoromethylsulfonyl)amides **330d**–**f** in good to excellent yields by treatment with lithium bis(trifluoromethanesulfonyl)imide [LiNTf_2_] in deionised water at room temperature (Scheme 66) [201]. Interestingly, all the ionic liquids thus prepared turned out to be miscible with water, acetone, AcOEt, THF, and CHCl_3_, but immiscible with hexane.

Notably, most of these compounds were found to possess significant antibacterial activity against six Gram-negative bacteria, namely *E. coli*, *S.* Typhimurium, *P. aeruginosa*, *A. calcoaceticus*, *S. pyogenes*, and *S. aureus*, and against four Gram-positive bacteria, namely *B. subtilis*, *R. ruber*, *E. faecalis*, and *S. epidermidis*. High antibacterial toxicity against all the tested bacteria was exhibited by the ionic liquid **329c** with acetonitrile substituent. Moreover, ionic liquids **329d** and **329e** demonstrated bacterial inhibition of *E. faecalis* with an MIC value of 410 and 360 µM, respectively. It was also found that all ionic liquids, except 1,1′-[(tosylazanediyl)bis(ethane-2,1-diyl)]bis(3-propargyl-1*H*-imidazol-3-ium) bromide (**329b**), possessed significant antibacterial activities against β-lactam resistant *P. aeruginosa* with MIC values ranging from 200 to 500 µM. Finally, it was observed that **329c** exhibited considerable antibacterial activity (MIC = 100 µM) against *B. subtilis*, a bacterial species that required a high dose (680 µM) of amoxicillin [201].

**Scheme 67:**
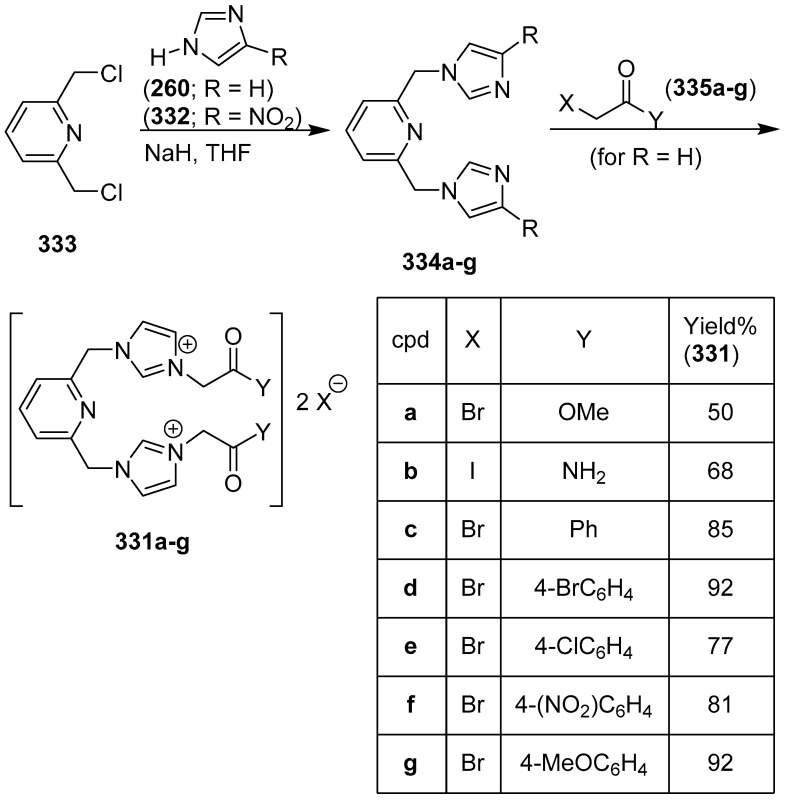
Synthesis of bis(imidazole)-pyridines **331a**–**g.**

In 2020, Mangalagiu, Mangalagiu, and coworkers designed, synthesised, and evaluated the antimycobacterial activity of bis(imidazole)pyridines **331a**–**g** [202]. The efficient and straightforward synthesis of these new quaternary salts was carried out by an approach in which a solution of 3 eq of 1*H*-imidazole (**260**) or 4-nitro-1*H*-imidazole (**332**) in a mixture of THF and DMF was treated with a suspension of 3.6 eq of NaH in THF and 1 eq of 2,6-bis(chloromethyl)pyridine (**333**) at room temperature. The resulting compounds **334a**–**g** were then treated with a solution of 2.4–2.8 eq of α-halocarbonyl derivatives **335a**–**g** giving rise to the required quaternary salts **331a**–**g** in yields ranging from 50 to 92% (Scheme 67) [202].

These derivatives, which were soluble at a high concentration (200 μM) in microbiological medium, were evaluated for their in vitro antimycobacterial activity against *M. tuberculosis* H37Rv grown under aerobic conditions. Among the tested quaternary ammonium salts, 1,1′-[pyridine-2,6-diylbis(methylene)]bis{3-[2-(4-chlorophenyl)-2-oxoethyl]-1*H*-imidazol-3-ium} bromide (**331e**) and 1,1′-[pyridine-2,6-diylbis(methylene)]bis{3-[2-(4-nitrophenyl)-2-oxoethyl]-1*H*-imidazol-3-ium} bromide (**331f**) turned out to have an excellent anti-TB activity against *M. tuberculosis* H37Rv under aerobic condition with MIC values of 51 and 58 μM, respectively, and IC_50_ values of 34 and 38 μM, respectively. Furthermore, they proved to be not cytotoxic and exhibited a very good intracellular activity [202].

## 13. Pyridine/Imidazole Hybrids

**Figure 12:**
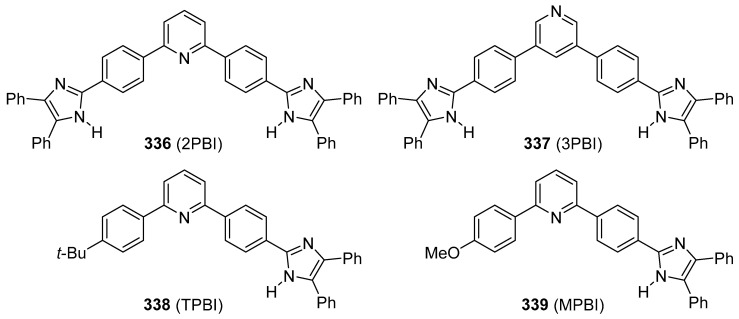
Structures of imidazole-pyridine fluorophores 2PBI (**336**), 3PBI (**337**), TPBI (**338**), and MPBI (**339**).

In 2013, in the context of a study on the identification and development of new antibacterial substances effective against resistant bacterial strains, Renganathan and coworkers synthesised four imidazole-pyridine fluorophores, i.e., 2,6-bis[4-(4,5-diphenyl-1*H*-imidazol-2-yl)phenyl]pyridine (2PBI) (**336**), 3,5-bis[4-(4,5-diphenyl-1*H*-imidazol-2-yl)phenyl]pyridine (3PBI) (**337**), 2-[4-(*tert*-butyl) phenyl]-6-[4-(4,5-diphenyl-1*H*-imidazol-2-yl)phenyl]pyridine (TPBI) (**338**), and 2-[4-(4,5-diphenyl-1*H*-imidazol-2-yl)phenyl]-6-(4-methoxyphenyl)pyridine (MPBI) (**339**) (Figure 12), by using Pd(PPh_3_)_4_-catalysed Suzuki reactions followed by AcOH-mediated three component cyclisation reactions [203].

**Scheme 68:**
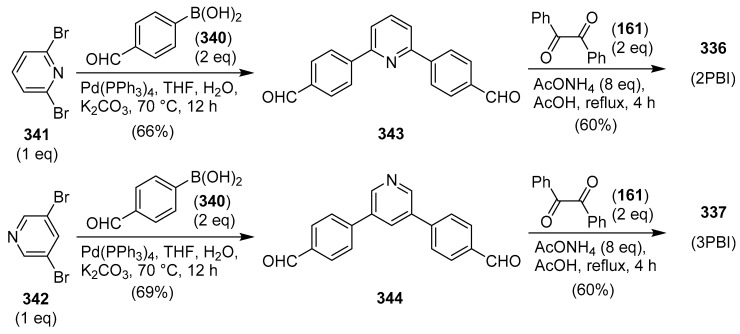
Synthesis of hybrids 2PBI (**336**) and 3PBI (**337**).

As shown in Scheme 68, compounds **336** and **337** were synthesised by Pd(PPh_3_)-catalysed cross-coupling reaction of 2.0 eq of 4-formylboronic acid (**340**) with 1.0 eq of 2,6-dibromopyridine (**341**) and 1.0 eq of 3,5-dibromopyridine (**342**), respectively, in a mixture of THF and water at 70 °C for 12 h using K_2_CO_3_ as base followed by treatment of the resulting compounds **343** and **344**, respectively, with 2 eq of benzil (**161)** and 8 eq of AcONH_4_ in AcOH under reflux for 4 h. In this way, compounds **336** and **337** were obtained in 40 and 41% overall yield, respectively [203].

**Scheme 69:**
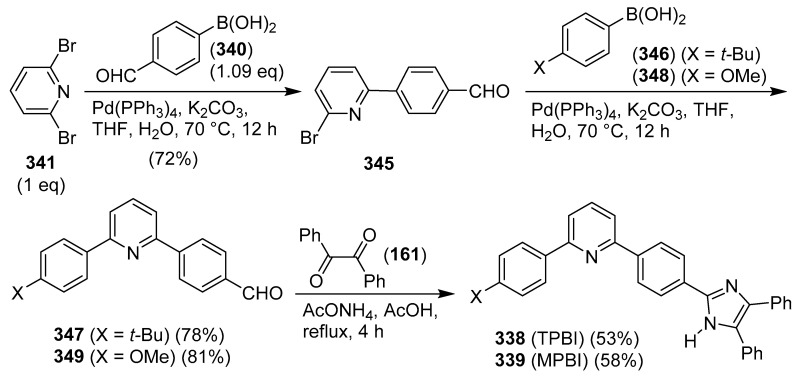
Synthesis of imidazole-pyridine fluorophores TPBI (**338**) and MPBI (**339**).

A different protocol was instead used to prepare compounds **338** and **339**. As shown in Scheme 69, **341** was treated with 1.09 eq of **340** in a mixture of THF and water at 70 °C for 12 h in the presence of 5 mol% Pd(PPh_3_)_4_ and 3 eq of K_2_CO_3_ providing 4-(6-bromopyridin-2-yl)benzaldehyde (**345**) in 72% yield. The Pd(PPh_3_)_4_-catalysed Suzuki coupling of **345** with an equimolar amount of 4-(*tert*-butyl)phenylboronic acid (**346**) gave 4-{6-[4-(*tert*-butyl)phenyl]pyridin-2-yl}benzaldehyde (**347**) in 78% yield. The subsequent cyclisation reaction of this pyridine derivative with 1.09 eq of benzil (**161**) and 7.3 eq of AcONH_4_ in AcOH at 120 °C for 4 h then gave imidazole-pyridine fluorophore **338** in 53% yield. On the other hand, the Pd(PPh_3_)_4_-catalysed Suzuki coupling of **345** with 4-methoxyphenylboronic acid (**348**) provided compound **349** in 81% yield. This 2,6-diarylpyridine was then converted to **339** in 58% yield by cyclisation with benzil (**161**) and AcONH_4_ in AcOH at 120 °C for 4 h (Scheme 69) [203].

UV-Vis and fluorescence studies demonstrated that hybrids **336**, **337**, **338**, and **339** had excellent fluorescence properties with high quantum efficiencies, they were sensitive to the polarity of the microenvironment, and that the effect of solvent polarity on their absorption behaviour was minimal. In addition, studies on the in vitro antibacterial activity of these fluorophores revealed that **339** had relatively good inhibitory power against Gram-negative strains (*E. coli* MTCC 2939, *P. aeruginosa* MTCC 1934, *Aeromonas hydrophila* MTCC 1739), while **338** exhibited antibacterial activity against Gram-positive *Rhodococcus rhodochrous* MTCC 265 [203].

In 2015, Gomha and coworkers [204] reported the synthesis of pyridines **350a**–**e** and **351a**–**e** containing imidazole moiety and bipyridine derivatives **352**, **353**, and **354** in good yields via one-pot multicomponent condensation reactions [9] and evaluated the in vitro antibacterial activities of these compounds against Gram-positive and Gram-negative bacteria.

**Scheme 70:**
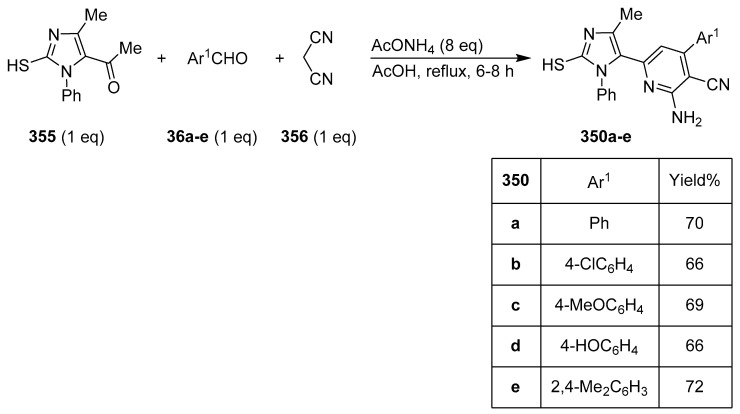
Synthesis of compounds **350a**–**e.**

Compounds **350** were prepared in 66–72% yield by one-pot condensation of 1-(4-methyl-1-phenyl-2-sulfanyl-1*H*-imidazol-5-yl)ethan-1-one (**355**) [205], the appropriate aryl aldehydes **36**, malononitrile (**356**), and AcONH_4_ in AcOH under reflux (Scheme 70) [204].

**Scheme 71:**
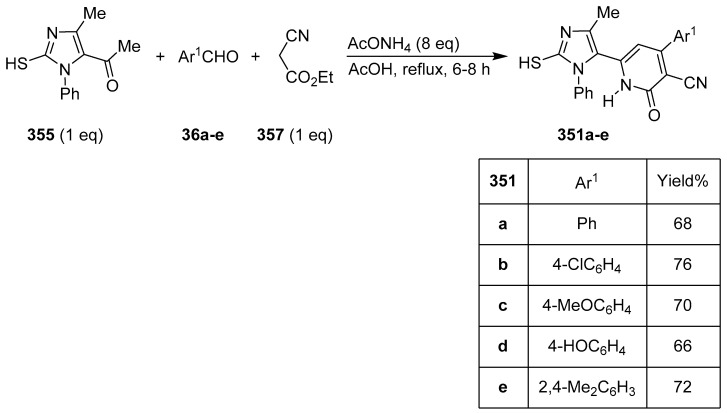
Synthesis of compounds **351a**–**e.**

Compounds **351** were prepared in similar manner in 66–76% yield by one-pot condensation of **355** with aldehydes **36**, ethyl cyanoacetate (**357**), and a molar excess of AcONH_4_ in refluxing AcOH (Scheme 71) [204].

**Scheme 72:**
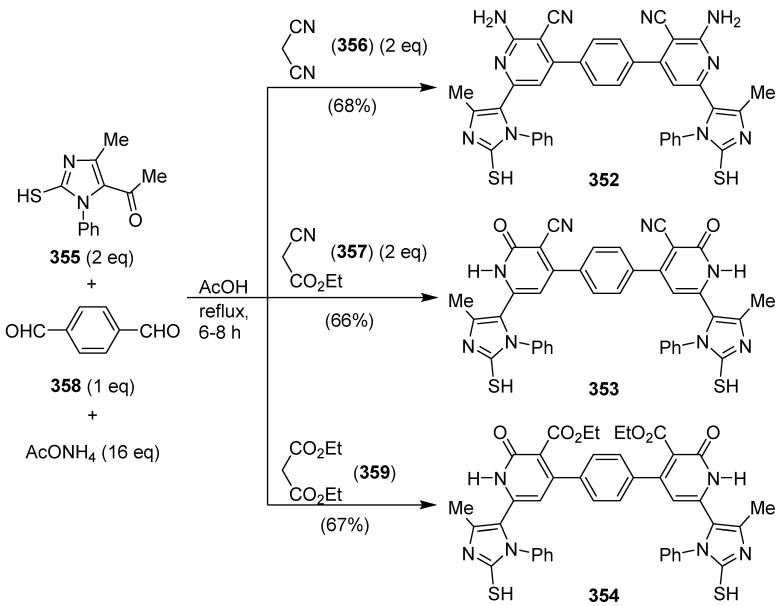
Synthesis of bipyridine derivatives **352**, **353**, and **354.**

The protocol involving the one-pot cyclocondensation reaction of Scheme 71 was then extended to the synthesis of bispyridine derivatives **352**, **353**, and **354**. In particular, 2 eq of **355** were treated with 1 eq of terephthaldehyde (**358**), 2 eq of malononitrile (**356**), and 16 eq of AcONH_4_ in AcOH under reflux for 6–8 h, providing 4,4′-(1,4-phenylene)bis[2-amino-6-(4-methyl-1-phenyl-2-sulfanyl-1*H*-imidazol-5-yl)nicotinonitrile] (**352**) in 68% yield (Scheme 72). When ethyl cyanoacetate (**357**) and diethyl malonate (**359**) replaced malononitrile, the cyclocondensation reaction gave rise to bispyridine derivatives **353** and **354**, respectively, in 66–67% yield (Scheme 72) [204].

The in vitro antibacterial activity of all the hybrids so synthesised was evaluated against the Gram-positive bacteria *S. pneumoniae* and *B. subtilis*, and the Gram-negative bacteria *P. aeruginosa* and *E. coli*. Ampicillin and gentamicin were used as the reference drugs for the Gram-positive bacteria and the Gram-negative bacteria, respectively. Compounds **350c** and **350d** were found to exhibit high inhibitory activity against *S. pneumoniae* while compounds **350a**, **350b**, **350e**, and **351a** showed moderate inhibitory effects. Imidazole derivatives **350c**, **350d**, **351a**, **351e**, **352**, **353**, and **354** exhibited high inhibitory effects against *B. subtilis*. For example, the zone of inhibition of **350c** was 20.8 ± 0.6 mm and that of ampicillin was 32.4 ± 0.3 mm. Furthermore, compounds **350c**, **350d**, **351a**, **351e**, and **354** exhibited high inhibitory effects against *E. coli*. For example, the zone of inhibition of **350d** was 19.9 ± 0.4 mm and that of the reference compound, gentamicin, was 19.9 ± 0.3 mm [204].

**Scheme 73:**
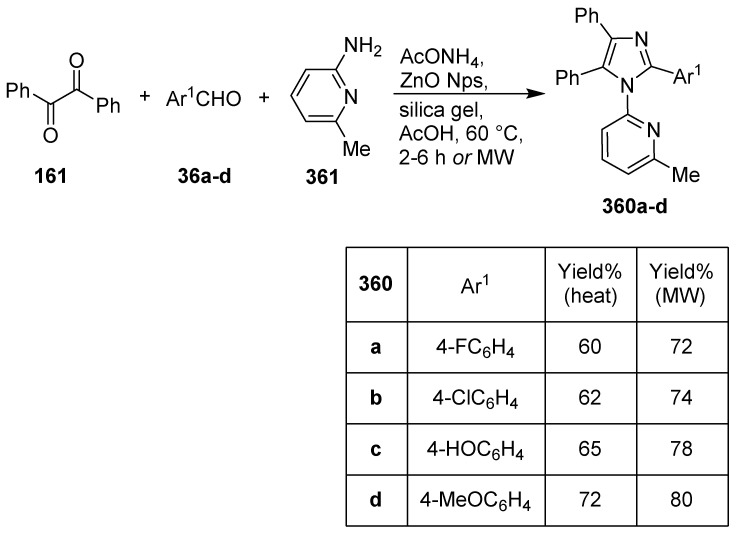
Synthesis of 2-(2-aryl-4,5-diphenyl-1*H*-imidazol-1-yl)-6-methylpyridines **360a**–**d.**

Finally, in 2019, Narayana and coworkers synthesised 2-(2-aryl-4,5-diphenyl-(1*H*-imidazol-1-yl)-6-methylpyridines **360a**–**d** by one-pot cyclocondensation of benzil (**161**) with aryl aldehydes **36a**–**j**, 6-methylpyridin-2-amine (**361**), and AcONH_4_ in glacial acetic acid at 60 °C using conventional heating for 3 h or microwave irradiation for 20–25 min in the presence of ZnO nanoparticles (10 mol%) as effective catalyst (Scheme 73) [166]. The yields ranged from 60 to 72% using conventional heating and were higher (72–80%) when the reactions were carried out under MW irradiation. It is also worth noting that in the paper by Narayana and coworkers the structure of **361** was wrongly reported. Compounds **360a**–**d** were then evaluated for their in vitro antibacterial activity using the resazurin reduction assay and it was found that the IC_50_ values of **360a** towards *S. aureus* and *E. coli* were 96 and 47 µM, respectively [166].

## 14. Quinoline/Imidazole Hybrids

Quinoline derivatives including 4-quinolones have attracted considerable attention because of their presence in the skeleton of several antibacterial drugs [206,207,208,209,210,211]. In recent years, efforts have also been directed to the design, synthesis, and evaluation of the antimicrobial properties of quinoline-imidazole derivatives.

**Scheme 74:**
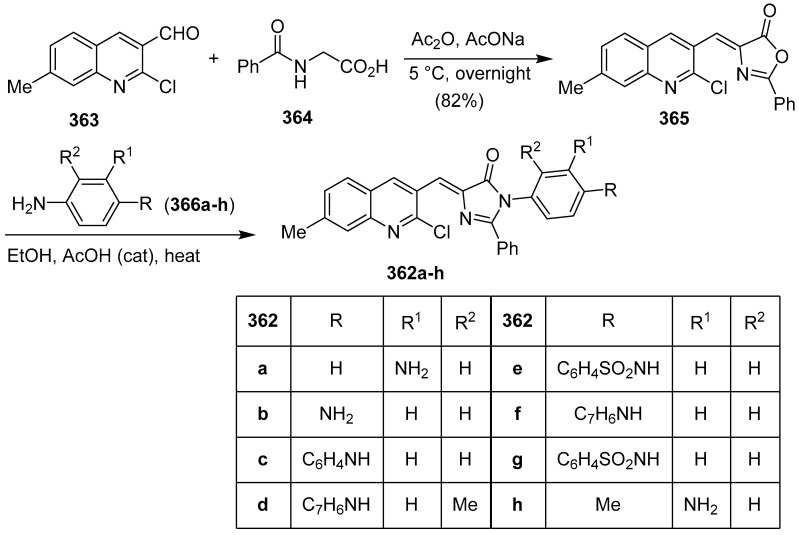
Synthesis of 2-chloro-7-methylquinoline-3-carbaldehyde-based imidazole derivatives, **362a**–**h.**

In 2012, Parab and Dixit synthesised and characterised 2-chloro-3-formyl-7-methylquinoline/imidazole hybrids **362a**–**h** based on 2-chloro-7-methylquinoline-3-carbaldehyde (**363**) and tested their antibacterial activities against the Gram-negative bacteria *E. coli* and *P. aeruginosa* and the Gram-positive bacteria *Bacillus subtilis* and *B. megaterium* [212]. As shown in Scheme 74, one of these hybrid, (*Z*)-3-(3-aminophenyl)-5-[(2-chloro-7-methyl-3-quinolinyl) methylene]-2-phenyl-3,5-dihydro-4*H*-imidazol-4-one (**362a**) was synthesised in 82% yield by refluxing 1 eq of 2-chloro-7-methylquinoline-3-carbaldehyde (**363)** and 1 eq of hippuric acid (**364**) in Ac_2_O with freshly prepared AcONa for 2 h, followed by addition of the resulting compound **365** to a solution of diamine **366a** in EtOH containing few drop of glacial AcOH and subsequent heating. Compound **363** was in turn synthesised by a Vilsmeier–Haack reaction according to a procedure reported in the literature [213,214]. The other 2-chloro-7-methylquinoline-3-carbaldehyde-based imidazole derivatives, **362b**–**h**, were prepared in a similar manner from **363** by using diamines **366b**–**h**, but, unfortunately, the yields of many reactions illustrated in Scheme 74 were not reported.

Nevertheless, compounds **362a**–**h** were evaluated for their in vitro antibacterial activities and the results of these tests showed that hybrids **362a**–**c** exhibited moderate inhibition against the above-mentioned Gram-negative bacterial species and especially against *E. coli*, while all hybrids displayed good activity against the Gram-positive organisms. In particular the inhibition zones of **362b** and **362c** against *B. megaterium* were 28 and 30 mm, respectively, while the inhibition zone of streptomycin, the reference drug, was 36 mm [212].

**Scheme 75:**
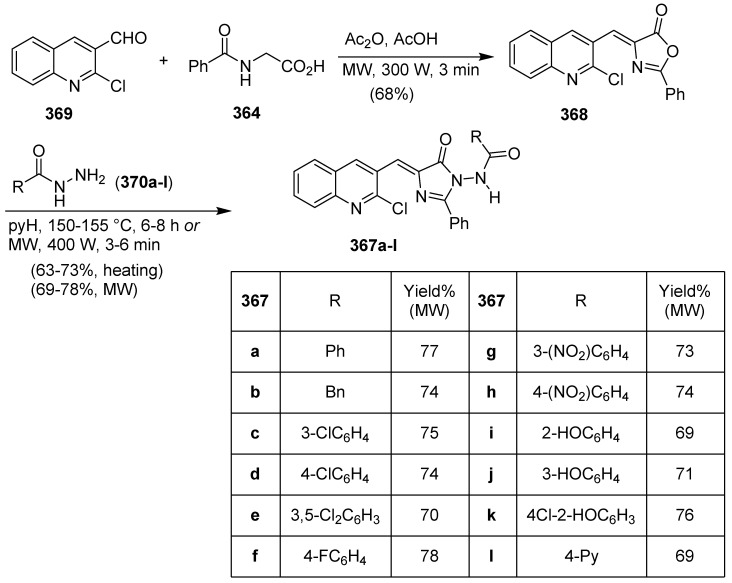
Synthesis of (*Z*)-*N*-{4-[(2-chloroquinolin-3-yl)methylene]-5-oxo-2-phenyl-4,5-dihydro-1*H*-imidazol-1-yl}arenamides **367a**–**l.**

In 2014, Desai and coworkers described the synthesis and characterisation of (*Z*)-*N*-{4-[(2-chloroquinolin-3-yl)methylene]-5-oxo-2-phenyl-4,5-dihydro-1*H*-imidazol-1-yl} arenamides **367a**–**l** (Scheme 75) and screened the in vitro antimicrobial activity of these quinoline based imidazole derivatives against *E. coli* MTCC 443, *P. aeruginosa* MTCC 1688, *S. aureus* MTCC 96, and *S. pyogenes* MTCC 442 [215]. (*Z*)-4-[(2-Chloroquinolin-3-yl)methylene]-2-phenyloxazol-5(4*H*)-one (**368**), which was the key synthetic intermediate, was prepared in 68% yield by microwave-promoted reaction of 2-chloroquinoline-3-carbaldehyde (**369**) [215] with hippuric acid (**364**). Compounds **367** were then prepared in 63–73% yield by heating **368** with equimolar amounts of the appropriate arene carbohydrazides **370** in pyridine in an oil bath at 150–155 °C for 6–8 h. Nevertheless, higher yields (69–78%) of compounds **367a**–**l** were obtained by reaction between **368** and **370** under irradiation in a microwave oven at 400 W for 3–6 min.

The results of the evaluation of the antibacterial activity of hybrids **367a**–**l** showed that: (i) compounds **367d** and **367h** exhibited activity (MIC = 50 μM) against *E. coli* higher than that of ampicillin, the reference drug (MIC = 290 μM); (ii) compounds **367h** and **367j** were highly potent against P. aeruginosa with MIC values of 100 and 110 μM, respectively; (iii) compounds **367f** and **367i** with an MIC value of 210 μM for each of them had remarkable antibacterial activity against S. aureus; and (iv) compound **367d** showed significant potency (MIC = 100 μM) against *S. pyogenes* [215], an aerotolerant Gram-positive bacterium that is the cause of strep throat.

**Scheme 76:**
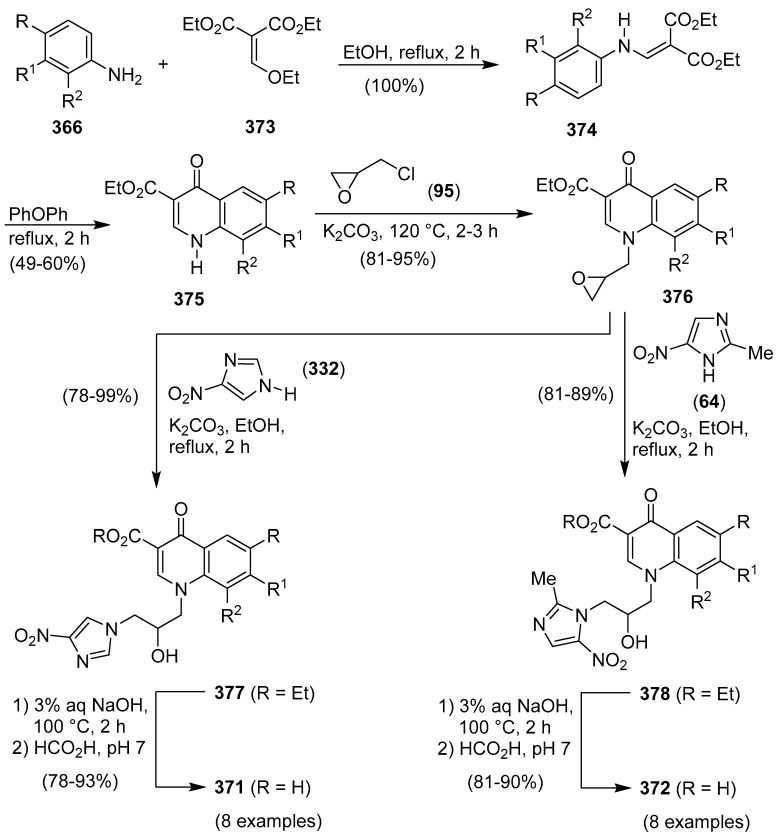
Synthesis of 4-quinolone/imidazole hybrids **371** and **372**.

Still in 2014, Zhou and coworkers [216] designed and synthesised two novel series of 4-quinolone/imidazole hybrids **371** and **372**. These compounds were prepared according to the route outlined in Scheme 76. In particular, arenamines **366** were treated with commercially available ethoxymethylene malonic acid ester **373** in EtOH under reflux providing compounds **374** in almost quantitative yields. Treatment of these intermediates with diphenyl ether under reflux for 2 h afforded quinolones **375** in yields ranging from 49 to 60%. The subsequent reaction of these compounds with a large molar excess of 2-(chloromethyl)oxirane (**95**) under reflux for 2–3 h in the presence of K_2_CO_3_ gave the ethyl 1-(oxiran-2-ylmethyl)-4-oxo-1,4-dihydroquinoline-3-carboxylate derivatives **376** in 81–95% yield. These compounds were then treated with 4-nitro-1*H*-imidazole (**332**) in EtOH under reflux for 2 h in the presence of K_2_CO_3_ providing compounds **377** in 80–99% yield. Finally, saponification of these derivatives with 3% NaOH at 100 °C for 2 h, followed by acidification with formic acid to pH 7 afforded hybrids **371** in yields ranging from 78 to 93%. Similarly, the reaction of compounds **376** with 2-methyl-5-nitro-1*H*-imidazole (**64**) in refluxing EtOH in the presence of K_2_CO_3_ provided compounds **378** in 81–89% yield. Finally, saponification of these derivatives followed by acidification produced hybrids **372** in yields ranging from 81 to 90% [216].

Subsequently, hybrids **371** and **372** and their direct precursors, compounds **377** and **378**, respectively, were evaluated for their antibacterial activities against the Gram-positive bacteria *S. aureus* ATCC 25923, *S. pneumoniae*, *S. aureus* N315, *M. luteus* ATCC 4698, MRSA, *B. subtilis* ATCC 6633, and against the Gram-negative bacteria *P. aeruginosa*, *E. coli* DH52, *S. dysenteriae*, and *S.* Typhi [217] and it was found that many of the tested compounds, at low concentrations, exhibited good or even higher antimicrobial activities in comparison with the reference drugs chloramphenicol [50] and norfloxacin. It was also discovered that 1-[2-hydroxy-3-(2-methyl-5-nitro-1*H*-imidazol-1-yl)propyl]-4-oxo-7-trifluoromethyl-1,4-dihydroquinoline-3-carboxylic acid (**372d**) had in vitro an MIC value of 2 μM against MRSA and was more active than its ethyl ester precursor, **378d**. Hybrid **372d** also had an MIC value of 9 μM against *S. pneumoniae* and was more potent than chloramphenicol, which had an MIC value of 50 μM. The results of the study by Zhou and coworkers finally showed that hybrids bearing a 2-methyl-5-nitroimidazolyl moiety exhibited antibacterial activities higher than those of hybrids bearing a 4-nitroimidazolyl moiety [216]. Zhou and coworkers also investigated the mechanism of action of **372d** by studying the interactions of this hybrid with calf thymus DNA and found that non-covalent interaction between **372d** and TOPO IV DNA complex, especially hydrogen bonds between **372d** and Ser79, was involved [216].

**Scheme 77:**
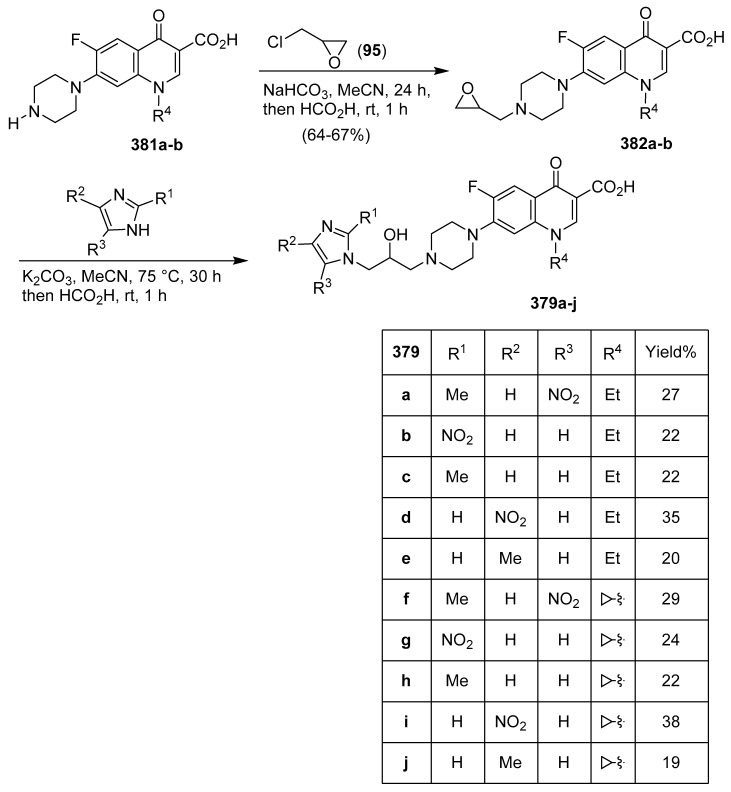
Synthesis of hybrids **379a**–**j.**

In 2015, Zhou and coworkers, as part of their studies on the synthesis and biological evaluation of quinolone/azole hybrids [61], synthesised several 4-quinolone/imidazole hybrids **379a**–**j** and **380a**–**e** as new types of antimicrobial agents [218]. Hybrids **379** were synthesised using the route shown in Scheme 77. In particular, norfloxacin (**381a**, R^4^ = Et) was treated with an equimolar amount of 2-(chloromethyl)oxirane (**95**) in MeCN at 50 °C for 10 h in the presence of NaHCO_3_ and then treated with formic acid to adjust the pH value to 5.5–6.5 providing 1-ethyl-6-fluoro-7-[4-(oxiran-2-ylmethyl)piperazin-1-yl]-4-oxo-1,4-dihydroquinoline-3-carboxylic acid (**382a**) in 67% yield. Using similar experimental conditions compound **381b** (R^4^ = cyclopropyl) was converted to carboxylic acid **382b** in 64% yield. Subsequently, a mixture of **382a** and 2-methyl-5-nitro-1*H*-imidazole (**64**) in MeCN was stirred at 75 °C in the presence of K_2_CO_3_, then cooled to room temperature and treated with formic acid to adjust the pH value to 5.5–6.5 providing compound **379a** in 27% yield. A similar procedure was then used to prepare hybrids **379b**–**j** from **382b** and the appropriately substituted free-NH imidazoles [218].

**Scheme 78:**
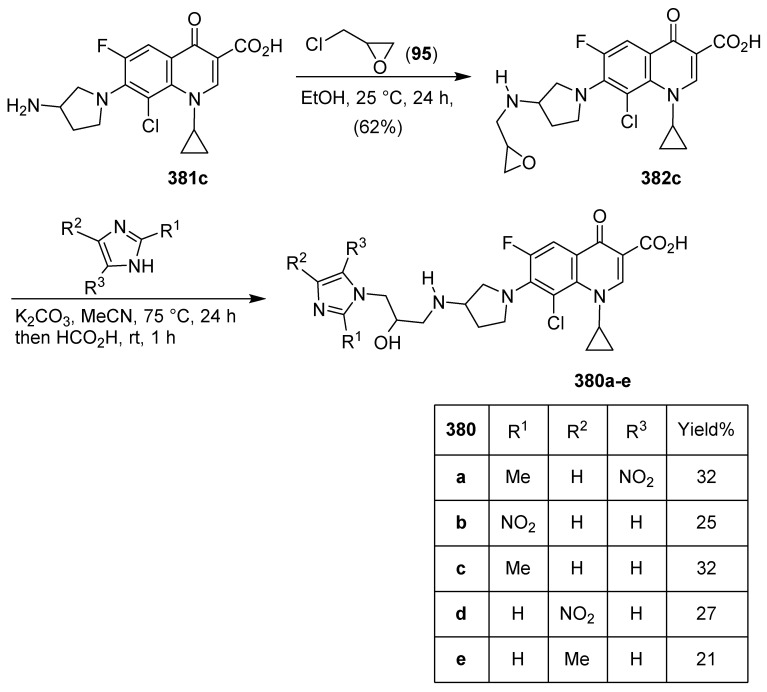
Synthesis of hybrids **380a**–**e.**

On the other hand, hybrids **380a**–**e** were synthesised in two steps from the fluoroquinolone antibiotic clinafloxacin (**381c**) via the route shown in Scheme 78 that involved the use of a protocol similar to that developed for the synthesis of hybrids **379a**–**j** [218].

Hybrids **379a**–**j** and **380a**–**e** were then tested in vitro for their antimicrobial activities against the Gram-positive bacteria *M. luteus* ATCC 4698, MRSA N315, *S. aureus* ATCC 25923, and *B. subtilis* ATCC 2126 and against the Gram-negative bacteria *P. aeruginosa*, *E. coli* JM109, *P. hauseri* [60], and *S.* Typhi [217]. Chloramphenicol, norfloxacin, ciprofloxacin, and clinafloxacin were used as reference drugs. Most hybrids exhibited good bioactivities and compounds **379a**, **379e**, **379g**, **379j**, **380b**, and **380c** exhibited remarkable activity against MRSA with MIC values ranging from 0.39 to 6.6 µM, which were lower than that of chloramphenicol (MIC = 36 µM). With regard to units of measurement that were used to express MIC values, we wish to point out that in our opinion the values of these measurements were expressed by the authors using incorrect units instead of reporting them using µM values.

Notably, hybrid **380b** was found to inhibit the growth of all bacterial strains tested with MIC values ranging from 97 nM to 1.4 µM and demonstrated excellent activity against *E. coli* with an MIC value of 97 nM. Moreover, the tested quinolone/imidazoles were found to inhibit induced bacterial resistance more slowly than clinical drug controls also exhibiting low cytotoxicity to human cells. The interactions of hybrid **380b**, Cu^2+^, and MRSA DNA were also investigated, and it was discovered that **380b** could intercalate into DNA through Cu^2+^ bridge to form a steady **380b**, Cu^2+^, and MRSA DNA ternary complex, which might further block DNA replication. It was finally found that **380b** could be effectively stored and carried by human serum albumin [218].

**Scheme 79:**
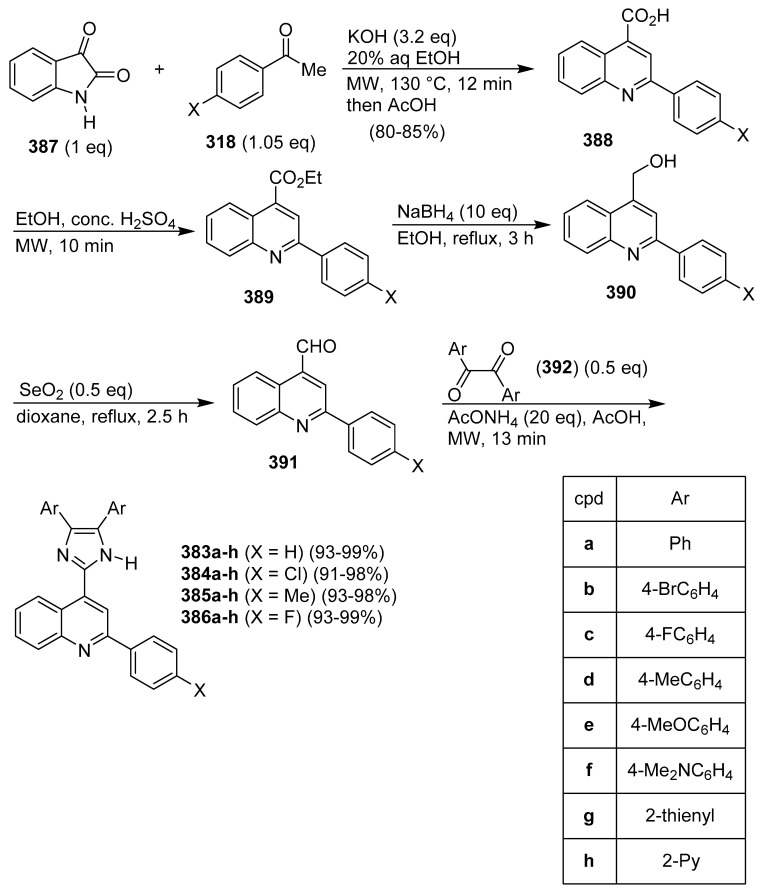
Synthesis of quinoline/imidazole hybrids **383a**–**h**, **384a**–**h**, **385a**–**h**, and **386a**–**h.**

In 2016, Al-Qawasmeh, Al-Tel, and coworkers synthesised quinoline/imidazole hybrids **383a**–**h**, **384a**–**h**, **385a**–**h**, and **386a**–**h** and tested the activity of these 4-(4,5-diaryl-1*H*-imidazol-2-yl)-2-arylquinoline derivatives against a range of bacterial strains [219]. These hybrids were synthesised through the route shown in Scheme 79, in which the first step involved the condensation reaction of 1.0 eq of isatin (**387**) with 1.05 eq of acetophenones **318** in the presence of 3.2 eq of KOH and 20% aqueous EtOH under irradiation by MW with variable power to keep a constant temperature at 130 °C for 12 min, followed by acidification with AcOH.

Esterification of the resulting carboxylic acids **388** with EtOH under microwave irradiation in the presence of conc. sulfuric acid produced esters **389**, which were reduced using NaBH_4_ in EtOH, providing alcohols **390**. Subsequent oxidation of these intermediates by treatment with SeO_2_ in dioxane under reflux gave aldehydes **391** in high yields. Finally, the reaction of these compounds with α-diketones **392** and AcONH_4_ in AcOH under microwave irradiation led to the required hybrids **383**, **384**, **385**, and **386** [219].

These compounds were then tested against the Gram-negative bacteria *E. coli* ATCC 25922, ATCC strains of *K. pneumoniae*, *P. vulgaris* clinical isolate, *P. aeruginosa* clinical isolate, as well as against Gram-positive pathogens including two strains of *S. aureus* (methicillin-sensitive and methicillin-resistant) and *S. epidermidis* and turned out to be generally more active against Gram-positive bacterial species than Gram-negative bacteria, although most of these hybrids did inhibit the growth of *E. coli* and *P. aeruginosa* in a low MIC range. Only hybrids **384b**, **384c**, **384h**, **386c**, and **386h** inhibited *E. coli* with MIC values less than 24–34 µM and were more potent than ceftriaxone, a third-generation cephalosporin, which was used as a positive control (56 µM). It was also observed that the most potent hybrids possessed hydrophobic and electron-withdrawing functionalities (**384b**, **384c**, **386b**, and **386c**), such as halogens, on their aryl arms [219].

**Scheme 80:**
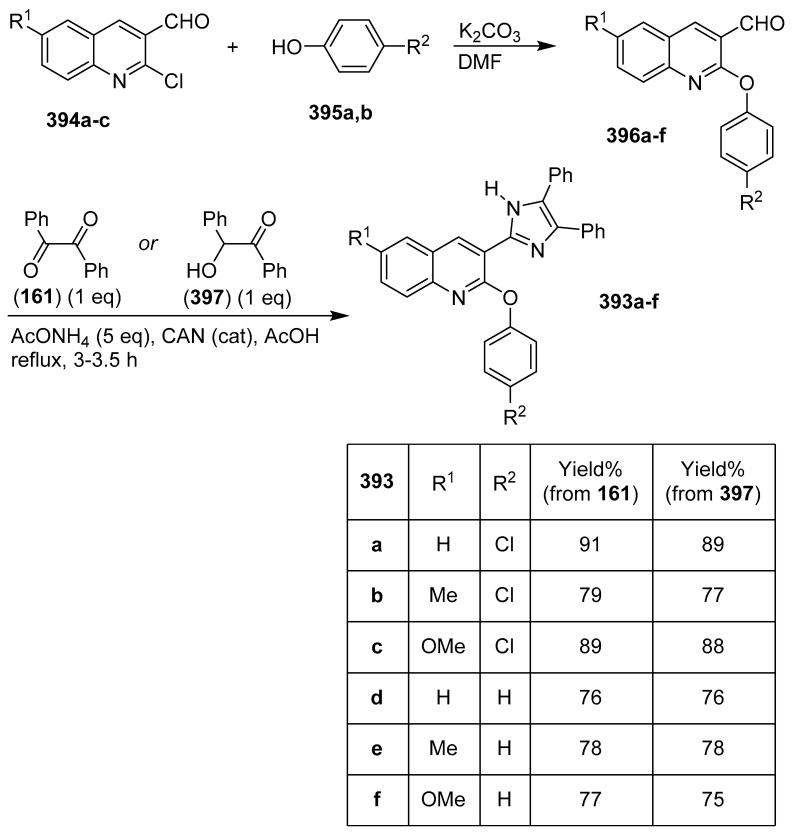
Synthesis of hybrids **393a**–**f.**

In 2018, Patel and coworkers synthesised imidazole derivatives **393a**–**f** bearing quinoline nucleus and screened these compounds for their antimicrobial and antitubercular activities [220]. As outlined in Scheme 80, their optimised synthesis began with the reaction of 2-chloroquinoline-3-carbaldehydes **394** with phenols **395a** and **395b** in DMF in the presence of K_2_CO_3_. The subsequent reaction of the resulting 2-aryloxy-6-quinoline-3-carbaldehydes **396** with an equimolar amount of benzoin (**397**) or benzil (**161**) and 5 eq of AcONH_4_ in AcOH under reflux, in the presence of a catalytic amount of CAN, provided target hybrids **393** in yields ranging from 75 to 91%.

These compounds were screened against three Gram-positive bacteria, i.e., *B. subtilis* MTCC 441, *Clostridium tetani* MTCC 449, and *S. pneumoniae* MTCC 1936, and three Gram-negative bacteria, i.e., *E. coli* MTCC 443, *S.* Typhi MTCC 98, and *Vibrio cholerae* MTCC 390, using ampicillin, ciprofloxacin, and chloramphenicol as standard antibacterial drugs. Among the tested compounds, hybrid **393c** displayed high activity against *B. subtilis* and *E. coli* with MIC values of 120 μM comparable to that of chloramphenicol (MIC = 150 μM). Hybrids **393e** and **393f** displayed high activity against *S.* Typhi with MIC values of 220 and 210 μM, respectively, and hybrid **393c** displayed activity against *V. cholerae* that, on a molar basis, was lower than that of ampicillin (MIC = 500 and 720 μM, respectively). It was also observed that the in vitro antitubercular activity of several hybrids **393** against the culture of *M. tuberculosis* H_37_R_v_ strain was generally significantly lower than that of the standard drug isoniazid, for which the percentage of inhibition at a concentration of 1.8 mM was 99%. Only hybrid **393c** displayed 84% inhibition [220]. Molecular docking studies were also performed to highlight the interaction of hybrids **393** with GlcN-6-P synthase enzyme and it was found that the antimicrobial compounds **393a**, **393c**, and **393d** exhibited the least binding energy among the tested hybrids [220].

**Scheme 81:**
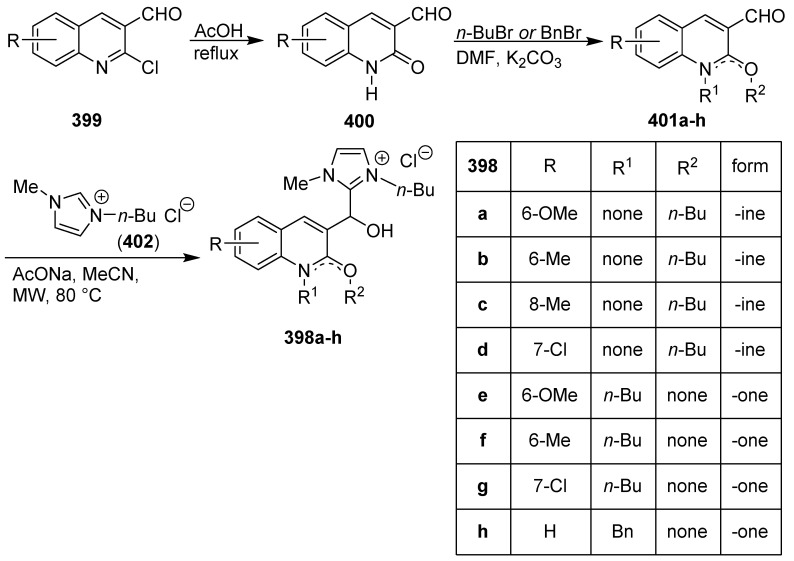
Synthesis of quinoline-based hydroxyimidazolium hybrids **398a**–**h.**

In 2019, Insuasty, Abonia, and coworkers synthesised quinoline-based hydroxyimidazolium hybrids **398a**–**h** and evaluated in vitro their antimicrobial activity against two Gram-negative microorganisms, *E. coli* and *K. pneumoniae*, Gram-positive *S. aureus*, and two acid fast slow-growing mycobacteria, *Mycobacterium tuberculosis* H_37_R_v_ and *M. bovis* BCG [221]. As shown in Scheme 81, the synthesis of hybrids **398** involved the conversion of 2-chloroquinoline-3-carbaldehydes **399** to 2-oxo-1,2-dihydroquinoline-3-carbaldehydes **400**, followed by alkylation of the latter derivatives with *n*-BuBr or BnBr in DMF in the presence of K_2_CO_3_. Aldehydes **401** were then subjected to reaction with 3-butyl-1-methylimidazolium chloride (**402**), in MeCN at 80 °C in the presence of AcONa under ultrasonic irradiation to afford the required hybrids **398** in 60–91% yield [221,222]. Compounds **400** were in turn prepared by a Meth–Cohn reaction [223].

Evaluation tests of antibacterial activity showed that almost all hybrids **398** exhibited low activity against the Gram-negative organisms, but hybrid **398b** demonstrated a potent anti-staphylococcal activity with an MIC value of 5 μM. It was also found that hybrids **398a** and **398b** possessed significant inhibitory activity against *M. tuberculosis* H_37_R_v_ with MIC values of 46 and 24 μM, respectively. The cell wall of this bacterium, which is the causative agent of tuberculosis, has characteristics of both Gram-positive and Gram-negative bacteria. On the other hand, hybrid **398b** at concentrations ranging from 30 to 500 μM demonstrated 100% inhibition of *S. aureus* and at 300 μM demonstrated 100% inhibition of *K. pneumoniae*, and hybrids **398d**, **398e**, and **398h** displayed moderate growth inhibition against the virulent H_37_R_v_ strain with MIC values of 110 μM [221].

## 15. Pyrimidine/Imidazole Hybrids

In recent years, a large number of studies has been done on the synthesis and evaluation of the antibacterial properties of pyrimidine derivatives [224,225,226,227,228,229,230,231] and in this context significant attention has been paid to the identification, synthesis, and evaluation of the antibacterial properties of pyrimidine/imidazole hybrids with potentially significant activity [232,233,234,235].

**Scheme 82:**
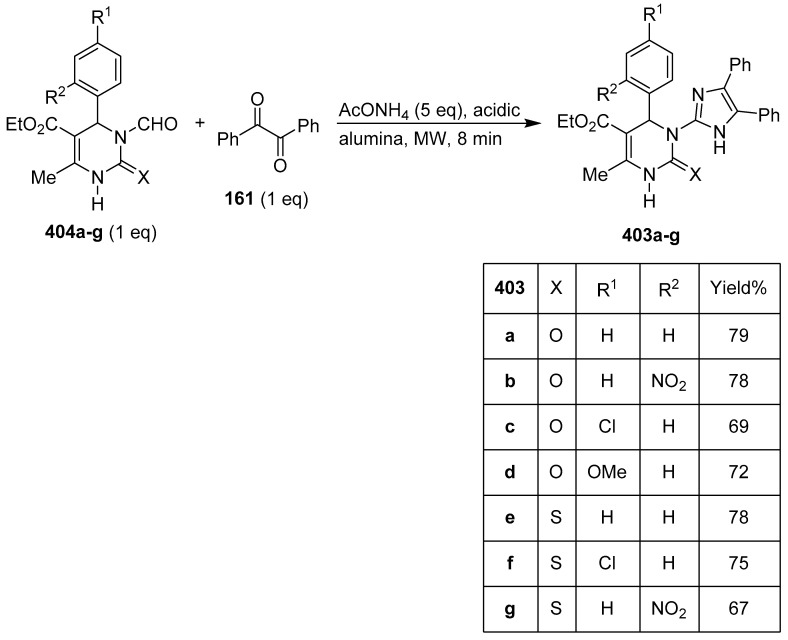
Synthesis of imidazolyl pyrimidines **403a**–**g.**

In 2011, Rathod synthesised substituted ethyl 3-(4,5-diphenyl-1*H*-imidazol-2-yl)-6-methyl-2-oxo/sulfanylidene-4-phenyl-1,2,3,4-tetrahydropyrimidine-5-carboxylates **403a**–**g** by condensation of benzil (**161**) with substituted ethyl 3-formyl-6-methyl-2-oxo/sulfanylidene-4-phenyl-1,2,3,4-tetrahydropyrimidine-5-carboxylates **404**, and AcONH_4_ in the presence of acidic alumina and glacial AcOH under reflux for 12 h using conventional heating (Method A) or under microwave irradiation for 8 min (Method B) (Scheme 82) [232]. The yields of the required imidazolyl pyrimidines, which were higher using this second method, ranged from 67 to 79%.

The antibacterial activity of compounds **403a**–**g** was assayed against *S.* Typhi, *P. aeruginosa*, *K. pneumoniae*, and *S. aureus* using norfloxacin as the standard drug. Among the tested compounds at 100 µg/mL, **403d** with a zone of inhibition of 12 mm was the most active against *S. aureus*. It was also observed that *S.* Typhi was highly sensitive to **403b** (zone of inhibition = 12 mm), and that **403f** exhibited maximum zone of inhibition (18 mm) against *K. pneumoniae* and minimum zone of inhibition against all other bacterial strains tested [232].

In 2014, Desai and coworkers described the synthesis of hybrid molecules **405** containing pyrimidine-based imidazole scaffolds and tested the antibacterial activity of these *N*-(4-arylidene-5-oxo-2-sulfanyl-4,5-dihydro-1*H*-imidazol-1-yl)-6-methyl-2-oxo-4-phenyl-1,2,3,4-tetrahydropyrimidine-5-carboxamides against *E. coli* and *S. aureus* [233].

**Scheme 83:**
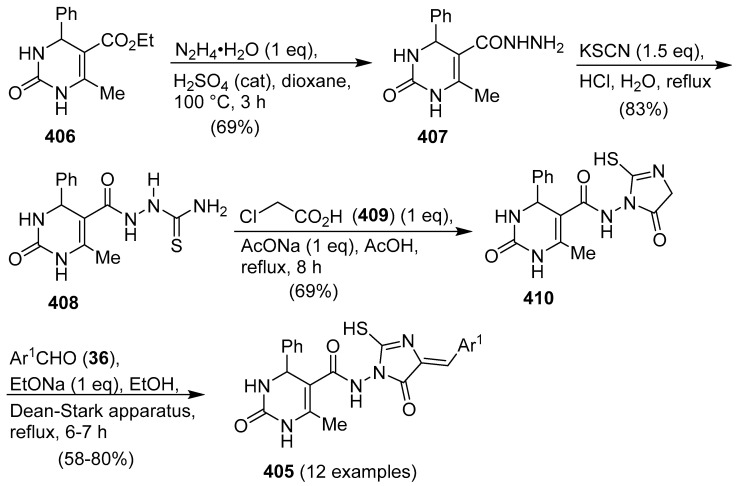
Synthesis of hybrids **405.**

The synthesis of hybrids **405** was achieved as outlined in Scheme 83 starting from ethyl 6-methyl-2-oxo-4-phenyl-1,2,3,4-tetrahydropyrimidine-5-carboxylate (**406**), which was prepared by reaction of benzaldehyde with urea and ethyl acetoacetate in dioxane under reflux in the presence of conc. HCl. The reaction of **406** with hydrazine hydrate in dioxane in the presence of a catalytic amount of conc. H_2_SO_4_ at 100 °C gave compound **407**, which was treated with KSCN in acidic medium, affording carbothioamide **408**. The subsequent reaction of **408** with chloroacetic acid (**409**) and AcONa in AcOH under reflux provided compound **410**, which was eventually converted into the required hybrids **405** by a Knoevenagel-type condensation reaction with the appropriately substituted aryl aldehydes **36** in EtOH in the presence of EtONa. The reaction, which was carried out using a Dean-Stark apparatus, provided hybrids **405** with yields ranging from 58 to 80%. All these hybrids were presumed to mainly possess a *Z*-configuration [233].

The results of an antibacterial screening of compounds **405**, in which ciprofloxacin was used as the standard drug, showed that (i) both **405b** (Ar^1^ = 2-FC_6_H_4_) and **405d** (R = 2-HOC_6_H_4_) exhibited good activity against E. coli and that the antimicrobial activity of hybrids **405f** (Ar^1^ = 4-ClC_6_H_4_) and **405j** (Ar^1^ = 2,6-Cl_2_C_6_H_3_) (MIC = 53 and 50 μM, respectively) against E. coli was lower than that of ciprofloxacin (75 μM); (ii) hybrid **405j** had an MIC value of 25 μM against *S. aureus* that was, on a molar basis, six times lower than that of ciprofloxacin (150 μM) [233].

Finally, in 2016, Pathan and Rahatgaonkar [234] repeated the synthesis of imidazole/pyrimidine hybrids **403a**–**g**, the preparation of which had been reported for the first time in 2011 by Rathod (cf. Scheme 82) [232], and studied the docking of these compounds into the active site of cytochrome P450 (14DM) 14α-sterol demethylase CPY51 enzyme. Hybrids **403a**, **403b**, **403c**, **403d**, which was the most active hybrid against *S. aureus*, and **403f** were found to show the highest binding with P450 (14DM) in comparison to selective drug fluconazole [234].

## 16. Addendum

In recent years, some antibacterial molecular hybrids and conjugates bearing imidazole moiety, which have not been mentioned in the previous sections of this review, have been identified, synthesised, and evaluated for their bioactivity. The literature data of some of these compounds have been summarised below.

**Scheme 84:**
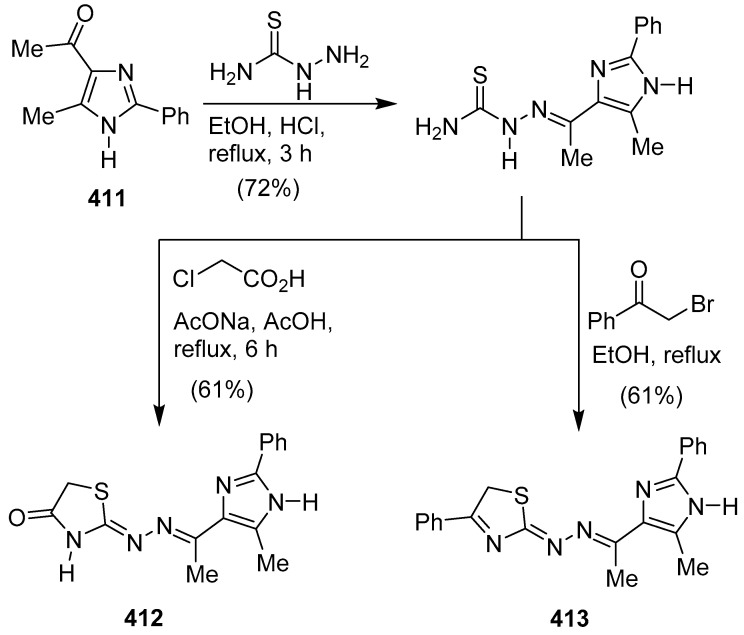
Synthesis of imidazole-based heterocycles **412** and **413.**

In 2011, Abdel-Wahab and coworkers [235] used 1-(5-methyl-2-phenyl-1*H*-imidazol-4-yl) ethan-1-one (**411**) [236] as a precursor for the synthesis of (*Z*)-2-{[(*E*)-1-(5-methyl-2-phenyl-1*H*-imidazol-4-yl)ethylidene]hydrazinylidene}thiazolidin-4-one (**412**) and (*Z*)-2-{[(*E*)-1-(5-methyl-2-phenyl-1*H*-imidazol-4-yl)ethyilidene]hydrazinylidene}-4-phenyl-2,5-dihydrothiazole (**413**). The reactions used for the synthesis of compounds **412** and **413** are shown in Scheme 84.

These imidazole-based heterocycles proved to exhibit strong antibacterial activities against *B. subtilis* ATCC 6633 and *S. aureus* ATCC 29213, and hybrid **412** turned out to exhibit strong activity (MIC = 70 µM) also against *K. pneumoniae* ATCC 13883 [235].

**Figure 13:**
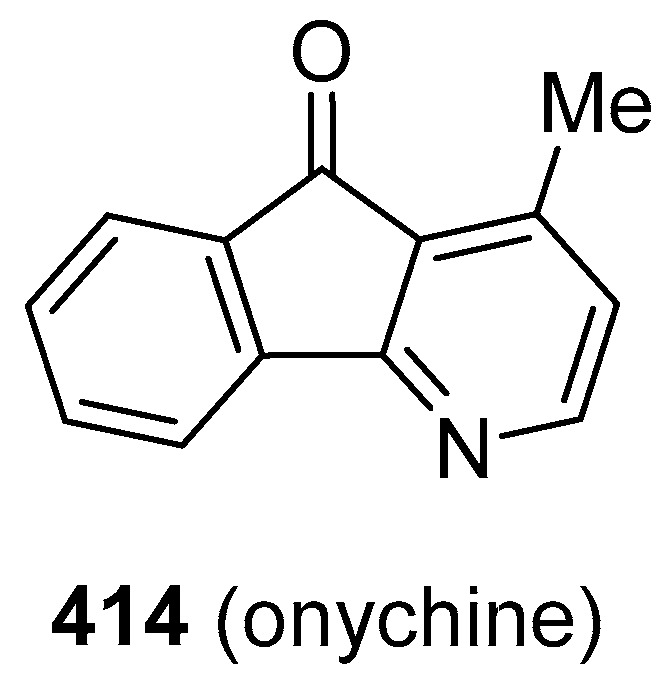
Structure of onychine (**414**).

In 2012, Kantevari and coworkers [237], taking into account that onychine (**414**) (Figure 13), a naturally occurring 4-azafluorenone (5*H*-indeno[1,2-*b*]pyridin-5-one) alkaloid isolated from the root of the plant *Polyalthia debilis* (Pierre) Finet & Gagnep. [238], exhibits antimicrobial activity against *S. aureus*, *B. subtilis*, and *E. coli* (MIC ≥ 500 μM) [239], synthesised 1-benzyl-2-butyl-4-chloroimidazole–embodied 4-azafluorenone hybrids **415**.

**Scheme 85:**
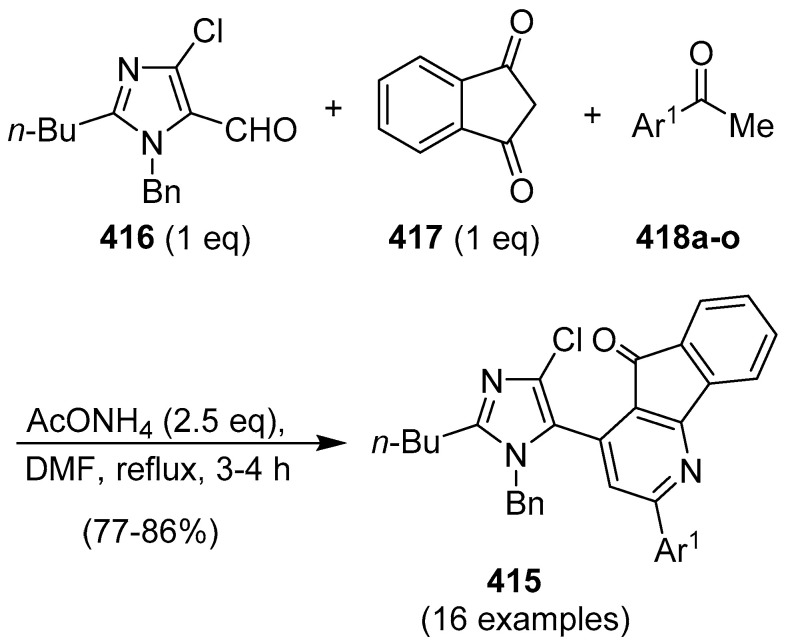
Synthesis of 2-aryl-4-(1-benzyl-2-butyl-4-chloro-1*H*-imidazol-5-yl)-5*H*-indeno [1,2-*b*]pyridin-5-ones **415.**

These compounds were prepared in good yields by one pot condensation of 1-benzyl-2-butyl-4-chloro-1*H*-imidazole-5-carbaldeyde (**416**) with equimolar amounts of 1,3-indanedione (**417**) and methyl aryl ketones **418**, and 2.5 eq of AcONH_4_ in DMF under reflux for 3–4 h (Scheme 85) [237].

2-Aryl-4-(1-benzyl-2-butyl-4-chloro-1*H*-imidazol-5-yl)-5*H*-indeno[1,2-*b*]pyridin-5-ones **415** were then screened for their antibacterial activity against the Gram-positive bacteria *B. subtilis* and *S. aureus* and the Gram-negative bacteria *E. coli*, *P. aeruginosa*, and *K. pneumoniae*, and it was found that compounds **415a** (Ar^1^ = Ph), **415c** (Ar^1^ = 4-BrC_6_H_4_), **415f** (Ar^1^ = 3,4,5-(MeO)_3_C_6_H_2_), **415g** (Ar^1^ = 1-naphthyl), **415h** (Ar^1^ = 2-naphthyl), **415j** (Ar^1^ = 5-Br-2-thienyl), **415k** (Ar^1^ = 2-furyl), **415l** (Ar^1^ = 5-Br-2-pyridyl), and **415n** (Ar^1^ = 2-dibenzothienyl) exhibited good activity towards *S. aureus*. Furthermore, compound **415k** proved to display the highest zone of inhibition against *S. aureus* (15 mm), *P. aeruginosa* (14 mm), and *K. pneumoniae* (15 mm), while the standard drug gentamicin had inhibition zones of 15, 15, and 19 mm, respectively, against these bacterial strains [237].

**Scheme 86:**
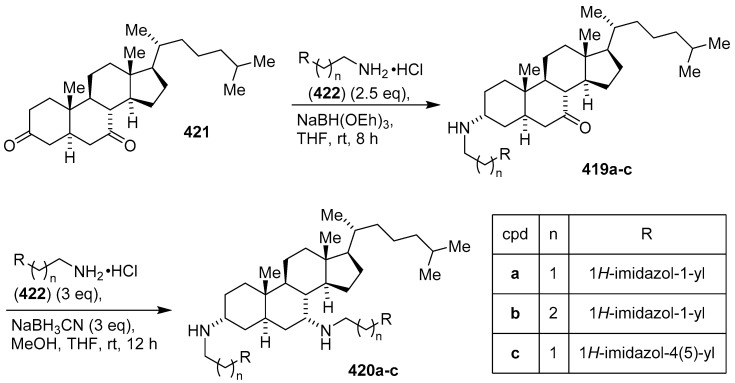
Synthesis of imidazole appended cholestane based-conjugates **419a**–**c** and **420a**–**c.**

In 2013, Kim and coworkers synthesised 3α-amino-5α-cholestane derivatives **419a**–**c** containing imidazole rings and 3α,7α-diamino-5α-cholestane derivatives **420a**–**c** containing imidazole rings starting from 5α-cholestane-3,7-dione (**421**) and evaluated the antimicrobial activity of these imidazole appended cholestane based-conjugates against a range of Gram-positive and Gram-negative bacterial strains [240]. The synthesis of conjugates **419** and **420** was carried out as illustrated in Scheme 86.

Conjugates **419** were prepared by the one-step reductive amination of **421** with the appropriate primary amines hydrochlorides **422**, Et_3_N, and sodium tris(2-ethylhexyloxy)borohydride [NaBH(OEh)_3_] in THF at room temperature. On the other hand, conjugates **420** were then obtained by treatment of compounds **419** with 3 eq of the appropriate amine hydrochlorides **422** and 3 eq of NaBH_3_CN in a 1:1 mixture of THF and MeOH at room temperature [240].

All the conjugates thus synthesised were evaluated for their antibacterial activities and it was found that compounds **419a**–**c** showed activity against most of the Gram-positive bacteria and that 3-propylimidazole conjugate **419b** was four times potent against *S. aureus* 77 and *B. subtilis* ATCC 6633 than compound **419a**, whereas the latter compound exhibited two and four times higher potency against *S. aureus* 241 and *E. faecalis* ATCC 29212 strains, respectively, than compound **419b**. Notably, 3,7-di(imidazole) conjugates **420a**–**c** exhibited antimicrobial potency higher than imidazole conjugates **419a**–**c**. In fact, compound **420b** with MIC values from 4 to 8 μM showed 2–8 times higher activity than compound **419b** against all Gram-positive bacteria (*S. aureus* ATCC 6538P, *S. aureus* giorgio (a methicillin-sensitive strain), *S. aureus* 77, *S. aureus* 241, *S. epidermidis* 887E, *E. faecalis* ATCC 29212, *M. luteus* ATCC 9341, *Bacillus subtilis* ATCC 6633, and *B. licheniformis* EMR). However, conjugates **419** and **420** did not exhibit significant activity against Gram-negative bacteria, which were resistant to these compounds [240].

**Scheme 87:**
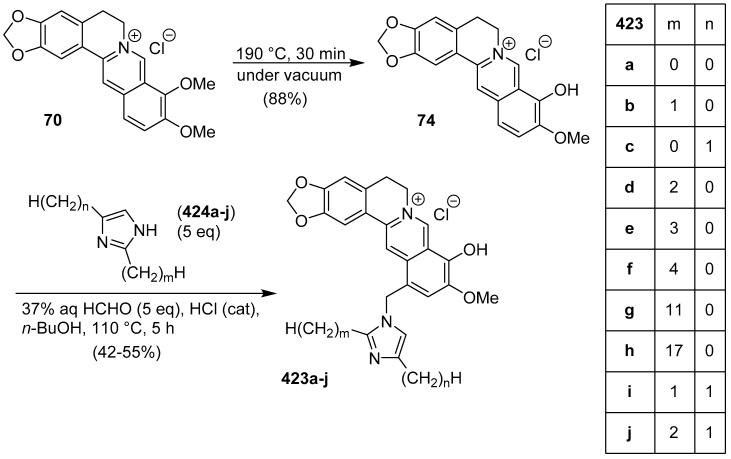
Synthesis of C-12-substituted berberine/imidazole hybrids **423a**–**j.**

In 2016, Zhou and coworkers [241], considering that berberine (**70**), an isoquinoline alkaloid isolated from *C. chinensis* and *Berberis* spp., is a versatile drug in treatment of common metabolic diseases [242] and is a broad spectrum antimicrobial agent [45,243,244,245], synthesised and characterised berberine-derived imidazoles **423a**–**j** and evaluated their in vitro antibacterial activity against the Gram-negative bacteria *E. coli*, *P. hauseri* [60], *P. aeruginosa*, and *S.* Typhi [217] and the Gram-positive bacteria MRSA, *S. aureus*, *B. subtilis*, and *M. luteus*. As shown in Scheme 87 the synthesis of hybrids **423** was carried out by the rarely reported structural modification of the C-12 position in berberine backbone [246,247]. In particular, selective demethylation of berberine chloride (**70**) by heating at 190 °C under vacuum for 0.5 h provided berberrubine chloride (**74**) in 88% yield. The subsequent reaction of 5 eq of imidazoles **424a**–**j** with 5 eq of formaldehyde aqueous solution and 1 eq of **74** in anhydrous *n*-butanol at 110 °C for 5 h in the presence of a catalytic amount of HCl afforded the required hybrids **423a**–**j** in yields ranging from 42 to 55%.

Most of these hybrids turned out to exhibit good bioactivity against the tested bacteria and especially compound **423a** with an MIC value of 2 μM against *S.* Typhi [217] was found to be much more active than the reference drugs berberine (1.4 mM), chloramphenicol [50] (100 μM), and norfloxacin (13 μM). In this study it was also revealed that hybrid **423a** could effectively intercalate into calf thymus DNA to form **423a**–DNA complex, which might further block DNA replication to exert the powerful antimicrobial activities [241].

**Scheme 88:**
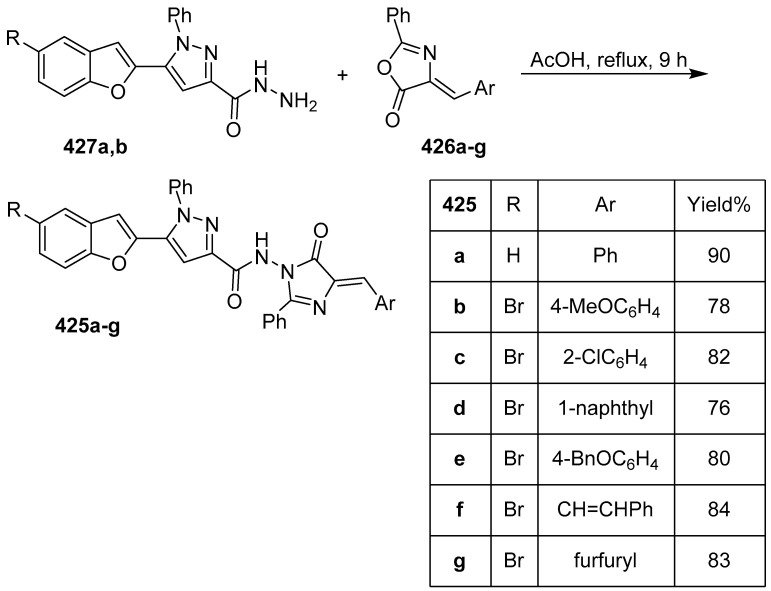
Synthesis of *N*-(4-aryilidene-5-oxo-2-phenyl-4,5-dihydro-1*H*-imidazol-1-yl)-5-(benzofuran-2-yl)-1-phenyl-1*H*-pyrazole-3-carboxamides **425a**–**g.**

Still in 2016, Idrees and coworkers synthesised and evaluated the antibacterial activity of *N*-(4-aryilidene-5-oxo-2-phenyl-4,5-dihydro-1*H*-imidazol-1-yl)-5-(benzofuran-2-yl)-1-phenyl-1*H*-pyrazole-3-carboxamides **425a**–**g** [248]. These compounds were synthesised in 76–90% yield by treatment of 4-arylidene-2-phenyloxazol-5(4*H*)-ones **426** with an equimolar amount of 5-(benzofuran-2-yl)-1-phenyl-1*H*-pyrazole-3-carbohydrazides **427** in AcOH under reflux for 9 h (Scheme 88).

Compounds **425** were tested for their antimicrobial activity in vitro at different concentrations against Gram-positive bacteria *Bacillus thuringiensis* and *S. aureus* and Gram-negative bacteria *E. coli* and *Enterobacter aerogenes*. Chloramphenicol was used as a standard drug. It was found that compound **425a** at a concentration of 230 μM showed excellent activity against *B. thuringiensis* (zone of inhibition = 18 mm) and moderate activity against *S. aureus* (zone of inhibition = 12 mm) and against *E. coli* (zone of inhibition = 15 mm). However, this compound was inactive against *E. aerogenes* at concentrations lower than 450 µM [248].

**Scheme 89:**
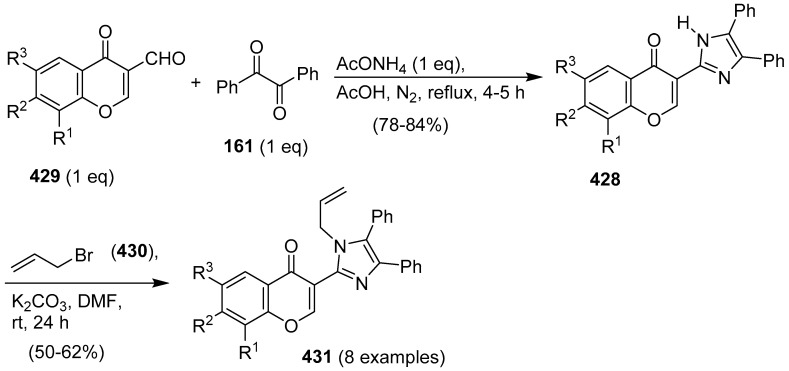
Synthesis of 3-(4,5-diphenyl-1*H*-imidazol-2-yl)-4*H*-chromen-4-ones **428** and 3-(1-allyl-4,5-diphenyl-1*H*-imidazol-2-yl)-4*H*-chromen-4-ones **431.**

In 2017, Ishar and coworkers synthesised and characterised novel 3-(4,5-diphenyl-1*H*-imidazol-2-yl)-4*H*-chromen-4-ones **428** by one pot condensation of substituted 4-oxo-4*H*-chromene-3-carbaldehydes **429**, benzil (**161**), and AcONH_4_ in refluxing AcOH under N_2_ atmosphere (Scheme 89) [249]. The subsequent reaction of compounds **428**, which were obtained in yields ranging from 78 to 84%, with allyl bromide (**430**) in DMF at room temperature for 24 h in the presence of fused K_2_CO_3_ furnished 3-(1-allyl-4,5-diphenyl-1*H*-imidazol-2-yl)-4*H*-chromen-4-ones **431** in yields ranging from 50 to 62%.

Compounds **428** and **431** were then evaluated for their antibacterial activity against Gram-positive *S. aureus* MTCC 96 and *B. subtilis* MTCC 2451, and Gram-negative *E. coli* MTCC 82 and *P. aeruginosa* MTCC 2642. It was thus found that compound **431a** (R^1^ = R^2^ = R^3^ = H) and compound **428c** (R^1^ = R^2^ = H, R^3^ = Cl) exhibited excellent inhibitory activity against *B. subtilis* with MIC values of 2.4 and 3.2 μM, respectively. Compound **431a** also displayed significant inhibitory activity against *E. coli* with an MIC value of 2.8 μM [249].

## 17. Conclusions

Over the past few decades, the growing demand for new antimicrobials for human use that can act against emerging pathogens and multidrug resistant bacterial strains non-treatable by the current antibiotics and responsible for the current increase in morbidity, mortality, and longer hospitalisation, has stimulated scientists to develop a variety of protocols for the synthesis of molecular hybrids and conjugates bearing imidazole moiety and to evaluate the antibacterial activity of these compounds.

In this unprecedented review with 261 references, we have illustrated and commented on the results obtained from the end of 1990s until the end of February 2020 in studies concerning the synthesis, characterisation, and evaluation of the antibacterial activities over 760 non-condensed molecular hybrids and conjugates bearing imidazole moiety, which include natural products such as oroidin (**245a**) [153,154,155,156], clathrodin (**245b**) [152], clathridimine (**228**) [142], and the enantiomer of the first naturally occurring dimeric pyrrole/imidazole hybrid of naturally occurring sceptrin (**213**) [134,135].

As regards the synthetic methodologies used in these studies, it is worth noting that multicomponent condensation reactions, a class of benign processes that are characterised by experimental simplicity and low cost, have frequently been used for the synthesis of a variety of hybrids and conjugates [9,93,97,145,164,166,176,177,178,219,220,232,237], and that such processes [250,251,252,253] as well as several other condensation reactions have often been preferentially carried out using microwave (MW)-promoted reactions instead of conventional heating sources [254,255,256,257,258]. In fact, as often highlighted by the published data, the advantages of microwave-promoted reactions [93,144,166,177,219,232] include significant shorter reaction times, higher yields, and product purity when compared to conventional thermal methods.

Even copper(I)-catalysed alkyne-azide cycloadditions [258,259] have been frequently employed for the efficient introduction of a 1,2,3-triazole moiety into the desired imidazole hybrids and conjugates [32,59,177,178]. On the other hand, classical Pd-catalysed Suzuki-type reactions have only been used for the preparation of imidazole-pyridine fluorophores 2PBI (**336**), 3PBI (**337**), TBPI (**338**), and MPBI (**339**) [203]. Instead, atom economic strategies involving Pd-catalysed regioselective direct (hetero)arylation reactions of heteroaromatics with (hetero)aryl halides or pseudohalides [9,10,11,12,13,14,15], which have been and are still frequently used in the chemistry of imidazole derivatives, as well as the van Leusen reaction based of tosylmethylisocyanides, which is one of the most appropriate strategies for the preparation of imidazole-based medicinal molecules [260], to the best of our knowledge, have never been used for the synthesis of hybrids and conjugates belonging to the classes of substances mentioned in this review.

Many results achieved in the study of the antibacterial properties of the hybrids and conjugates mentioned in this review are also worthy of mention. In fact, numerous compounds, some of which are mentioned below, were proven to exhibit better antibacterial properties than the reference compounds used as standards. In particular, 5-nitroimidazole/3-sulfanyl-1,2,4-triazole hybrids **41c** and **41e** proved to possess MIC values against *C. sporogenes* lower than that of metronidazole (**1**), the reference compound [35]. Nitroimidazole/berberine hybrid **71g** had antibacterial potency against Gram-negative bacteria *S. dysenteriae* and *P. vulgaris* ATCC 6896 superior to that of the reference drugs norfloxacin and chloramphenicol, [44,50]. (*E*)-*N’*-(1-(4-Bromophenyl)-2-(2-methyl-5-nitro-1*H*-imidazol-1-yl)ethylidene)isonicotinohydrazide (**78**) was found to exhibit inhibitory activity against *E. coli* ATCC 35218 that was similar to that of both standards kanamycin B and penicillin G [51]. Quinolone-imidazole hybrid **93i** possessed antibacterial activity against *P. aeruginosa*, one of the ESKAPE pathogens [261], with an MIC value that was significantly lower than that of the reference drugs chloramphenicol, norfloxacin, ciprofloxacin, and clinafloxacin [61]. Naphthalimide-derived metronidazole **112b** proved capable to effectively inhibit the growth of both *P. vulgaris* and *S. dysenteriae*, to kill these bacteria, and to prevent bacterial resistance [65]. Naphthalimide-derived metronidazole **138e** showed high activity against *A. baumannii* (an aerobic Gram-negative ESKAPE pathogen that causes serious infections in the lungs, blood, and brain) with rapid killing effect and no obvious resistance development [74,261]. Imidazole containing bisazetidinones **195a** and **196b** were shown to possess activity against the Gram-negative ESKAPE pathogen *K. pneumoniae* [262] that was almost equivalent to that of ampicillin [121], an antibiotic used to prevent and treat a number of bacterial infections. 1,8-Naphthalimide-derived imidazole **302b** proved to exhibit activity against *E. coli* and *P. aeruginosa* that was comparable to that of the standard drug chloramphenicol [77]. 1,1′-[(Tosylazanediyl)bis(ethane-2,1-diyl)]bis(3-cyanomethyl-1*H*-imidazol-3-ium) chloride (**329c**) was found to be considerably more potent than antibiotic amoxicillin against Gram-positive *B. subtilis* [201]. Quinoline-based hydroxyimidazolium hybrid **398b** demonstrated potent anti-staphylococcal activity and significant activity against *M. tuberculosis* H_37_R_v_ [223]. C-12 Substituted berberine-imidazole hybrid **423a** was proven to exhibit activity against the Gram-negative bacterium *S.* Typhi [217] higher than that of the reference drugs berberine, chloramphenicol, and norfloxacin [241].

**Figure 14:**
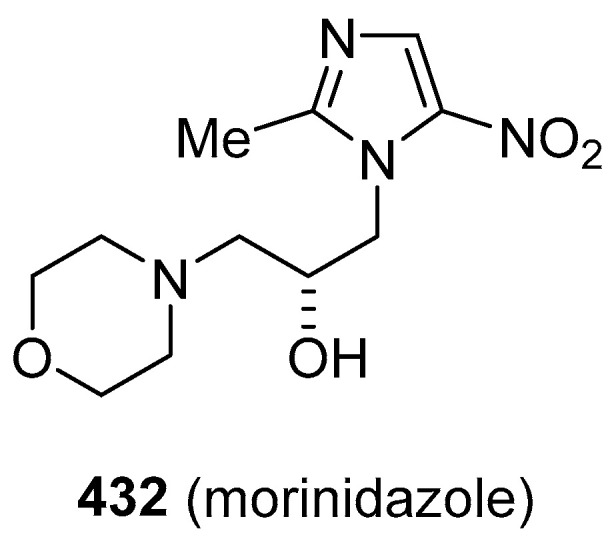
Structure of morinidazole (**432**).

It should, however, be pointed out that, although some brilliant results have been achieved concerning the identification of molecular hybrids and conjugates bearing imidazole moiety that are capable of exercising in vitro effective and selective antibacterial properties also towards multidrug resistant bacteria, to the best of our knowledge none of the compounds mentioned in this review are currently subjected to clinical trials and are included in the list of antibiotics in the clinical pipeline in October 2019 [263]. However, this list contains morinidazole [(R)-1-(2-methyl-5-nitro-1*H*-imidazol-1-yl)-3-(morpholin-4-yl)propan-2-ol] (**432**) (Figure 14), a 5-nitroimidazole antimicrobial drug developed by Jiangsu Hansoh Pharmaceutical and approved in China for the treatment of anaerobic bacterial infections, including appendicitis and pelvic inflammatory disease caused by anaerobic bacteria [264].

Nevertheless, it is our belief that in the near future the current intense research activity in the field of this review [265] can allow to identify and develop novel imidazole-based antibacterial hybrids running into clinical trials and eventually for patient treatment. We hope that the data of this review will pave the way for innovative achievement in this field.

## Abbreviations

Ac	acetyl
Ar	aryl
ATCC	American Type Culture Collection
Bn	benzyl
Boc	*tert*-butoxycarbonyl
BOM	benzyloxymethyl
Bu	butyl
CAN	cerium(IV) ammonium nitrate
Cbz	*N*-carboxybenzyl
CDI	1,1′-carbonyldiimidazole
CFL	compact fluorescent lamp
CGMCC	China General Microbiological Culture Collection Center
*m*-CPBA	*m*-chloroperoxybenzoic acid
DBU	1,5-diazabicyclo[5.4.0]undec-7-ene
DEAD	diethyl azodicarboxylate
DIBAL-H	diisobutylaluminum hydride
DIPEA	*N*,*N*-diisopropylethylamine
DMSO	dimethyl sulfoxide
DPIX	deuterioporphyrin
DMF	dimethylformamide
ED_50_	half effective dose
EDC	1-ethyl-3-(3-dimethylaminopropyl)carbodiimide
Eh	2-ethylhexyl
eq	(stoichiometric) equivalent
Et	ethyl
FDA	Food and Drug Administration
Fmoc	fluorenylmethyloxycarbonyl
HOBt	hydroxybenzotriazole
HBTU	*O*-(benzotriazol-1-yl)-*N*,*N*,*N*’,*N*’-tetramethyluronium hexafluorophosphate
HMTA	hexamethylenetetramine
IC_50_	half inhibitory concentration (bacterial growth or biochemical)
Ir(ppy)_3_	tris(2-phenylpyridine)iridium
Me	methyl
MIC	minimum inhibitory concentration (bacterial growth)
MRSA	methicillin-resistant *Staphylococcus aureus*
Ms	methanesulfonyl
MTCC	Microbial Type Culture Collection (India)
Mtz	metronidazole
MW	microwave
Nim	5-nitro-1*H*-imidazol-1-yl
NMM	4-methylmorpholine
Nps	nanoparticles
n.r.	not reported
Ns	nosyl (*p*-nitrobenzesulfonyl)
PBP	penicillin binding protein
PCC	pyridinium chlorochromate
Ph	phenyl
PNB	*p*-nitrobenzyl
PNZ	*p*-nitrobenzyloxycarbonyl
Pr	propyl
Pro	proline
PTCC	Persian Type Culture Collection
Py	pyridyl
rt	room temperature
TBAB	tetrabutylammonium bromide
TBS	*tert*-butyldimethylsilyl
TBTU	*N*,*N*,*N*′,*N*′-tetramethyl-*O*-(benzotriazol-1-yl)uronium tetrafluoroborate
Tf	triflyl (trifluoromethanesulfonyl)
TFA	trifluoroacetic acid
THF	tetrahydrofuran
TIPS	trisopropylsilyl
Ts	tosyl (*p*-toluenesulfonyl)
